# Towards superior mRNA caps accessible by click chemistry: synthesis and translational properties of triazole-bearing oligonucleotide cap analogs†

**DOI:** 10.1039/d3ra00026e

**Published:** 2023-04-25

**Authors:** Mateusz Kozarski, Karolina Drazkowska, Marcelina Bednarczyk, Marcin Warminski, Jacek Jemielity, Joanna Kowalska

**Affiliations:** a Division of Biophysics, Institute of Experimental Physics, Faculty of Physics, University of Warsaw Pasteura 5 02-093 Warsaw Poland jkowalska@fuw.edu.pl; b Centre of New Technologies, University of Warsaw Banacha 2c 02-097 Warsaw Poland j.jemielity@cent.uw.edu.pl

## Abstract

Messenger RNA (mRNA)-based gene delivery is a powerful strategy for the development of vaccines and therapeutics. Consequently, approaches that enable efficient synthesis of mRNAs with high purity and biological activity are in demand. Chemically modified 7-methylguanosine (m^7^G) 5′ caps can augment the translational properties of mRNA; however, efficient synthesis of structurally complex caps, especially on a large scale, is challenging. Previously, we proposed a new strategy to assemble dinucleotide mRNA caps by replacing the traditional pyrophosphate bond formation by copper-catalyzed azide–alkyne cycloaddition (CuAAC). Here, we used CuAAC to synthesize 12 novel triazole-containing tri- and tetranucleotide cap analogs with the aim of exploring the chemical space around the first transcribed nucleotide in mRNA and overcoming some of the limitations previously reported for the triazole-containing dinucleotide analogs. We evaluated the efficiency of incorporation into RNA for these analogs and their influence on the translational properties of *in vitro* transcribed (IVT) mRNAs in rabbit reticulocyte lysate and JAWS II cultured cells. The incorporation of the triazole moiety within the 5′,5′-oligophosphate of trinucleotide cap produced compounds that were well incorporated into RNA by T7 polymerase while replacing the 5′,3′-phosphodiester bond with triazole impaired incorporation and translation efficiency, despite a neutral effect on the interaction with the translation initiation factor eIF4E. One of the compounds (m^7^Gppp-tr-C_2_H_4_pA_m_pG), had translational activity and other biochemical properties comparable to natural cap 1 structure, thus being a promising mRNA capping reagent for potential in cellulo and *in vivo* applications in the field of mRNA-based therapeutics.

## Abstract 2

mRNA-based gene delivery is a powerful strategy for many therapeutic areas. In this work, we used CuAAC to synthesize next-generation triazole-bearing mRNA 5' cap analogs and evaluated them as reagents for modification of in vitro transcribed mRNA.

## Graphical abstract

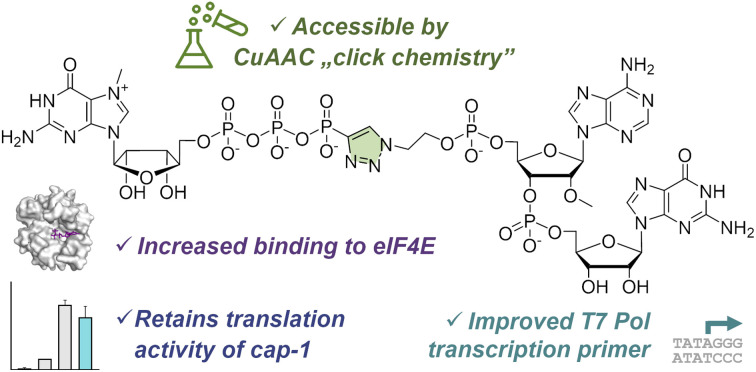

## Introduction

The 5′ end of eukaryotic messenger RNA (mRNA) terminates with a unique structure called the 5′ cap. The 5′ cap consists of a positively charged 7-methylguanosine (m^7^G) linked to the first transcribed nucleotide *via* a 5′,5′-triphosphate bridge.^1^ The 5′ cap plays crucial roles in many processes in eukaryotic cells, including mRNA maturation, transport, and turnover.^2^ Moreover, the 5′ cap protects mRNA against premature degradation by 5′-exonucleases^3^ and facilitates the initiation of protein biosynthesis by enabling recognition of the 5′ end of mRNA by the eukaryotic translation initiation complex 4F (eIF4F), wherein eIF4E is the cap-binding protein.^4^ The 5′ cap also serves as a tag preventing the recognition of RNA by some elements of the innate immune system, thereby enabling the innate immune system to distinguish between cellular and non-self RNA.^2^

m^7^G cap analogs have been used as reagents for modifying the 5′ end of mRNA with the aim of designing more potent mRNA-based therapeutics or molecular tools for biological studies.^5–8^ Chemical modification of the 5′,5′-triphosphate bridge in the m^7^G cap may increase mRNA half-life and its affinity for eIF4E, resulting in higher protein output.^9^ To date, several different mRNA cap analogs have been developed with the aim of improving mRNA product quality and translational properties.^10,11^ However, recent advances in mRNA-based vaccines^12–14^ have highlighted the demand for synthetic m^7^G cap analogs that, besides possessing superior biological properties, can be synthesized in bulk. Chemical synthesis of m^7^G cap analogs is challenging owing to the instability of the positively charged m^7^G under both acidic and alkaline conditions.^15^ Additionally, the usual ZnCl_2_-mediated coupling reaction to form a pyrophosphate bond based on P-imidazolide of 7-methylguanosine diphosphate as the key reagent may be inefficient, time-consuming, and difficult to upscale.^16^ Therefore, new, faster, and more robust approaches for the synthesis of m^7^G cap analogs need to be explored.

**Fig. 1:**
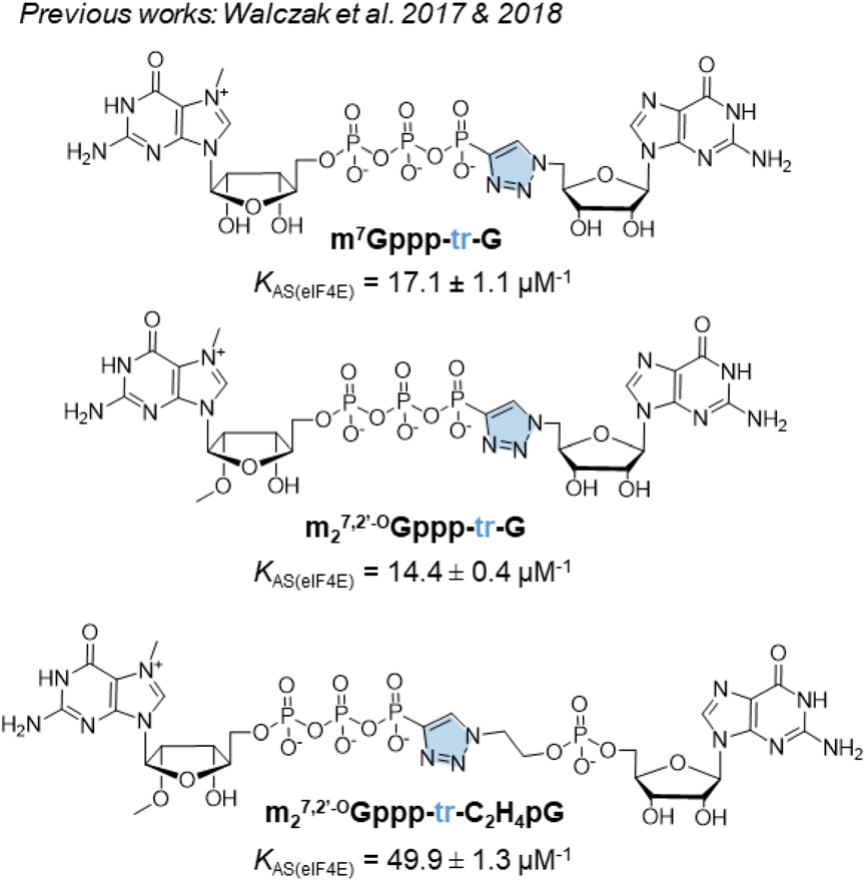
Structures of previously synthesized phosphotriazole dinucleotide cap analogs which have been used as a template in this work.^17,19^

For addressing this problem, we previously synthesized derivatives of the m^7^GpppG dinucleotide cap analog, bearing a triazole modification within the 5′,5′-triphosphate bridge, which can be efficiently assembled from two mononucleotide subunits using copper-catalyzed azide–alkyne cycloaddition (CuAAC).^17^ CuAAC, being the prime example of a ‘click reaction’,^18^ provides shorter reaction times and minimizes the amount of reagents and organic solvents used and by-products generated during mRNA cap synthesis. After screening multiple caps carrying different triazole-containing polyphosphates, we identified compounds that had a high affinity for eIF4E and improved the efficiency of protein biosynthesis *in vitro* and in cultured cells.^17,19^ Unfortunately, we also found several compounds that did not confer favorable translational properties, despite their fairly stable interaction with eIF4E. Notably, the presence of the triazole moiety between the 5′,5′-triphosphate and the 5′ carbon of the first transcribed nucleotide decreased the efficiency of incorporation of the cap analog during transcription initiation, and consequently, decreased capping efficiency of the mRNA product.^17^ Therefore, such compounds were determined to be unsuitable for mRNA-related application. For instance, m^7^Gppp-tr-G (Fig. 1) showed higher affinity for eIF4E (*K*_AS_ = 17.1 ± 1.1 μM^−1^) than that showed by unmodified m^7^GpppG (*K*_AS_ = 12.5 ± 0.3 μM^−1^). Moreover, capping efficiency of m^7^Gppp-tr-G was high (79%); however, because of the location of the triazole ring, the compound incorporated into mRNA in the reverse orientation, resulting in a non-functional product. The anti-reverse analog (ARCA) carrying the same modification, m_2_^7,2′-O^Gppp-tr-G, was free of this limitation, but provided unacceptably low RNA capping efficiency (17%).^17^ Later, an improved version of the triazole-modified dinucleotide cap carrying a triazole-modified tetraphosphate chain, namely m_2_^7,2′-O^Gppp-tr-C_2_H_4_pG, was reported (Fig. 1).^19^ m_2_^7,2′-O^Gppp-tr-C_2_H_4_pG showed high affinity for eIF4E (*K*_AS_ = 49.9 ± 1.3 μM^−1^) and good chemical stability. The efficiency of translation of mRNA capped with m_2_^7,2′-O^Gppp-tr-C_2_H_4_pG was higher than that of mRNA capped with ARCA-capped RNA. However, capping efficiency of m_2_^7,2′-O^Gppp-tr-C_2_H_4_pG was notably lower than that observed with reference caps.^19^

**Fig. 2:**
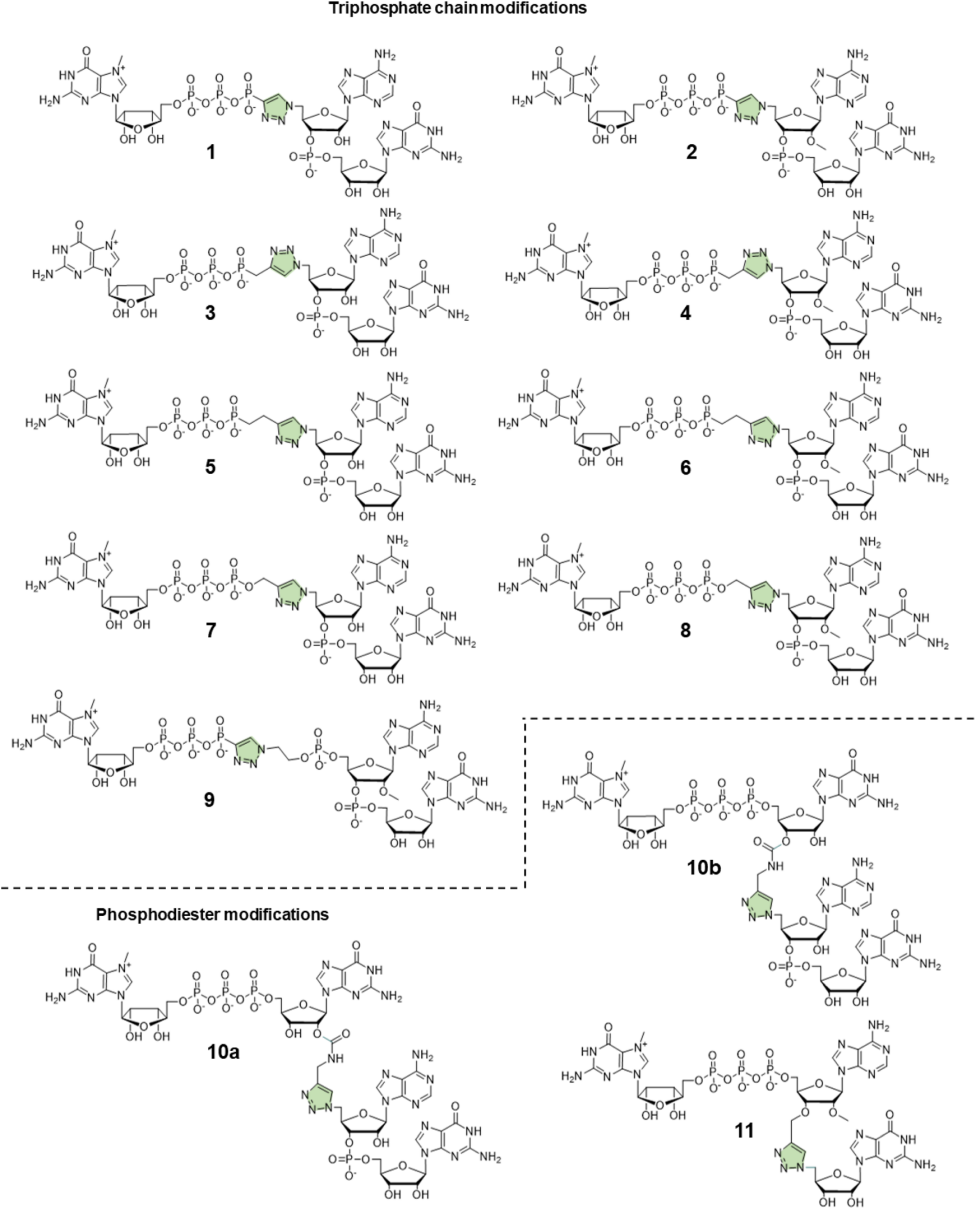
Structures of phosphotriazole trinucleotide cap analogs synthesized in this work.

In this study, to further explore the potential of triazole-modified capping reagents and address some of the previously identified issues, we developed a novel class of triazole-containing tri- and tetranucleotide cap analogs (Fig. 2). Previous studies have shown that trinucleotide cap analogs based on the general structure of m^7^GpppNpG initiate transcription from templates containing class III T7 promoter, similar to dinucleotide derivatives of m^7^GpppG.^7,11,20^ In the case of dinucleotide cap analogs, RNA polymerase initiates transcription by pairing G in the cap and C in the DNA template. In the case of m^7^GppApG-like trinucleotides, the transcription is initiated by pairing between G and C at +1 position and additional pairing between A and T at the −1 position in the DNA template. This additional pairing promotes transcription initiation with the cap analog instead of GTP, increasing capping efficiency and reducing the frequency of incorrect (reverse) cap incorporation into RNA, compared to dinucleotides.^7^ Additionally, the trinucleotide design enables incorporation of epigenetic methylations at the 2′-O position and within the nucleobase of the first transcribed nucleotide, which is impossible with dinucleotide cap analogs. As such, in this study, we explored whether a similar approach can be employed to augment the incorporation of triazole-modified cap analogs into the 5′ end of mRNA. To this end, we developed efficient synthetic pathways for azido- and alkyne-functionalized mono- and dinucleotide subunits, which can be combined in the CuAAC to efficiently form tri- or tetranucleotide caps carrying a triazole moiety within the 5′,5′-oligophosphate bridge or instead of the 5′,3′-phosphodiester bond. The obtained novel cap analogs were incorporated into mRNA encoding *Gaussia* luciferase by *in vitro* transcription, and translational properties of capped transcripts were examined in rabbit reticulocyte lysate (RRL) and in cultured cells (JAWS II) to identify structures that were efficient transcription initiators and conferred superior biological properties to mRNA.

## Results and discussion

### Design and synthesis of novel trinucleotide cap analogs bearing triazole moiety

**Fig. 3:**
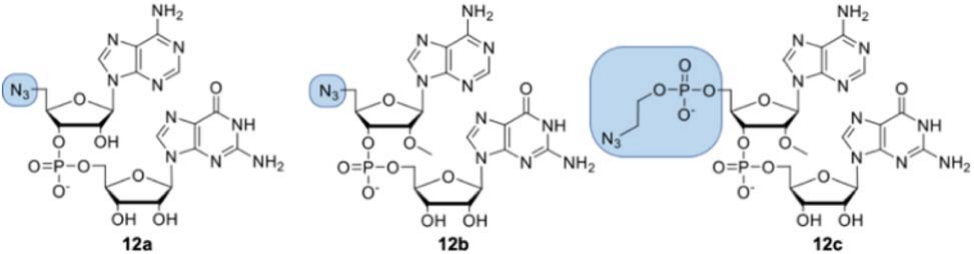
Structures of 5′-azido modified dinucleotides (compounds 12a, 12b, and 12c).

Dinucleotides bearing an azide moiety at the 5′ position of ribose, namely compounds 12a–c, were synthesized through a solid-phase synthesis approach. We synthesized three types of clickable 5′-azidodinucleotides, providing access to cap 0 and cap 1 type RNA ends (Fig. 3).

**Scheme 1:**
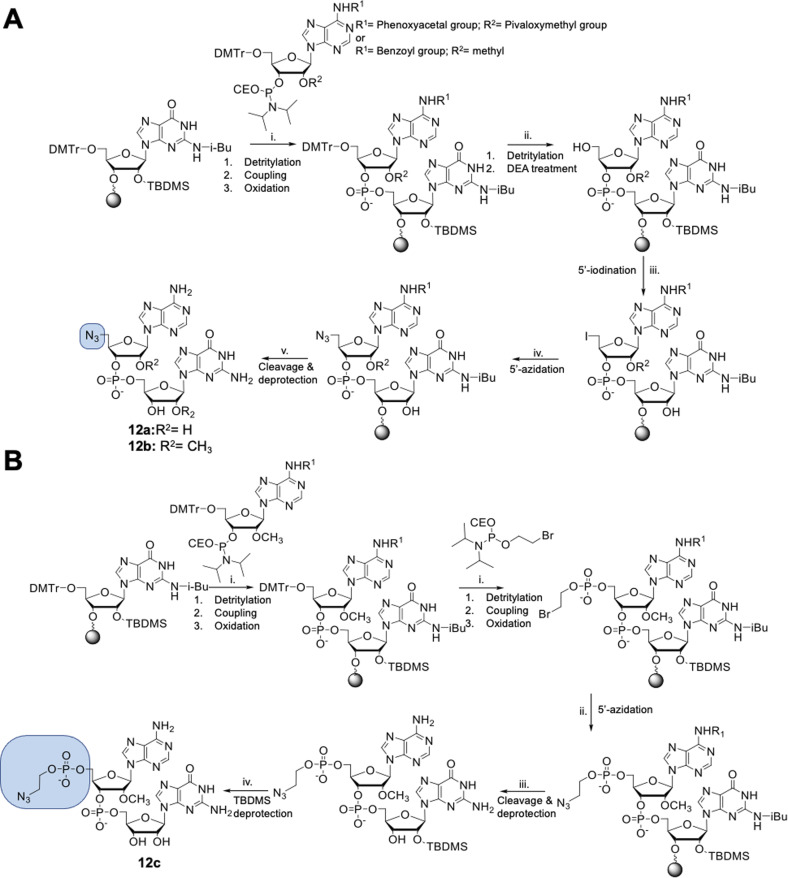
Solid phase synthesis of 5′-azido modified dinucleotides. (A) Solid phase synthesis of compounds 12a and 12b; (i) (1) 3% trichloroacetic acid in dichloromethane, (2) 2 equivalents of phosphoramidites in acetonitrile 0.2 M and BTT Activator 0.3 M, (3) 50 mM iodine solution in pyridine/water (9 : 1, v/v); (ii) (1) 3% trichloroacetic acid in dichloromethane, (2) 20% (v/v) diethylamine in acetonitrile; (iii) 0.6 M (PhO)_3_PCH_3_I in anhydrous DMF, 15 minutes, rt; (iv) saturated solution of NaN_3_ in DMF, 1 hour, 60 °C; (v) 33% ammonium hydroxide and 40% methylamine in water (1/1, v/v), 1 hour, 50 °C; (B) synthesis of compound 12c; (i) (1) 3% trichloroacetic acid in dichloromethane, (2) 2 equivalents of phosphoramidites in acetonitrile 0.2 M and BTT Activator 0.3 M, (3) 50 mM iodine solution in pyridine/water (9 : 1, v/v); (ii) saturated solution of NaN_3_ in DMF, 1 hour, 60 °C; (iii) 33% ammonium hydroxide and 40% methylamine in water (1/1, v/v), 1 hour, 50 °C; (iv) TEA, TEA*3HF, DMSO, 3 h, 65 °C.

**Scheme 2:**
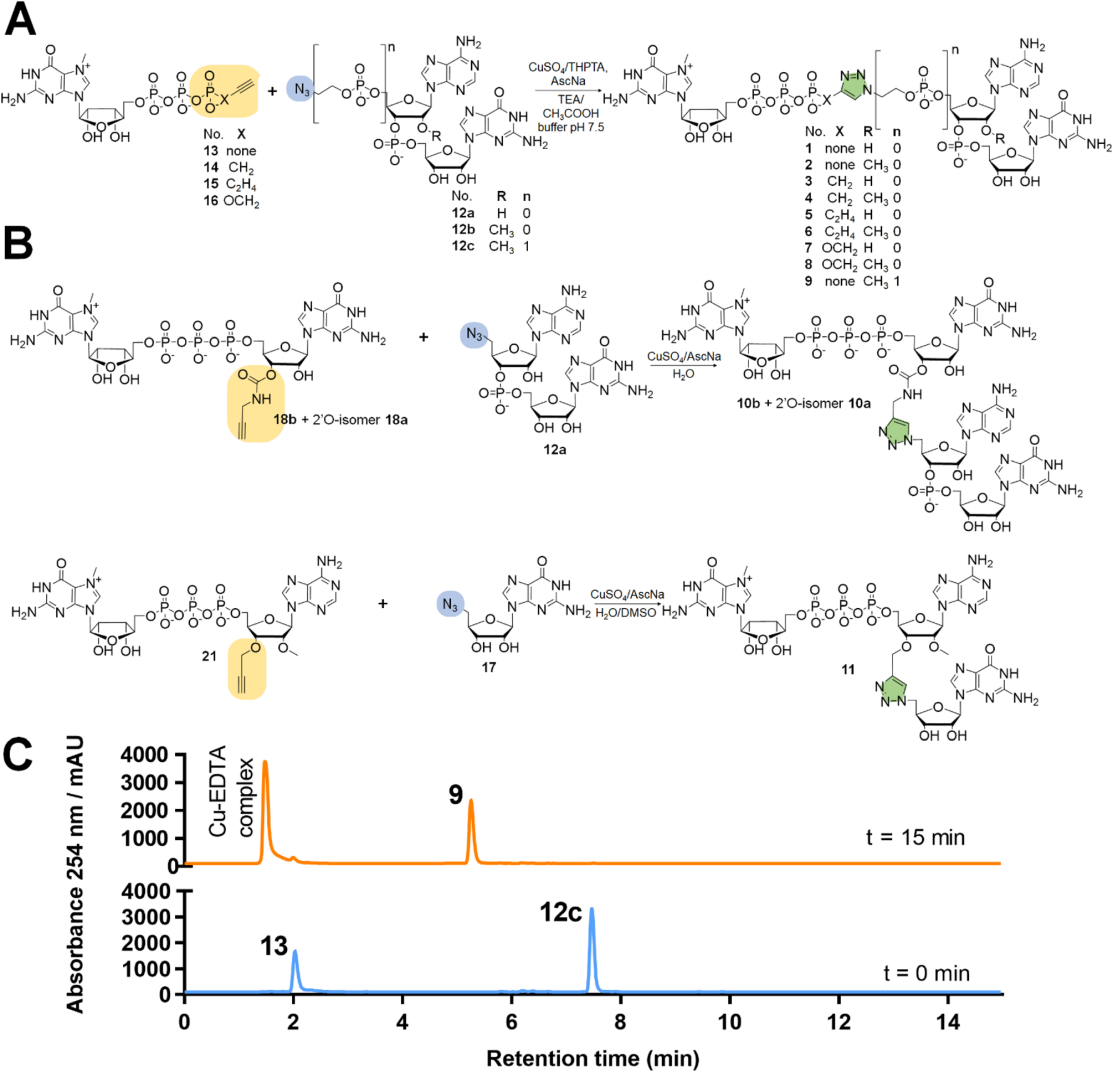
Synthesis of tri- and tetranucleotide cap analogs by CuAAC. (A) Synthesis of trinucleotide cap analogs with phosphate bridge modification. (B) Synthesis of tri- and tetranucleotide cap analogs with phosphodiester modification. (C) Representative RP-HPLC chromatogram obtained for the synthesis of compound 9.

The dinucleotides were synthesized using phosphoramidite approach based on adenosine 2′-*O*-PivOM-phosphoramidites (Scheme 1).^21^ In the case of compounds 12a and 12b, the azido group was incorporated on a solid support by treating the 5′-OH deprotected compound with methyltriphenoxyphosphonium iodide, followed by incubation in a saturated solution of sodium azide in DMF.^22^ After the azidation step, the final dinucleotides 12a and 12b were cleaved from the support, and protecting groups were removed by incubating the compounds in ammonium/methylamine solution (1 : 1 v/v) (Scheme 1A). To synthesize dinucleotide 12c, an additional coupling step with 2-cyanoethyl 2-bromoethyl *N*,*N*-diisopropylamino-phosphite was performed after 5′-OH deprotection. The obtained dinucleotide carrying 2-bromoethyl phosphoester was converted into 2-azidoethyl phosphoester by treatment with NaN_3_ in DMF and cleaved from the resin (Scheme 1B). Subsequently, all dinucleotides were isolated by ion-exchange chromatography as triethylammonium salts and used in CuAAC reactions to obtain triazole-bearing trinucleotide cap analogs. Trinucleotide cap analogs 1–9 were synthesized *via* CuAAC between dinucleotides containing 5′-azido group and m^7^G 5′-triphosphate analogs containing different terminal alkynes at the γ-phosphate.^17,23^ Typically, an aqueous solution of 5′-azidodinucleotide (1 equivalent) was mixed with a 5′-triphosphate 7-methylguanosine analog containing alkyne moiety (1–2 equivalents) dissolved in TEA/CH_3_COOH buffer (pH 7.5), followed by the addition of an aqueous solution of THPTA/CuSO_4_ (0.5 equivalents) and excess sodium ascorbate. The reaction mixture was mixed for 15–60 minutes and quenched with an aqueous solution of Na_2_EDTA (Scheme 2A).

Tri- and tetranucleotide cap analogs in which the phosphodiester bond was replaced with a triazole moiety, namely compounds 10a, 10b, and 11, were synthesized *via* different routes (Scheme 2B). For this purpose, we synthesized 2′- and 3′-alkyne functionalized cap analogs and linked them to 5′-azidonucleoside 17 or dinucleotide 12a*via* CuAAC to obtain functional triazole-bearing tri- or tetranucleotide cap analogs.

To obtain tetranucleotide cap analogs, we used alkyne-functionalized dinucleotides, m^7^GpppG derivative functionalized with carbamoyl moiety at either the 2′ or 3′ position of guanosine, 18a and 18b.^24^ The mixture of 18a and 18b (1 equivalent) was reacted with 5′-azidodinucleotide 12a (1 equivalent) in an aqueous solution to obtain a mixture of compounds 10a and 10b. After CuAAC, compounds 10a and 10b were separated using RP-HPLC (Scheme 2B). Finally, we synthesized a trinucleotide cap analog, compound 11, carrying a 3′-OCH_2_-triazole moiety and methyl group at the 2′ position of adenosine. The starting material was 2′-*O*-methyl-3′-*O*-propargyl-adenosine (compound 19), which was obtained in one step by treating commercially available 2′-*O*-methyl-adenosine with propargyl bromide in the presence of NaH and TBAI.^25^ Compound 19 was converted into 2′-*O*-methyl-3′-*O*-propargyl-adenosine 5′-monophosphate (20) using phosphoryl chloride in trimethyl phosphate.^26,27^ The product was isolated by ion-exchange chromatography and linked with m^7^GDP imidazole-derivative using MgCl_2_-mediated coupling reaction to obtain 3′-alkyne-functionalized m^7^GpppA_m_ (21). Finally, compounds 21 and 17 (5′-azido-5′-deoxyguanosine) were used to synthesize compound 11*via* CuAAC. Compounds 21 and 17 were mixed in dimethyl sulfoxide (DMSO)/water, followed by the addition of copper sulfate (0.3 equivalent) and sodium ascorbate (10 equivalents). The reaction mixture was then stirred for 1 h at 25 °C (Scheme 2B). The reaction progress was monitored using RP-HPLC with an absorption detector (Scheme 2C). The 5′-azido dinucleotides were converted into desired products (compounds 1–9, 10a–b, and 11) in high yields (76–100%), based on RP-HPLC analyses. Triazole-bearing tri- and tetranucleotide cap analogs were isolated from the reaction mixtures by ion-exchange chromatography and by preparative RP-HPLC. The structures and purities of all the compounds were confirmed by HRMS and NMR.

### Evaluation of RNA capping efficiency

**Fig. 4:**
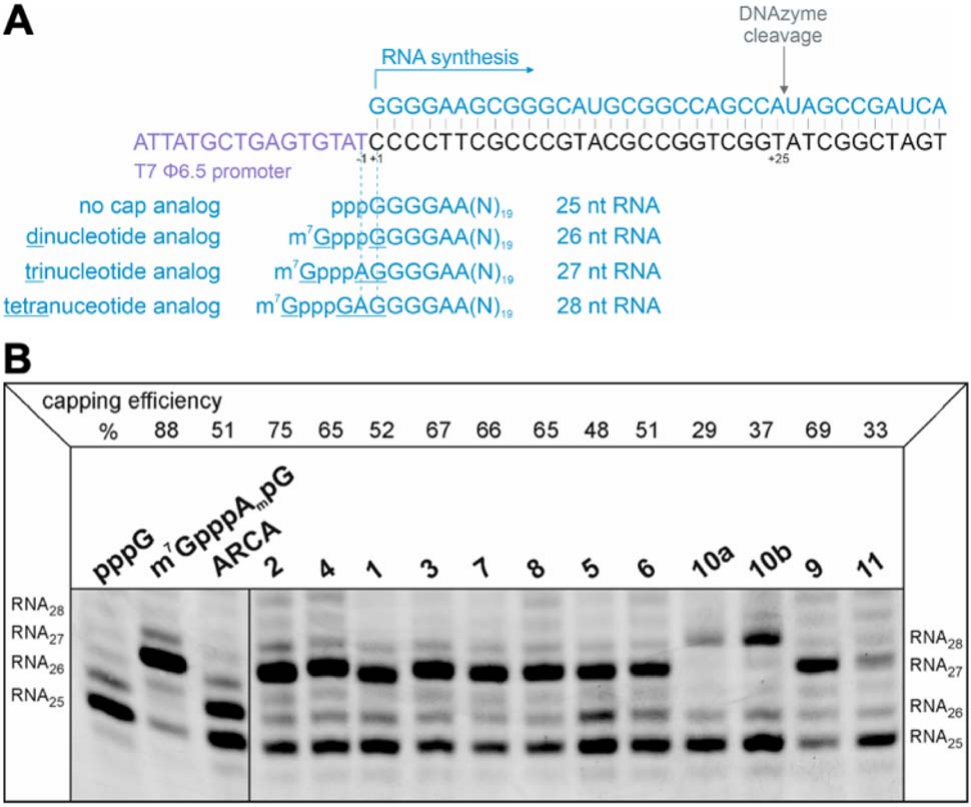
(A) Cap analog pairing with the template sequence which initiates transcription. The particular type of analog incorporated into short RNA, *i.e.*, di-, tri-, or tetranucleotide, defines the length of the final transcript after DNAzyme trimming as 26, 27, or 28 nucleotides, respectively. Control uncapped RNA is 25 nucleotides long. (B) Analysis of capping efficiency. Short RNAs obtained by *in vitro* transcription from a template containing φ6.5 promoter, capped with different analogs during the reaction (with ATP, CTP, UTP, 2 mM each; 0.5 mM GTP; 3 mM cap analog), were analyzed in 15% PAA after trimming by DNAzyme. Capping efficiency values based on densitometric quantification of bands corresponding to capped and uncapped RNAs in each sample are given above the gel.

The efficiency of incorporation of the cap analogs into RNA (capping efficiency) during *in vitro* transcription was assessed. Short RNA molecules were generated by *in vitro* transcription in the presence of cap analogs at concentrations 6-fold higher than GTP. The transcripts were purified by HPLC, treated with DNAzyme to reduce 3′-end heterogeneity, and analyzed in a high-resolution polyacrylamide gel (Fig. 4B). Previously reported di- and trinucleotide cap analogs, ARCA, and m^7^GpppA_m_pG (cap 1), respectively, were used as reference (Fig. 4B). The expected length of uncapped RNA obtained using this protocol was 25 nt. The length of capped RNA varied from 26 to 28 nt, depending on the pairing scheme with the promoter sequence (Fig. 4A).

Capping efficiency for control trinucleotide analog m^7^GpppA_m_pG and dinucleotide analog ARCA was observed to be 88% and 51%, respectively. As expected, control trinucleotide analogs were incorporated into the transcripts more efficiently than control dinucleotide analogs under the same conditions. The capping efficiency of the novel analogs ranged between 29% and 75%. Significantly lower capping efficiencies (compared to compounds 1–9), *i.e.*, 29%, 37%, and 33% were observed for compounds 10a, 10b, and 11, respectively, which carry a triazole moiety in place of the first phosphodiester bond. The lowest capping efficiency was observed for compound 10a, a tetranucleotide, in which triazole moiety was attached to 2′ position of the first transcribed nucleotide. Analog 2 showed the highest capping efficiency (75%) among all the triazole-bearing cap analogs, significantly higher than that of its dinucleotide counterpart (17%).^17^ Relatively high capping efficiency was also observed for compounds 9 (69%), 3 (67%), 7 (66%), 4 (65%), and 8 (65%); consequently, these analogs seemed to be promising molecules for further testing. Since compounds 10a and 11 showed low capping efficiencies, they were excluded from further analysis.

### Exploring the translational properties of mRNA capped with triazole-containing cap analogs

**Fig. 5:**
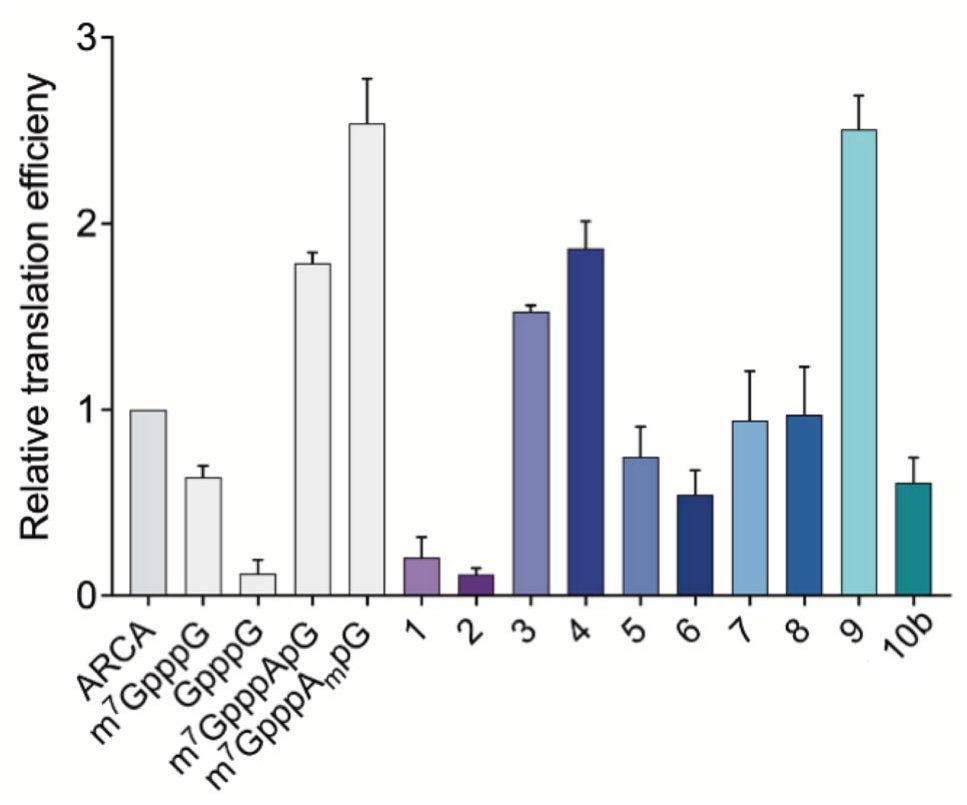
Translation efficiencies for mRNAs capped with novel triazole-bearing trinucleotide cap analogs from IVT reaction with ATP, CTP, UTP, 2 mM each; 0.5 mM GTP; 3 mM cap analog, measured *in vitro* in rabbit reticulocyte lysate system. *Gaussia* luciferase luminescence signal was measured as a function of mRNA concentration and the linear regression coefficients were obtained from those dependencies. The coefficients obtained for each capped-mRNA were normalized to the coefficient obtained for ARCA-mRNA to determine relative translational efficiencies. Four different concentrations of mRNA in translation reactions were tested (see Experimental section for details). mRNAs capped with ARCA and other unmodified m^7^G caps were used as positive controls and GpppG was used as a negative control. Data represent mean value ± SD calculated for three replications.

After assessing the capping efficiencies of the novel triazole-bearing oligonucleotide cap analogs, we explored the translational properties of mRNA carrying them. The typical *in vitro* translation experiment was performed in RRL programmed with mRNA at one of four concentrations (see Experimental section for details). The determined relative translation efficiencies are shown in Fig. 5 and numerical data are shown in Table 1.

**Table 1:** Comparison of *Gaussia* luciferase production from mRNA capped with different cap analogs in RRL and JAWS II living cells

Translational properties of capped mRNA		
Capped-RNA	RRL	JAWS II
	Translation efficiency ± SD	Normalized protein output ± SEM
ARCA (m_2_^7,2′-O^GpppG)	1	1
m^7^GpppG	0.64 ± 0.06	n.d.
pppG	n.d.	0.10 ± 0.09
GpppG	0.12 ± 0.07	n.d.
m^7^GpppApG	1.79 ± 0.06	n.d.
m^7^GpppA_m_pG	2.54 ± 0.24	5.93 ± 0.42
1	0.20 ± 0.11	0.21 ± 0.09
2	0.12 ± 0.03	0.43 ± 0.11
3	1.53 ± 0.03	0.22 ± 0.08
4	1.87 ± 0.14	0.42 ± 0.19
5	0.75 ± 0.16	0.25 ± 0.09
6	0.54 ± 0.13	0.21 ± 0.05
7	0.94 ± 0.27	0.15 ± 0.05
8	0.97 ± 0.26	0.37 ± 0.14
9	2.51 ± 0.18	4.79 ± 0.10
10b	0.61 ± 0.14	0.15 ± 0.07

In the RRL experiment, mRNAs capped with previously reported di- and trinucleotide cap analogs, ARCA, m^7^GpppG, GpppG, and m^7^GpppApG (cap0) m^7^GpppA_m_pG (cap1), were used as references. In contrast to ARCA, m^7^GpppG can be incorporated into RNA in both the reverse and forward orientations, resulting in non-functional and functional mRNA, respectively.^28^ Therefore, translation efficiency of m^7^GpppG-RNA was observed to be 1.3-fold lower than that of ARCA-capped RNA. Relative translation efficiency for GpppG-RNA (negative control) was observed to be 0.11. In contrast to mRNA capped with dinucleotide cap analogs, mRNA capped with trinucleotide cap analogs (m^7^GpppApG and m^7^GpppA_m_pG) were more active in RRL. The translation efficiencies of m^7^GpppApG-capped RNA and m^7^GpppA_m_pG-capped RNA (88% capping efficiency) were 2-fold and 2.5-fold higher, respectively than that of ARCA-capped RNA (51% capping efficiency). Among all the novel triazole-containing cap analogs, the highest translation efficiency (2.51 ± 0.18) was observed for mRNA capped with compound 9, in which the triazole moiety was located within the 5′,5′-tetraphosphate chain between α and β phosphates. The translation efficiency of mRNA bearing this analog was 2.5-fold higher than that of ARCA-capped RNA and was comparable with m^7^GpppA_m_pG-capped mRNA, despite lower capping efficiency. mRNA capped with compounds 1–8, in which the triazole moiety was located at the 5′ position of the ribose, showed lower translation efficiency than that observed for mRNA capped with m^7^GpppA_m_pG. The lowest translation efficiency was observed for mRNAs capped with compounds 1 and 2 (0.20 ± 0.11 and 0.12 ± 0.03) (Table 1), in which the triazole ring was directly connected to the phosphate group. This finding contrasted with the previous finding that the triazole moiety at this position in the dinucleotide does not disturb the interaction of mRNA with eIF4E.^17^ This may be attributed to the conformational rigidity of the modified 5′,5′-triphosphate chain that reveals itself after incorporation into RNA, but not for ‘free’ caps. In agreement with this hypothesis, the insertion of a methylene linker between the triazole and phosphate groups in compounds 3 and 4 significantly increased the translation efficiency of mRNA (1.53 ± 0.03 and 1.87 ± 0.14, respectively). The translation efficiency of mRNAs capped with compounds 3 and 4 was nearly 2-fold higher than that of ARCA-capped RNA and comparable with that of m^7^GpppApG-capped mRNA (1.79 ± 0.06). However, elongating the methylene linker by inserting a second methylene group (compounds 5 and 6) decreased the translation efficiency (0.75 ± 0.16 and 0.54 ± 0.13, respectively), which may be attributed to steric effects combined with slightly lower capping efficiencies of these analogs. In addition, the introduction of a phosphoester group instead of phosphonate (compounds 7 and 8) did not improve the translational properties of capped mRNAs. The translation of transcripts capped with these compounds was comparable with that of ARCA-capped mRNA.

### Protein output from capped mRNAs in JAWS II cells

Protein-production efficiency (output) from mRNAs capped with the triazole-bearing analogs was investigated in JAWS II cells. mRNAs encoding *Gaussia* luciferase were prepared during the IVT reaction utilizing a 6-fold higher cap analog concentration than GTP. *Gaussia* luciferase is secreted outside the cells, which enables convenient luminescence analysis in the collected cell culture medium at different time points without cell lysis.^29^ Because the presence of RNA impurities may significantly affect the outcome of cell-culture experiments with *in vitro* transcribed mRNA,^7^ uncapped impurities were enzymatically removed from the samples (apart from pppG control), followed by HPLC purification to remove dsRNA. mRNAs before and after HPLC purification were analyzed using a TBE agarose gel (Fig. S1A†). To confirm the removal of dsRNAs, we performed dot-blot analysis using a dsRNA-recognizing antibody (Fig. S1B†). The purified mRNAs were used for transfection of JAWS II cells (lipofection), followed by luciferase activity measurement at different time points (16, 40, 64, and 88 h).

**Fig. 6:**
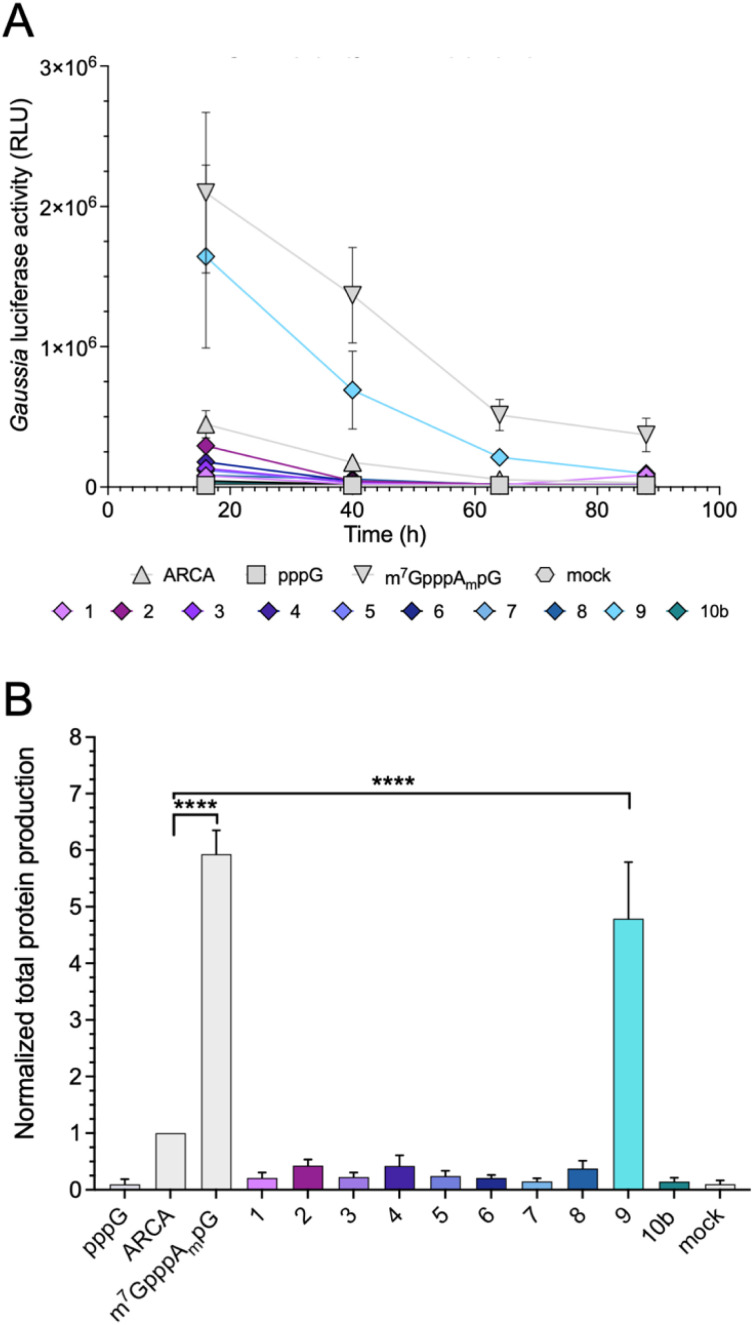
Protein output from HPLC-purified mRNAs capped with various analogs. (A) *Gaussia* luciferase activity was measured in medium collected from JAWS II cultures at particular-time points: 16, 40, 64, and 88 h after cell transfection with luciferase mRNAs capped with various analogs. Medium was replaced with fresh one; therefore, results from each time point show activity of newly produced luciferase. Measurements for technical triplicates from single biological replicate are presented. (B) Total protein production (cumulative luminescence) over four days in JAWS II cells transfected with mRNA capped with various analogs (results from tetraplicate experiment). Bars represent mean value ± SEM normalized to ARCA-capped RNA. Statistical significance: ****P < 0.0001 (one-way ANOVA with Turkey's multiple comparison test).

mRNAs capped with triazole-bearing cap analogs 1–9 and 10b were evaluated. The time-dependent expression is shown in Fig. 6A (representative results from a single replicate), whereas the cumulative luminescence calculated as the sum of all four time points (overall protein output) is shown in Fig. 6B (results from four biological replicates). Surprisingly, the results of the cell culture experiment did not correlate quantitatively with results from the studies in RRL. A significant difference was particularly visible for mRNA capped with compound 4. The translation efficiency of mRNA capped with compound 4 in RRL was 2-fold higher than that of ARCA-capped RNA, whereas, in living cells, mRNA capped with compound 4 was approximately 50% less active than ARCA-capped RNA. Overall, the majority of the triazole-bearing analogs showed notably lower protein production in JAWS II cells than the reference mRNAs. However, mRNA capped with compound 9 was an exception. Protein output from mRNA capped with compound 9 was comparable to that of m^7^GpppA_m_pG-capped RNA. Notably, compound 9 had also the highest translation efficiency in the RRL.

### The affinity for eIF4E and susceptibility to degradation by the hDCP1/DCP2 complex

**Fig. 7:**
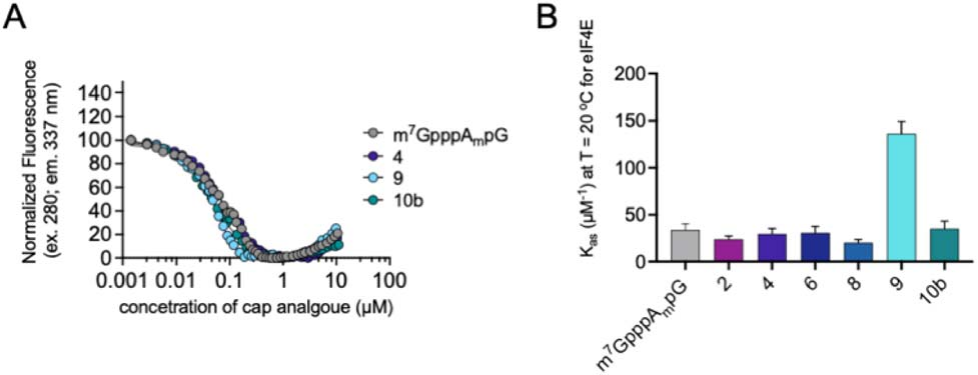
The affinities for eIF4E determined using FQT experiments. (A) Example curves obtained for triazole cap analogs. (B) Cumulative *K*_AS_ values for 2′-*O*-methyl triazole cap analogs.

Interestingly, as explained previously, we observed a divergence between protein expression in RRL and living JAWS II cells, especially for mRNA capped with compound 4. To determine a plausible explanation for the discrepancies, we tested the biochemical properties of the selected compounds (2, 4, 6, 8, 9 and 10b). First, we determined the affinities for eIF4E of triazole-bearing trinucleotide cap analogs, namely compounds 2, 4, 6, 8, and 9, tetranucleotide cap analog 10b and compared them to the affinity of m^7^GpppA_m_pG. To that end, we conducted a fluorescence quenching titration experiment, wherein a decrease in protein fluorescence emission was observed upon binding of the cap analogs to eIF4E.^30,31^ We found that the *K*_AS_ values of nearly all the triazole-bearing cap analogs were comparable with or slightly lower that the *K*_AS_ value determined for m^7^GpppA_m_pG, with the exception of compound 9 (*K*_AS_ = 136.1 ± 13.2 μM^−1^). *K*_AS_ value for compound 9 was more than 4-fold higher than that for m^7^GpppA_m_pG (*K*_AS_ = 29.6 ± 2.3 μM^−1^) (Fig. 7 and Table S2†). This was expected because of the presence of an additional, negatively-charged phosphate group in compound 9.^32^ Overall, the results suggest that increased affinity of triazole-bearing cap analog for eIF4E, as observed for compound 9, is a necessary factor for achieving high translational activity of capped-mRNA.

**Fig. 8:**
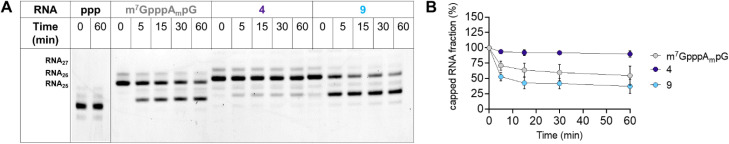
Susceptibility to degradation of capped RNAs by hDCP1/DCP2 complex. (A) Representative PAGE analysis; RNA_25_: uncapped RNA. RNA_27_ – RNA capped with m^7^GpppA_m_pG, compound 4, or compound 9. RNA_26_ – RNA_27_ decapped by hDCP1/2. (B) Comparison of degradation of RNA capped with m^7^GpppA_m_pG, 4, and 9 in time. Data represents mean value ± SD calculated for two replicates.

To further characterize compound 9, we also tested the susceptibility of 27-nt capped RNA to degradation by the human DCP1/DCP2 complex (hDCP1/DCP2). To this end, we synthesized short, 27-nt RNAs capped with compound 9, compound 4, which relatively high translational affinity in RRL but not in JAWS II cells, and m^7^GpppA_m_pG, which was used as a reference (see Experimental section for details). The synthesized RNAs were incubated with hDCP1/DCP2 complex at 37 °C and reaction progress was analyzed at different time points (0, 15, 30, and 60 min) by polyacrylamide gel-electrophoresis (PAGE; Fig. 8A).

We found that RNA capped with analog 4 was completely resistant to degradation by the hDCP1/DCP2 complex under the experimental conditions. Surprisingly, RNA capped with analog 9, which was efficiently expressed in both the RRL system and JAWS II living cells, was degraded faster than the reference (m^7^GpppA_m_pG-capped RNA). These findings juxtaposed with the determined binding affinities for eIF4E, and translational properties of mRNAs capped with 4 and 9, suggest that for the triazole-modified compounds affinity for eIF4E and conformational flexibility may have greater influence on translational properties of mRNA in living cells than susceptibility to hydrolysis by hDCP1/DCP2.

## Conclusions

Herein, we combined a ‘click-chemistry’-based approach to nucleotide assembly with tri- and tetranucleotide-based mRNA capping strategies to achieve an alternative approach to assembly of oligonucleotide cap analogs conferring high translational activity to mRNA. We successfully combined trinucleotide mRNA capping technology, enhancing both capping efficiency and protein production in cultured cells, with CuAAC-based assembly, which may facilitate synthesis of mRNA cap analogs in bulk. Overall, we synthesized 12 triazole-bearing mRNA cap analogs (compounds: 1–9, 10a, 10b and 11) in 15–62% yield. Evaluating the capping efficiencies for all the synthesized cap analogs revealed that analogs bearing the triazole moiety within the 5′,5′-phosphate bridge were relatively well incorporated into short mRNA (50–75% capping efficiency), compared to the unmodified reference (88%), whereas modification within the first phosphodiester bond severely impaired incorporation. Further studies identified compound 9 (m^7^Gppp-tr-C_2_H_4_pA_m_pG) as the cap analog that provided superior properties to mRNA in terms of affinity for eIF4E and translational properties *in vitro* (in RRL system) and in cultured cells (JAWS II). mRNA capped with compound 9 showed biological properties comparable with mRNA capped with unmodified cap 1 (m^7^GpppA_m_pG) commercially available as Clean Cap AG®. Therefore, analog 9, which bears a triazole modification within the oligophosphate bridge, is a promising candidate for further evaluation as an mRNA capping reagent in cultured cells and *in vivo* models for application in mRNA-based therapeutics. Furthermore, due to synthetic accessibility *via* click chemistry, this structural design may be useful for the development of strategies for post-transcriptional chemical capping of mRNA.

## Experimental section

### Chemical synthesis

#### General procedure (A) for solid phase synthesis of dinucleotides

Dinucleotides were obtained according to a previously described protocol.^21^ The dinucleotides were synthesized using an ÄKTA™ Oligopilot™ Plus 10 Synthesizer (GE Healthcare) with 50 μmol of 5′-*O*-DMT-2′-*O*-TBDMS-rGiBu 3′-lcaa Primer Support 5G (GE Healthcare; *ca.* 301 μmol g^−1^ loading). In the coupling step (2 equivalents, 100 μmol) of adenosine phosphoramidite 5′-*O*-DMTr-2′-*O*-PivOM-APAC or 5′-*O*-DMTr-2™-*O*-methyl-A-bz was used. Next, 0.30 M 5-(benzylthio)-1-*H*-tetrazole in acetonitrile was recirculated through the column for 15 min. A solution of 3% (v/v) dichloroacetic acid in toluene was used as detritylation reagent, 0.05 M iodine in pyridine was used for oxidation, 20% (v/v) *N*-methylimidazole in acetonitrile was used as Cap A, and a mixture of 40% (v/v) acetic anhydride and 40% (v/v) pyridine in acetonitrile was used as Cap B. After the last cycle of synthesis, RNAs attached to the solid support were treated with 20% (v/v) diethylamine in acetonitrile to remove 2-cyanoethyl protecting groups. Finally, the solid support was washed with acetonitrile and flushed with argon.

#### 5′-Iodination

The 5′-OH-dinucleotides while still attached to the solid support were converted into 5′-iodo-dinucleotides. To that end, the support was washed with 0.6 M solution of methyltriphenoxyphosphonium iodide dissolved in dry DMF four times, according to the previously described protocol.^21^ After the iodination step, the dinucleotides attached to the solid support were washed with DMF (10 mL) and acetonitrile (10 mL) and flushed with argon to dry the resin.

#### 5′-Azidation

5′-Iodo-dinucleotides were converted to 5′-azido-dinucleotides by nucleophilic substitution. The dry resin was suspended in a saturated solution of NaN_3_ in DMF (5 mL), followed by incubation for 1 h at 60 °C. Subsequently, the resins were washed with DMF (10 mL), water (10 mL), ethanol (10 mL), and acetonitrile (10 mL). After the washing steps, the solid supports with 5′-azido-dinucleotides were flushed with argon. After the 5′-azidation step, final products were cleaved from the solid support by incubating the resins in a solution of 33% ammonium hydroxide and 40% methylamine (1 : 1, v/v) for 1 h at 55 °C. The obtained suspension was filtered off, and the resin was washed with water. The aqueous fractions were combined and freeze-dried. The final products were isolated by ion-exchange chromatography using a DEAE Sephadex™ column, wherein the final products were eluted with a linear gradient of triethylammonium bicarbonate (TEAB) in deionized water (0–0.6 M). 2′-*O*-TBDMS deprotection was omitted because all acidic protecting groups were removed during the 5′-iodination step.

#### Synthesis of 5′-N_3_ApG (compound 12a)

Compound 12a was synthesized according to the general procedure (A) for solid-phase synthesis of dinucleotides with 5′-iodiation and 5′-azidation steps.

^1^H NMR: *δ*_H_ (500.24 MHz; D_2_O; TSP) 8.19 (1H, s), 8.08 (1H, s), 7.91 (1H, s), 5.92 (1H, d, *J* = 4.3, H1′), 5.78 (1H, d, *J* = 4.6, H1′), 4.75–4.73 (1H, m, H2′), 4.68–4.62 (2H, m, H2′, H3′), 4.51–4.47 (1H, m, H3′), 4.37–4.33 (1H, m, H4′), 4.32–4.24 (1H, m, H4′, H5′), 4.20–4.14 (1H, m, H5′′), 3.66 (1H, dd, *J* = 13.7, 3.2, H5′), 3.60 (1H, dd, *J* = 13.7, 5.1, H5′′), 3.19 (9H, q, *J* = 7.4, TEA), and 1.27 (14H, t, 7.4, TEA). ^31^P NMR: *δ*_P_ (202.49 MHz, D_2_O, H_3_PO_4_) −0.73 (1P, s). HRMS (ESI−) calculated for [C_20_H_23_N_13_O_10_P^−^]: 636.1434; *m*/z found: 636.1437.

#### Synthesis of 5′-N_3_A_m_pG (compound 12b)

Compound 12b was synthesized according to the general procedure of solid phase synthesis of dinucleotides with 5′-iodination and 5′-azidation steps.

^1^H NMR: *δ*_H_ (500.24 MHz; D_2_O; TSP) 8.25 (1H, s), 8.14 (1H, s), 7.93 (1H, s), 6.04 (1H, d, *J* = 4.7, H1′), 5.82 (1H, d, *J* = 4.9, H1′), 4.76–4.72 (1H, m, H2′), 4.54–4.48 (2H, m, H2′, H3′), 4.32 (1H, dd, *J* = 8.5, 4.7, H3′), 4.22–4.18 (2H, m, H4′), 3.59 (2H, dd, *J* = 4.3, 2.9, H5′, H5′′), 3.49 (3H, s, CH_3_), 3.57–3.51 (1H, m, H5′), and 3.46–3.41 (1H, m, H5′′). ^31^P NMR: *δ*_P_ (202.49 MHz; D_2_O; H_3_PO_4_) −0.15 (1P, d, *J* = 8.4). HRMS (ESI−) calculated for [C_21_H_25_N_13_O_10_P^−^]: 650.1590; *m*/*z* found: 650.1599.

#### Synthesis of 5′-N_3_-C_2_H_4_pA_m_pG (compound 12c)

Compound 12c was synthesized according to the general procedure of solid phase synthesis of dinucleotides, followed by coupling with 2-cyanoethyl 2-bromoethyl *N*,*N*-diisopropylamino-phosphite, oxidation with 0.05 iodine in pyridine and DEA treatment. In the second coupling step 2.5 equivalents, 125 μmol 2-cyanoethyl 2-bromoethyl *N*,*N*-diisopropylamino-phosphite were used.^33^

#### 5′-Azidation of 5′-Br-C_2_H_4_pA_m_pG

The azido group was substituted by suspending the solid-support 5′-bromo dinucleotide in a saturated solution of sodium azide in DMF. The suspension was shaken for 1 h at 60 °C. Next, the solution was removed by filtration, and the resin was washed with DMF, water, ethanol, and acetonitrile and dried under reduced pressure.

#### Cleavage and TBDMS deprotection of 5′-N_3_-C_2_H_4_pA_m_pG (compound 12c)

5′-Azido dinucleotide attached to the solid support was suspended in a solution of 33% ammonium hydroxide and 40% methylamine in water (1 : 1, v/v; 10 mL) and shaken for 1 h at 55 °C. Next, the suspension was filtered using a syringe with frit. The resin was then washed with water (20 mL). The collected dinucleotide fractions were evaporated and freeze-dried to remove ammonia. After freeze-drying, 5′-azido dinucleotide was dissolved in DMSO (250 μL) and heated to 65 °C. Then, triethylamine (125 μL) was added, followed by TEA·3HF (65 μL). The final solution was shaken for 4 h at 65 °C. Next, the pH of the reaction mixture was adjusted to 6–7 with an aqueous solution of 0.05 M NaHCO_3_. 5′-Azido-dinucleotide was isolated by ion-exchange chromatography using a DEAE Sephadex™ column, wherein 5′-azido-dinucleotide was eluted with a linear gradient of TEAB (0–0.6 M).

^1^H NMR: *δ*_H_ (500.24 MHz; D_2_O; TSP) 8.25 (1H, s), 8.41 (1H, s), 8.23 (1H, s), 7.97 (1H, s), 6.10 (1H, d, *J* = 5.6, H1′), 5.86 (1H, d, *J* = 5.5, H1′), 4.96 (1H, ddd, *J* = 8.2, 4.8, 3.6, H3′), 4.55–4.48 (2H, m, H2′, H3′), 4.48–4.43 (1H, m, H4′), 4.37–4.32 (1H, m, H4′), 4.22 (2H, t, *J* = 4.0, H5′, H5′′), 4.06 (1H, dt, *J* = 11.7, 3.6, H5′), 3.98 (1H, ddd, *J* = 11.7, 4.9, 2.6, H5′′), 3.95–3.86 (m, CH_2_), 3.53 (2H, q, *J* = 7.3, EtOH), 3.48 (3H, s, CH_3_), 3.44–3.34 (2H, m, CH_2_), 3.20 (5H, q, *J* = 7.3, TEA), 1.33 (t, *J* = 7.3, EtOH), and 1.28 (t, *J* = 7.3, TEA). ^31^P NMR: *δ*_P_ (202.49 MHz; D_2_O; H_3_PO_4_) −0.14 (1P, s), 0.58 (1P, s). HRMS (ESI−) calculated for [C_23_H_30_N_13_O_14_P_2_^−^]: 774.1516; *m*/*z* found 774.1520.

#### General procedure (B) for the synthesis of trinucleotide m^7^G cap analogs

Trinucleotide m^7^G cap analogs (compounds 1–9) were synthesized *via* CuAAC. An aqueous solution (15–30 mM) of 5′-N_3_ApG (12a), 5′-N_3_A_m_pG (12b), or 5′-5′-N_3_-C_2_H_4_pA_m_pG (12c) (triethylammonium salt) and 1.0–3.0 equivalents of 5′-triphosphates-7-methylguanosine analogs (bearing terminal alkyne group) (compounds 13, 14, 15, and 16) in TEA/CH_3_COOH buffer (pH 7.5) or deionized water were mixed for 10 min at 25–40 °C. Then, 0.2–0.5 equivalents of copper sulfate (CuSO_4_·5H_2_O) dissolved in water or complex of CuSO_4_ and tris(3-hydroxypropyltriazolylmethyl) amine (THPTA) (1 : 1) were added. The reaction was initiated by adding an aqueous solution of sodium ascorbate (10 equivalents). The reaction mixture was shaken for 15–60 minutes at 25 °C or 37 °C. The reaction was quenched by adding an aqueous solution of Na_2_EDTA (1.0–1.5 equivalents). The obtained compounds were purified by ion-exchange chromatography using a DEAE Sephadex™ A-25 column (HCO_3_^−^ form) and by RP-HPLC with a semi-preparative column. The structures of the obtained cap analogs were confirmed by high-resolution mass spectrometry and NMR (1H, 31P, COSY, HSQC).

#### Synthesis of m^7^Gppp-tr-ApG (compound 1)

Compound 1 was obtained according to general procedure (B). 5′-N_3_ApG (12a) (6.5 μmol, 4.9 mg, 1.0 equivalent) was dissolved in deionized water (300 μL) and mixed with 13 (m^7^GpppC_2_H) (6.5 μmol, 3.5 mg, 1.0 equivalent) dissolved in TEA/CH_3_COOH buffer (300 μL; pH 7.5). The reaction was started by addition of aqueous solutions of CuSO_4_/THPTA complex (3.25 μmol, 0.8 mg, 0.5 equiv., 5 μL) followed by addition of aqueous solution of sodium ascorbate (65 μmol, 12.8 mg, 10 equiv., 30 μL). Then, the reaction mixture was shaken for 1 h at 37 °C. The reaction was quenched by adding an aqueous solution of Na_2_EDTA (9.75 μmol, 3.63 mg, 1.5 equivalent) and purified using semi-preparative RP-HPLC (column B and method D) (Table S1†). Compound 1 was obtained as an ammonium salt in 36% yield.

^1^H NMR: *δ*_H_ (399.90 MHz; D_2_O; TSP) 9.13 (1H, s, H8 m^7^G), 8.21 (1H, s, H8), 8.20 (1H, s, H8), 8.18 (1H, s, H2), 8.01 (1H, s, Htriazol), 5.95 (1H, d, *J* = 3.5, H1′), 5.93 (1H, d, *J* = 3.5 Hz, H1′), 5.82 (1H, d, *J* = 4.9, H1′), 5.00–4.87 (1H, m, H3′), 4.68–4.59 (2H, m, H2′ one proton H2′ is overlapped with water), 4.56 (1H, d, *J* = 5.1, H3′), 4.49 (2H, dt, *J* = 15.0, 4.4, H3′, H4′), 4.35 (1H, s, H4′), 4.33–4.12 (7H, m, H5′, H5′′, H4′), and 4.03 (3H, s, CH_3_). ^31^P NMR: *δ*_P_ (161.89 MHz; D_2_O; H_3_PO_4_): −0.74 (1P, s, ApG), −7.94 (d, *J* = 22.2), −11.49 (d, *J* = 19.1), and −23.32 (1P, t, *J* = 20.6, Pβ). HRMS (ESI−) calculated for [C_33_H_40_N_18_O_23_P_4_^−^]: 1181.1548; *m*/*z* found: 1181.1563.

#### Synthesis of m^7^Gppp-tr-A_m_pG (compound 2)

Compound 2 was obtained according to general procedure (B). 5′-N_3_-A_m_pG 12b (3.7 μmol, 2.8 mg, 1.0 equiv.) was dissolved in deionized water (300 μL) and mixed with compound 13 (m^7^Gppp-C_2_H) (3.7 μmol, 2.0 mg, 1.0 equiv.) dissolved in TEA/CH_3_COOH buffer (300 μL; pH 7.5). To start the reaction, an aqueous solution of CuSO_4_/THPTA complex (1.85 μmol, 0.5 mg, 0.5 equiv., 2.5 μL) was added, followed by addition of an aqueous solution of sodium ascorbate (37 μmol, 6.4 mg, 10 equiv., 10 μL). Then, the reaction mixture was shaken for 30 min at 37 °C. The reaction mixture was quenched by adding aqueous solution of Na_2_EDTA (5.55 μmol, 2.1 mg, 1.5 equiv.) and purified using semi-preparative RP-HPLC (column B and method D) (Table S1†). Compound 2 was obtained as an ammonium salt in 25% yield.

^1^H NMR: *δ*_H_ (399.90 MHz; D_2_O; TSP) 9.12 (1H, s, H8 m^7^G), 8.21 (1H, s, H8), 8.19 (1H, s, H8), 8.16 (1H, s, H2), 7.94 (1H, s, Htriazol), 6.02 (1H, d, *J* = 3.7, H1′), 5.94 (1H, d, *J* = 3.7, H1′), 5.80 (1H, d, = 5.2, H1′), 5.00 (1H, dt, *J* = 8.4, 5.7, H3′), 4.61 (1H, dd, *J* = 4.9, 3.7, H2′), 4.56 (1H, dd, *J* = 10.0, 5.7, H3′), 4.50 (dd, *J* = 5.0, 4.8, H4′), 4.46 (1H, t, *J* = 5.0, H3′), 4.39 (1H, dd, *J* = 5.2, 3.7, H2′), 4.36–4.33 (1H, m, H4′), 4.31–4.29 (1H, m, H4′), 4.27–4.12 (5H, m, H5′, H5′′), 4.02 (3H, s, CH_3_ m^7^G), and 3.47 (3H, m, CH_3_, 2′-OMe). ^31^P NMR: *δ*_P_ (161.89 MHz; D_2_O; H_3_PO_4_) −1 (1P, s, ApG), −7.96 (d, *J* = 22.2), −11.49 (d, *J* = 17.3), and −23.35 (1P, t, *J* = 22.0, Pβ). HRMS (ESI−) calculated for [C_34_H_43_N_18_O_23_P_4_^−^]: 1195.1704; *m*/*z* found: 1195.1712.

#### Synthesis of m^7^Gppp-CH_2_-tr-ApG (compound 3)

Compound 3 was obtained according to general procedure (B). 5′-N_3_-ApG (12a) (6.5 μmol, 4.9 mg, 1.0 equiv.) was dissolved in deionized water (300 μL) and mixed with compound 14 (m^7^Gppp-C_3_H_3_) (6.5 μmol, 3.7 mg, 1 equiv.) dissolved in 300 μL TEA/CH_3_COOH (300 μL, pH 7.5). The reaction was started by addition of an aqueous solution of CuSO_4_/THPTA complex (3.25 μmol, 0.8 mg, 0.5 equiv., 5 μL) and an aqueous solution of sodium ascorbate (65 μmol, 12.8 mg, 10 equivalents, 30 μL). After 15 minutes of incubation, an extra portion of compound 14 (6.5 μmol, 3.7 mg, 1.0 equivalent) was added and incubation was continued for 30 minutes. After that, the reaction mixture was quenched by adding an aqueous solution of Na_2_EDTA (9.75 μmol, 3.63 mg, 1.5 equivalents, 500 μL) and purified using semi-preparative RP-HPLC (column B and method D) (Table S1†). Compound 3 was obtained as an ammonium salt in 57% yield.

^1^H NMR: *δ*_H_ (399.90 MHz; D_2_O; TSP) 9.17 (1H, s, H8), 8.21 (1H, s, H8), 8.10 (1H, s, H8), 7.94 (1H, s, H2), 7.77 (1H, d, *J* = 2.2, Htriazol), 5.96 (1H, d, *J* = 3.6, H1′), 5.92 (1H, d, *J* = 3.4, H1′), 5.80 (1H, d, *J* = 4.7, H1′), 4.89 (1H, dt, *J* = 7.8, 5.6, H3′), 4.75 (1H, t, *J* = 5.0, H2′), 4.72–4.66 (3H, m, H2′, H3′, H4′), 4.63 (1H, dd, *J* = 4.9, 3.6, H2′), 4.61–4.54 (1H, m, H4′), 4.50 (2H, q, *J* = 5.1, H3′, H4′), 4.38–4.26 (4H, m, H5′,H5′′), 4.25–4.14 (1H, m, H5′′,H5′), 4.03 (3H, s, CH_3_), 3.59 (dd, *J* = 13.2, 7.0), and 3.14 (2H, dq, *J* = 20.5, 15.5, HCH_2_). ^31^P NMR: *δ*_P_ (161.89 MHz; D_2_O; H_3_PO_4_) 10.56 (1P, dd, *J* = 45.3, 24.6), −0.80 (1P, s, ApG), −11.56 (1P, d, *J* = 19.5), and −23.10 (1P, dd, *J* = 25.5, 19.3, Pβ). HRMS (ESI−) calculated for [C_34_H_42_N_19_O_23_P_4_^−^]: 1195.1704; *m*/*z* found 1195.1722.

#### Synthesis of m^7^Gppp-CH_2_-tr-A_m_pG (compound 4)

Compound 4 was obtained according to the general procedure (B). 5′-N_3_-A_m_pG 12b (3.7 μmol, 2.8 mg, 1.0 equiv.) was dissolved in 300 μL deionized water and mixed with 14 (m^7^Gppp-C_3_H_3_) (3.7 μmol, 2.1 mg, 1.0 equiv.) dissolved in 300 μL TEA/CH_3_COOH pH 7.5 buffer. Then, an aqueous solution of CuSO_4_/THPTA complex (1.85 μmol, 0.5 mg, 0.5 equiv., 2.5 μL) was added, followed by addition of an aqueous solution of sodium ascorbate (37 μmol, 6.4 mg, 10 equiv., 10 μL). The reaction mixture was then shaken at 37 °C for 30 min. After 30 minutes, the reaction mixture was quenched by adding aqueous solution of Na_2_EDTA (5.55 μmol, 2.1 mg, 1.5 equiv.) and purified by semi-preparative RP-HPLC (column C and method F) (Table S1†). Compound 4 was obtained as an ammonium salt in 29% yield.

^1^H NMR: *δ*_H_ (399.90 MHz; D_2_O; TSP) 9.17 (1H, s, H8 m^7^G), 8.23 (1H, s, H8), 8.12 (1H, s, H8), 7.95 (1H, s, H2), 7.74 (1H, d, *J* = 2.1, HTriazol), 6.04 (1H, d, *J* = 3.5, H1′), 5.97 (1H, d, *J* = 3.6, H1′), 5.82 (1H, d, *J* = 5.0, H1′), 5.02 (1H, dt, *J* = 8.1, 5.6, H3′), 4.68 (2H, d, *J* = 4.8, H2′, H3′), 4.64 (1H, dd, *J* = 4.9, 3.7, H2′), 4.56–4.47 (5H, m, H2′, H3′, H4′), 4.36–4.30 (3H, m, H5′, H5′′), 4.26–4.15 (3H, m, H5′, H5′′), 4.03 (3H, s, CH_3_), 3.49 (3H, s, CH_3_ 2′-OMe), and 3.22–3.05 (2H, m, CH_2_). ^31^P NMR: *δ*_P_ (161.89 MHz; D_2_O; H_3_PO_4_) 10.39 (1P, q, *J* = 22.4), −1.04 (1P, s, ApG), −11.56 (1P, d, *J* = 19.5), and −23.10 (1P, dd, *J* = 25.1, 19.5, Pβ). HRMS (ESI−) calculated for [C_35_H_45_N_18_O_23_P_4_^−^]: 1209.1861; *m*/*z* found 1209.1865.

#### Synthesis of m^7^Gppp-C_2_H_4_-ApG (compound 5)

Compound 5 was obtained according to general procedure (B). 5′-N_3_-ApG 12a (5.5 μmol, 4.0 mg, 1.0 equivalent) was dissolved in deionized water (300 μL) and mixed with 15 (m^7^Gppp-C_4_H_5_) (5.5 μmol, 2.0 mg, 1 equivalent) dissolved in TEA/CH_3_COOH buffer, pH 7.5 (50 μL). Then an aqueous solution of CuSO_4_ (2.2 μmol, 0.35 mg, 0.4 equiv., 3 μL) was added, followed by addition of an aqueous solution of sodium ascorbate (55 μmol, 11 mg, 10 equiv., 30 μL). The reaction mixture was shaken for 1 h at 25 °C. After 1 h of incubation at 25 °C, the extra portion of compound 15 (2 μmol, 1.45 mg, 0.4 equivalents) was added and incubation was continued for 10 minutes. Then, the reaction mixture was quenched by adding an aqueous solution of Na_2_EDTA (8.25 μmol, 3 mg, 1.5 equiv.) and purified by semi-preparative RP-HPLC (column B and method D) (Table S1†). Compound 5 was obtained as ammonium salt in 41% yield.

^1^H NMR: *δ*_H_ (399.90 MHz; D_2_O; TSP) 8.07 (1H, s), 7.93 (1H, s), 7.77 (1H, s), 7.53 (1H, s), 7.84 (1H, s, Htriazol), 5.91–5.89 (2H, m, H1′), 5.80 (1H, d, *J* = 4.5, H1′), 4.89 (1H, s), 4.74–4.69 (2H, m, H2′, overlapped with water), 4.64 (1H, dd, *J* = 5.1, 2.1, H2′), 4.61–4.58 (1H, m), 4.55 (3H, s), 4.49 (2H, dd, *J* = 10.2, 5.3), 4.36–4.30 (4H, m), 4.23–4.16 (2H, m), 4.02 (3H, s, CH_3_), 2.81–2.70 (2H, m, CH_2_), and 2.05–1.91 (2H, m, CH_2_, overlapped with acetone). ^31^P NMR: *δ*_P_ (161.89 MHz; D_2_O; H_3_PO_4_) 17.20–16.82 (1P, m, Pα), −0.80 (1P, s, 3′P), −11,52 (1P, d, *J* = 18.4, Pγ), and −23.02 (1P, dd, *J* = 25.2, 19.0, Pβ). HRMS (ESI−) calculated for [C_35_H_45_N_18_O_23_P_4_^−^]: 1209.1861; *m*/*z* found 1209.1861.

#### Synthesis of m^7^Gppp-C_2_H_4_-A_m_pG (compound 6)

Compound 6 was obtained according to general procedure (B). 5′-N_3_-A_m_pG 12b (3.7 μmol, 2.8 mg, 1.0 equiv.) was dissolved in deionized water (200 μL) and mixed with 15 (m^7^Gppp-C_4_H_5_) (3.7 μmol, 2.2 mg, 1.0 equiv.) dissolved in TEA/CH_3_COOH buffer, pH 7.5 (100 μL). Then, the aqueous solution of CuSO_4_ (1.5 μmol, 0.5 mg, 0.4 equivalents, 3 μL) was added, followed by addition of aqueous solution of sodium ascorbate (37 μmol, 6.4 mg, 10 equivalents, 10 μL). The reaction mixture was then shaken at 25 °C for 30 min. After 30 minutes, the reaction mixture was quenched by adding aqueous solution of Na_2_EDTA (4.4 μmol, 1.7 mg, 1.2 equivalents, 500 μL) and purified using semi-preparative RP-HPLC (column B and method D) (Table S1†). Compound 6 was obtained as an ammonium salt in 29.3% yield.

^1^H NMR: *δ*_H_ (399.90 MHz; D_2_O; TSP) 9.17 (1H, s, H8 m^7^G), 8.24 (1H, s, H8), 8.10 (1H, s, H8), 7.98 (1H, s, H2), 7.48 (1H, d, *J* = 2.1, HTriazol), 6.08 (1H, d, *J* = 2.1, H1′), 5.98 (1H, d, *J* = 3.5, H1′), 5.82 (1H, d, *J* = 4.9, H1′), 5.08 (1H, dt, *J* = 7.2, 5.0, H3′), 4.69–4.67 (1H, m, H2′), 4.57–4.53 (2H, m, H2′, H3′), 4.50–4.44 (4H, m, H3′, H4′), 4.39–4.34 (3H, m, H5′, H5′′), 4.30–4.24 (2H, m, H5′, H5′′), 4.20–4.12 (1H, m, H5′, H5′′), 4.07 (3H, s, CH_3_), 3.53 (3H, s, CH_3_ 2′-OMe), 2.74–2.68 (2H, m, CH_2_), and 1.99–1.91 (2H, m, CH_2_). ^31^P NMR: *δ*_P_ (161.89 MHz; D_2_O; H_3_PO_4_) 17.10–16.50 (1P, m, Pγ), −0.95–(−0.93) (1P, m, ApG), −11.45 (1P, d, *J* = 18.7, Pα), and −22.83–(−23.11) (1P, m, Pβ). HRMS (ESI−) calculated for [C_36_H_47_N_18_O_23_P_4_^−^]: 1223.2017; *m*/*z* found 1223.2014.

#### Synthesis of m^7^GpppOCH_2_-tr-ApG (compound 7)

Compound 7 was obtained according to general procedure (B). 5′-N_3_-ApG 12a (2.5 μmol, 1.0 equivalent) was dissolved in deionized water (300 μL) and mixed with 16 (m^7^GpppOC_3_H_3_) (2.5 μmol, 1.5 mg, 1.0 equivalent) dissolved in TEA/CH_3_COOH buffer, pH 7.5 (50 μL). Then, an aqueous solution of CuSO_4_ (1 μmol, 0.25 mg, 0.4 equivalents, 1.4 μL) was added, followed by addition of an aqueous solution of sodium ascorbate (25 μmol, 4.95 mg, 10 equivalents, 25 μL). The reaction mixture was shaken at 25 °C for 1 h. Next, the reaction was quenched by adding an aqueous solution of Na_2_EDTA (3.75 μmol, 1.4 mg, 1.5 equivalents, 500 μL) and purified using semi-preparative RP-HPLC (column B and method D) (Table S1†). Compound 7 was obtained as an ammonium salt in 41% yield.

^1^H NMR: *δ*_H_ (399.90 MHz; D_2_O; TSP) 9.14 (1H, s, H8 m^7^G), 8.16 (1H, s, H8), 8.11 (1H, s, H8), 7.95 (1H, s, C2), 7.84 (1H, s, Htriazol), 5.97 (1H, d, *J* = 3.7, H1′), 5.94 (1H, d, *J* = 2.4, H1′), 5.81 (1H, d, *J* = 4.7, H1′), 5.02–4.97 (1H, m, H3′), 4.89–4.88 (2H, m, CH_2_), 4.77–4.75 (1H, m, H2′, overlapped with water), 4.73 (1H, dd, *J* = 5.2, 2.5, H2′), 4.66 (1H, dd, *J* = 4.9, *J*2 = 3.8, H2′), 4.63–4.62 (2H, m, H3′), 4.54–4.49 (3H, m, H4′), 4.38–4.16 (5H, m, H5′, H5′′), and 4.06 (3H, s, CH_3_). ^31^P NMR: *δ*_P_ (161.89 MHz; D_2_O; H_3_PO_4_) −0.73 (1P, s, ApG), −11.43–(−11.75) (2P, m, Pα, Pγ), and −22.88 (1P, t, *J* = 18.8, Pβ). HRMS (ESI−) calculated for [C_34_H_43_N_18_O_24_P_4_^−^]: 1211.1654; *m*/*z* found 1211.1655.

#### Synthesis of m^7^GpppOCH_2_-tr-A_m_pG (compound 8)

Compound 8 was obtained according to general procedure (B). 5′-N_3_-A_m_pG 12b (9.4 μmol, 1.0 equiv.) was dissolved in 100 μL water and mixed with a solution of 16 (m^7^GpppOC_3_H_3_) (15.8 μmol, 1.7 equiv.) dissolved in 100 μL. Then, CuSO_4_ (1.2 μL; 2.8 μmol, 0.7 mg, 0.3 equiv.) and sodium ascorbate (29.2 μL; 94 μmol, 18.6 mg, 10 equiv.) were added. The reaction mixture was vortexed at 25 °C for 30 minutes. After 30 minutes, the reaction was quenched by adding an aqueous solution of Na_2_EDTA (18.8 μmol, 6.9 mg, 2 equiv.) and purified by ion-exchange chromatography using a DEAE Sephadex™ A-25 column (HCO_3_^−^ form) and semi-preparative RP-HPLC (column B and method D) (Table S1†). The final product was obtained as an ammonium salt in 45% yield.

^1^H NMR: *δ*_H_ (399.90 MHz; D_2_O; TSP) 9.14 (1H, s, H8 m^7^G), 8.18 (1H, s, H8), 8.11 (1H, s, H8), 7.95 (1H, s, C2), 7.83 (1H, s, Htriazol), 6.05 (1H, d, *J* = 2.5, H1′), 5.97 (1H, d, *J* = 3.7, H1′), 5.81 (1H, d, *J* = 5.0, H1′), 5.14–5.07 (1H, m, H3′), 4.88 (2H, d, *J* = 5.2), 4.66 (1H, dd, *J* = 4.9, 3.7, H2′), 4.60 (2H, d, *J* = 3.8, H3′), 4.53–4.46 (4H, m, H2′, H4′), 4.40–4.32 (3H, m, H5′, H5′′), 4.31–4.13 (3H, m, H5′, H5′′), 4.05 (3H, s, CH_3_ m^7^G), and 3.51 (3H, s, CH_3_, 2′-OMe). ^31^P NMR: *δ*_P_ (161.89 MHz; D_2_O; H_3_PO_4_) −0.11 (1P, m, 3′P), −10.69 (1P, d, *J* = 18.8, Pα or Pγ), −10.89 (1P, d, *J* = 18.3, Pα or Pγ), and −22.87 (1P, dd, *J* = 18.8, 18.3, Pβ). HRMS (ESI−) calculated for [C_35_H_45_N_18_O_24_P_4_^−^]: 1225.1810; *m*/*z* found 1225.1810.

#### Synthesis of m^7^Gppp-tr-C_2_H_4_pA_m_pG (compound 9)

Compound 9 was synthesized according to general procedure (B). 5′-N_3_-C_2_H_4_pA_m_pG 12c (5.75 μmol, 1 equiv.) was dissolved in 100 μL followed by the addition of 13 (m^7^GpppC_2_H) (5.75 μmol, 1 equivalent). The reaction was initiated by adding an aqueous solution of CuSO_4_ (1.15 μmol, 0.2 equivalent, 10 μL) and sodium ascorbate (57.5 μmol, 10 equivalents, 50 μL). The reaction mixture was stirred for 15 min at 25 °C. Next, the reaction was quenched by adding an aqueous solution of Na_2_EDTA (5.75 μmol, 1 equivalent, 500 μL). Compound 9 was isolated from the reaction mixture using semi-preparative RP-HPLC (column A method D) (Table S1†). The final product was obtained as an ammonium salt in 42% yield.

^1^H NMR: *δ*_H_ (500.24 MHz; D_2_O; TSP) 9.12 (1H, s, H8 m^7^G), 8.38 (1H, s, H8), 8.26 (1H, s, H8, Htriazol), 8.21 (1H, s, H2), 7.97 (1H, s, H8), 6.05 (1H, d, *J* = 5.2, H1′), 5.96 (1H, d, *J* = 3.4, H1′), 4.94 (1H, dt, *J* = 8.3, 4.4, H3′), 4.61 (1H, dd, *J* = 4.8, 3.6, H1′), 4.56 (2H, q), 4.53–4.49 (2H, m), 4.47 (1H, t, *J* = 5.1), 4.44–4.38 (1H, m), and 4.37–4.31 (2H, m). ^31^P NMR: *δ*_P_ (202.53 MHz; D_2_O; H_3_PO_4_) *δ* −0.36 (1P, s), −0.93 (1P, s), −7.46 (1P, d, *J =* 21.6, Pα or Pγ), −11.45 (1P, d, *J =*17.5, Pα or Pγ), −23.14 (1P, t, *J =* 20.3, Pβ). HRMS (ESI−) calculated for [C_35_H_45_N_18_O_24_P_4_^−^]: 1225.1810; *m*/*z* found 1225.1810.

#### Synthesis of m^7^GpppG-carb-CH_2_-tr-ApG (compounds 10a and 10b)

5′-N_3_-ApG 12a (1 equivalent, 3.7 μmol) was dissolved in water (200 μL) and mixed with compounds 18a and 18b (2′ and 3′ isomers) (1 equivalent, 3.7 μmol) dissolved in TEA/CH_3_COOH buffer solution, pH 7 (50 μL). The reaction mixture was mixed for 10 min at 25 °C, followed by adding an aqueous solution of CuSO_4_ (0.5 equivalents, 1.85 μmol, 1 μL) and aqueous solution of sodium ascorbate (10 equivalents, 37 μmol, 30 μL). The reaction mixture was then stirred for 1 h at 25 °C. Next, the reaction mixture was quenched by the addition of an aqueous solution of Na_2_EDTA (1.5 equivalents, 5.5 μmol, 500 μL). The obtained isomers (2′ and 3′) were separated using RP-HPLC with a semi-preparative column (column B and method D) (Table S1†). The final isomers (compound 10a and 10b) were obtained as an ammonium salt in yield 15% and 24%, respectively.

##### Compound 10a

^1^H NMR: *δ*_H_ (500.24 MHz; D_2_O; TSP) 9.02 (s, 1H), 8.20 (s, 1H), 7.94 (s, 1H), 7.89 (s, 1H), 7.81 (s, 1H), 7.63 (s, 1H), 5.88–5.86 (m, 3H), 5.83 (1H, d, *J* = 4.2), 5.72 (1H, d, *J* = 4.1), 5.44 (1H, t, *J* = 5.5), 4.76–4.68 (5H, m), 4.65 (1H, t, *J* = 5.1), 4.62–4.54 (6H, m), 4.52 (1H, d, *J* = 5.6 ), 4.49 (1H, d, *J* = 4.9), 4.45 (2H, t, *J* = 5.0), 4.41–4.21 (15H, m), 4.20–4.11 (3H, m), 4.03 (s, 3H). ^31^P NMR: *δ*_P_ (202.53 MHz; D_2_O; H_3_PO_4_) 0.16 (1P, d, *J* = 9.5, Pα), −10.58 (2P, dd, *J* = 19.4, 12.0, 3′P and Pγ), −22.11 (1P, t, *J* = 19.3, Pβ).

##### Compound 10b

^1^H NMR: *δ*_H_ (500.24 MHz; D_2_O; TSP) 9.05 (1H, s), 8.23 (1H, s), 8.01 (1H, s), 7.96 (2H, s), 7.92 (1H, s), 7.72 (1H, s), 5.93 (1H, d, *J* = 3.0), 5.88 (1H, d, *J* = 3.5), 5.80 (1H, d, *J* = 4.7), 5.73 (1H, d, *J* = 7.2), 5.25 (1H, t, *J* = 10.8), 4.74 (1H, d, *J* = 3.0), 4.72–4.65 (4H, m), 4.57 (3H, dd, *J* = 8.1, 5.0), 4.49 (1H, t, *J* = 5.3), 4.45 (2H, t, *J* = 5.2), 4.43–4.15 (12H, m), 4.02 (3H, s). ^31^P NMR: *δ*_P_ (202.53 MHz; D_2_O; H_3_PO_4_) *δ* 0.23 (1P, d, *J* = 10.6, Pα), −10.54 (2H, dd, *J* = 19.3, 11.3, 3′P and Pγ), −21.99 (1P, t, *J* = 19.2). HRMS (ESI−) calculated for [C_35_H_45_N_18_O_24_P_4_^2−^] 759.1225; *m*/*z* found 759.1229 (compound 10a), 759.1225 (compound 10b).

#### Synthesis of 2′-*O*-Me-3′-*O*-C_3_H_3_-adenosine (compound 19)

2′-*O*-Me-3′-*O*-C_3_H_3_-adenosine (compound 19) was synthesized according to the method described by Jawalekar *et al.* with minor modifications.^25^ 2′-*O*-Me-adenosine (2 g, 7 mmol, 1 equivalent) was dissolved in hot anhydrous DMF (78 mL). Then, the solution was cooled on ice, and sodium hydride NaH (560 mg, 14 mmol, 2 equivalents) and tetrabutylammonium iodine TBAI (542 mg, 1.4 mmol, 0.2 equivalents) were added, followed by the addition of propargyl bromide (2.08 g, 8.4 mmol, 1.2 equivalents, 1.32 mL). The reaction mixture was gradually heated to 55 °C and stirred until no further substrate conversion was observed (8 h; substrate conversion of 40%). Next, the solvent was evaporated to dryness and the product was purified using liquid chromatography on silica gel column (0 to 7% of methanol in dichloromethane). The collected fractions, containing desired product, were evaporated giving 200 mg (0.62 mmol) of compound 19 as a yellow powder (8.9% yield).

^1^H NMR: *δ*_H_ (399.90 MHz; DMSO; TSP) 8.39 (1H, s, ade), 8.14 (1H, s, ade), 7.39 (2H, s, Ade NH_2_), 6.01 (1H, d, *J* = 6.4, H1′), 5.56 (1H, dd, *J* = 7.0, 4.7, H4′), 4.56 (1H, dd, *J* = 6.4, 4.9, H2′), 4.36 (1H, dd, *J* = 4.9, 2.8, H3′), 4.33 (2H, t, *J* = 2.2), 4.16 (1H, q, *J* = 3.3, H4′), 3.70 (1H, ddd, *J* = 12.1, 4.7, 3.7, H5′), 3.58 (1H, ddd, *J* = 12.1, 7.0, 3.4, H5′′), 3.51 (1H, t, *J* = 2.4), and 3.31 (3H, s, 2′-OMe).

#### Synthesis of 5′-monophosphate 2′-*O*-Me-3′-OC_3_H_3_ adenosine (compound 20)

Compound 19 (90 mg, 0.28 mmol, 1 equiv.) was dissolved in 1.4 mL of trimethyl phosphate. The solution was cooled on ice, and phosphoryl chloride was added (167.3 mg, 1.1 mmol, 3 equiv., 0.1 mL). The reaction mixture was then stirred on ice for 2 h. After full conversion of the substrate, the final product was precipitated using diethyl ether, washed with diethyl ether, and dissolved in water. The obtained aqueous solution was neutralized by adding sodium bicarbonate to a pH of 7. The final product was purified using ion-exchange chromatography using a DEAE Sephadex™ A-25 column (HCO_3_^−^ form). Compound 20 was isolated as a triethylammonium salt in 36% yield.

^1^H NMR: *δ*_H_ (500.24 MHz; D_2_O; TSP) (1H, s, ade), 8.26 (1H, s, ade), 6.20 (1H, d, *J* = 5.7, H1′), 4.67–4.61 (2H, m, H2′, H3′), 4.56–4.53 (1H, m, H4′), 4.45 (1H, dd, *J* = 16.0, 2.4, CH_2_), 4.41 (1H, dd, *J* = 16.0, 2.4, CH_2_), 3.51 (1H, q, *J* = 7.3, solvent), 3.47 (3H, s, 2′-Ome), 3.20 (7H, q, *J* = 7.3, TEA), 3.07, and 2.96 (1H, t, *J* = 2.4, C_3_H_3_). ^31^P NMR: *δ*_P_ (161.89 MHz; D_2_O; H_3_PO_4_). HRMS (ESI−) calculated for [C_14_H_17_N_5_O_7_P^−^] 398.0871; *m*/*z* found: 398.0872.

#### Synthesis of m^7^GpppA-2′-*O*-Me-3′-*O*-C_3_H_3_ (compound 21)

Compound 20 (1 equivalent, 46 μmol) and m^7^GDP-Im (2 equiv., 92 μmol) were dissolved in dry DMSO (350 μL). Zinc chloride (10 equiv., 460 μmol) was then added, and the reaction mixture was stirred for 2 h. After 2 h, the reaction mixture was quenched by adding an aqueous solution of Na_2_EDTA (20 mg mL^−1^) and sodium bicarbonate (10 mg mL^−1^). The final product was purified using ion-exchange chromatography using a DEAE Sephadex™ A-25 column (HCO_3_^−^ form) and RP-HPLC with a semi-preparative column (column C method D) (Table S1†). The final product was isolated as ammonium salt in 24% yield.

^1^H NMR: *δ*_H_ (500.24 MHz; D_2_O; TSP) 9.05 (1H, s, C8 m^7^G), 8.46 (1H, s, base), 8.24 (1H, s, base), 6.09 (1H, d, *J* = 6.2, H1′), 5.90 (1H, d, *J* = 3.8, H1′), 4.62 (1H, dd, *J* = 5.3, 3.3), 4.57–4.52 (3H, m, H2′ and H3′), 4.49–4.41 (3H, m), 4.41–4.35 (2H, m), 4.33–4.22 (3H, m), 4.05 (3H, s, CH_3_, m^7^G), 3.42 (3H, s, 2′-OMe), and 2.96 (1H, t, *J* = 2.4, CH). ^31^P NMR: *δ*_P_ (161.89 MHz; D_2_O; H_3_PO_4_) −10.89 (2P, dd, *J* = 19.3, 13.3, Pα, Pγ), and −22.44 (1P, t, *J* = 19.3, Pβ). HRMS (ESI−) calculated for [C_25_H_32_N_10_O_17_P_3_^−^] 837.1165; *m*/*z* found: 837.1166.

#### Synthesis of m^7^GpppA_m_-3′-OCH_2_-tr-G (compound 11)

Compound 21 (1 equivalent, 11 μmol) was dissolved in water (150 μL), and 5′-azido-5′-deoxyguanosine (1 equivalent, 11 μmol) was dissolved in DMSO (50 μL), followed by mixing both the solutions by stirring for 10 min. Next, aqueous solutions of CuSO_4_ (0.3 equivalents, 3.3 μmol, 10 μL) and sodium ascorbate (10 equiv., 110 μmol) were added. The reaction mixture was then stirred at 25 °C for 1 h. The reaction mixture was quenched by adding 500 μL of an aqueous solution of Na_2_EDTA (1 equiv., 11 μmol). The final product was purified by ion-exchange chromatography using a DEAE Sephadex™ A-25 column (HCO_3_^−^ form) and RP-HPLC with a semi-preparative column (column A and method D) (Table S1†). Compound 11 was isolated as an ammonium salt in 62% yield.

^1^H NMR: *δ*_H_ (500.24 MHz; D_2_O; TSP) 9.11 (1H, s, H8 m^7^G), 8.42 (1H, s, H8), 8.26 (1H, s, H8), 7.93 (1H, s, H2), 7.55 (1H, s, Htriazol), 6.06 (1H, d, *J* = 5.2, H1′), 5.89 (1H, d, *J* = 3.4, H1′), 5.76 (1H, d, *J* = 2.8, H1′), 4.85 (2H, dd, *J* = 6.2, 2.8), 4.76, 4.72–4.69 (1H, m, H2′), 4.62–4.57 (2H, m, H2′ and H3′), 4.52 (1H, t, *J* = 5.3, 5.1, H4′), 4.46–4.43 (4H, m), 4.41 (1H, dd, *J* = 4.1, 2.6), 4.38–4.33 (2H, m), 4.30–4.23 (3H, m), 4.02 (3H, s, CH_3_ m^7^G), and 3.25 (3H, s, CH_3_ 2′-OMe). ^31^P NMR: *δ*_P_ (161.89 MHz; D_2_O; H_3_PO_4_) −0.74 (1P, s, ApG), −7.94 (d, *J* = 22.2), −11.49 (d, *J* = 19.1), and −23.32 (1P, t, *J* = 20.6, Pβ). HRMS (ESI−) calculated for [C_35_H_45_N_18_O_24_P_4_^−^] 1145.2147; *m*/*z* found: 1145.2154.

### Biological and biochemical experiments

#### General procedure (C) of synthesis and purification of short RNAs and determination of capping efficiency

Short RNAs were generated as described by Sikorski *et al.*^7^ with modifications. Annealed oligonucleotides (CAGTAATACGACTCACTATAGGGGAAGCGGGCATGCGGCCAGCCATAGCCGATCA and TGATCGGCTATGGCTGGCCGCATGCCCGCTTCCCCTATAGTGAGTCGTATTACTG) containing T7 promoter sequence and recognition site for DNAzyme were used as templates for *in vitro* transcription reactions. Reactions were set in 50 μL mixtures which contained RNA Pol buffer (40 mM Tris–HCl pH 7.9, 10 mM MgCl_2_, 1 mM DTT, 2 mM spermidine); 1 μM annealed oligonucleotides; ATP, CTP, UTP, 2 mM each and 0.5 mM GTP (Thermo Fisher Scientific); 3 mM cap analog of interest; 1 U μL^−1^ RiboLock RNase Inhibitor (Thermo Fisher Scientific), 5 μL of home-made T7 RNA polymerase, and were incubated at 37 °C; after 1.5 h additional 5 μL of home-made T7 RNA polymerase were added and reactions were conducted for another 1.5 h at 37 °C; next, DNase I (EN0521, Thermo Fisher Scientific) was added (0.1 U μL^−1^), followed by incubation for 30 min incubation at 37 °C. A control sample – non-capped short RNA – was prepared in reaction with ATP, CTP, UTP, and GTP, 2 mM each, and no cap (analog) added. Transcripts were purified using the RNA Clean & Concentrator™-25 kit (Zymo Research). RNAs of the intended size for further processing and analysis were recovered from the fractions collected between 18 and 21 min of the HPLC purification program. For purification Phenomenex Clarity® 3 μm Oligo-RP column was utilized and a linear gradient of buffer B (0.1 M triethylammonium acetate pH 7.0 and 50% acetonitrile) from 10–26.7% in buffer A (0.1 M triethylammonium acetate pH 7.0) over 25 min at 1 mL min^−1^ was applied. Recovered by isopropanol precipitation, RNAs, to generate in these homogenous 3′-ends, were incubated in 1 μM concentration with 1 μM DNAzyme 10–23 in 50 mM MgCl_2_ and 50 mM Tris–HCl (pH 8.0) at 37 °C for 1 h. Obtained due to that 3′-homogenous ∼25-nt RNAs were purified using RNA Clean & Concentrator™-25 kit (Zymo Research) and treated with DNase I to remove DNAzyme. Samples were analyzed in 15% acrylamide/7 M urea/TBE gel. The capping efficiency was examined based on densitometric quantification of the bands. The intensities of the bands of uncapped (RNA_25_) and capped RNA (RNA_26_, RNA_27_, and RNA_28_ for di-, tri-, and tetranucleotide-capped transcripts, respectively) were used to calculate the percentage of capped RNA *i.e.*, capping efficiency in the analyzed samples. The values were determined by calculating the capped fraction percentage of the sum of the intensities of both bands (corresponding to capped and uncapped RNAs). The pppG-RNA sample served as a marker for uncapped transcripts, with the most intense band marking for 25-nt RNA.

#### Plasmid linearization (template for *in vitro* transcription)

Plasmid pJET_T7_Gluc_128A, encoding *Gaussia* luciferase, was linearized with AarI restriction enzyme (Thermo Fisher Scientific), overnight at 37 °C. The template was purified using the QIAquick™ PCR Purification kit. Linearized plasmid was used as a template for *in vitro* transcription reactions.

#### mRNA synthesis and purification

mRNAs encoding *Gaussia* luciferase were obtained as previously described by Sikorski *et al.* with modifications. Reactions were set in 20 μL mixtures which contained: RNA polymerase buffer (pH 7.9; 40 mM Tris–HCl, 10 mM MgCl_2_, 1 mM DTT, and 2 mM spermidine); 45 ng μL^−1^ DNA template; 2 mM ATP, CTP, and UTP each and 0.5 mM GTP (Thermo Fisher Scientific); 3 mM cap analog of interest; 1 U μL^−1^ RiboLock RNase Inhibitor (Thermo Fisher Scientific), 2 μL of home-made T7 RNA polymerase. Reaction mixture was incubated for 2 h at 37 °C. Next, additional 2 μL of home-made T7 RNA polymerase was added, and reaction mixture was incubated for another 2 h at 37 °C. Then, 0.1 U μL^−1^ DNase I (Thermo Fisher Scientific) was added, followed by incubation for 30 min at 37 °C. A control sample – non-capped mRNA – was prepared in reaction with 2 mM ATP, CTP, UTP, and GTP each, and no cap analog was added. Crude mRNAs were purified using NucleoSpin RNA Clean-up XS (Macherey-Nagel). For cell studies, non-capped mRNA from samples with capped mRNAs were removed by treatment with RNA 5′-polyphosphatase (RP8092H; Epicentre) and Xrn1 (New England Biolabs), two separate treatments, with purification using the NucleoSpin RNA Clean-up XS step in between. After enzymatic reactions, transcripts were again purified using NucleoSpin RNA Clean-up XS. RNA was purified by HPLC using RNASep™ Prep – RNA Purification Column (ADS Biotec) at 55 °C, a linear gradient of buffer B (0.1 M triethylammonium acetate pH 7.0 and 50% acetonitrile) from 17.5–25.8% in buffer A (0.1 M triethylammonium acetate pH 7.0) over 20 min at 0.9 mL min^−1^ was applied. mRNAs from the collected fractions were recovered by isopropanol precipitation. Non- and HPLC-purified transcripts were analyzed in 1× TBE agarose gel (1.2%), with RiboRuler High Range RNA Ladder (Thermo Fisher Scientific) (Fig. S1†).

#### *In vitro* translation studies in RRL system

The cap analogs were co-transcriptionally incorporated into the 5′ end of RNA encoding *Gaussia* luciferase. The standard IVT reaction contained 2 mM ATP, UTP, CTP, 0.5 mM of GTP and 3 mM of cap analog (6-fold excess over GTP), and a dsDNA template. The IVT reactions were performed for 4 h at 37 °C in the presence of T7 polymerase. The samples were treated with DNase and purified using a commercial kit – NucleoSpin RNA Clean-up XS (Macherey-Nagel). Obtained transcripts were analyzed in 1× TBE agarose gel (1.2%) (Fig. S1†). These mRNAs were used to program RRL to determine *in vitro* translation efficiency.

The translation efficiency of non-HPLC purified mRNA encoding *Gaussia* luciferase was determined using an RRL system (Promega). A typical *in vitro* translation reaction (10 μL) contained 4 μL of RRL mixture, 0.05 μL of amino acid mixture excluding leucine, 0.05 μL of amino acid mixture excluding methionine, 1.9 μL of 1 M potassium acetate, 0.4 μL of 25 mM magnesium chloride, 0.2 μL of Ribolock RNase Inhibitor (Thermo Fisher Scientific), 2.4 μL of water, and 1 μL of one of tested mRNA. Four stock solutions of capped mRNAs were prepared. For mRNAs capped with ARCA, m^7^GpppG, GpppG, m^7^GpppApG, 1, 2, 5, 6, 7, 8, and 10B, solutions of concentrations 93.75, 70.31, 46.88, and 23.44 pg μL^−1^ were used. For mRNAs capped with m^7^GpppA_m_pG, 3, 4, and 9, 280, 140, 93.75, and 70.31 pg μL^−1^ stocks were used. The translation reactions were pre-incubated for 1 h at 30 °C. Following incubation, the reaction mixtures were cooled on ice, and 1 μL of tested mRNAs encoding *Gaussia* luciferase from the stocks was added, followed by incubation for 1 h at 30 °C. The translation reaction was stopped by freezing the sample in liquid nitrogen.

#### Determination of translation efficiency in RRL system

The activity of the synthetized *Gaussia* luciferase produced by *in vitro* translation (translation efficiency) was determined by measuring luminescence using a Synergy H1 Microplate Reader (BioTek). The samples were defrosted and transferred to white non-binding 96-well plates. The luminescence of each sample was measured in the presence of 50 μL h-coelenterazine (10 ng mL^−1^; NanoLight) in PBS. Luciferase activity was plotted as a function of mRNA concentration to determine initial translation efficiency (initial slope, Fig. S2†). The translation efficiency was calculated using the estimated linear regression coefficient. All results were normalized to the data obtained for ARCA-capped RNA. GpppG-RNA (non-functional) was used as a negative control.

#### Protein production in JAWS II cells

JAWS II (ATCC: CRL-11904) murine immature dendritic cell line was used for translation studies as described by Sikorski *et al.*^7^ Cells were cultured in RPMI 1640 medium supplemented with 10% fetal bovine serum, sodium pyruvate, penicillin/streptomycin, and GM-CSF (5 ng mL^−1^; 315-03; PeproTech) at 5% CO_2_ and 37 °C. On the day of the experiment, 104 cells were seeded in 100 μL medium/well in a 96-well plate. Cells in each well were transfected using a mixture containing HPLC-purified mRNA (25 ng) in Opti-MEM (5 μL; 51985026; Gibco) mixed with Lipofectamine MessengerMAX Transfection Reagent (0.3 μL; Invitrogen) in additional Opti-MEM (5 μL). For each mRNA sample, three wells were used as technical replicates. Sixteen hours after transfection, the medium was collected and replaced with fresh medium (100 μL). The medium collection and replacement were conducted three more times at the indicated time points. To detect *Gaussia* luciferase activity, 50 μL of 10 ng mL^−1^ h-coelenterazine (301, NanoLight) in PBS was added to 10 μL of cell culture medium, and luminescence was measured on a Synergy H1 Microplate Reader (BioTek). Total protein production for each mRNA over 4 days (cumulative luminescence) was reported as the mean value ± SEM normalized to ARCA-capped RNA. Transcripts capped with m^7^GpppA_m_pG (cap1) and ARCA were used as positive control, and uncapped (5′-triphosphate) mRNA and mock (no mRNA) were used as negative controls. The experiments were performed in duplicate using mRNAs from two independent synthesis sets.

#### Dot blot analysis

mRNA (25 ng) was analyzed as previously described by Sikorski *et al.* with modifications.^7^ Transcripts were blotted onto Amersham™ Hybond™-N+ Membrane (RPN203B; GE Healthcare) and UV cross-linked. Membranes were blocked with 5% skim dried milk in PBST buffer with 0.1% Tween-20, followed by incubation with dsRNA-recognizing mouse monoclonal J2 antibody (SCICONS). Goat anti-mouse IgG (H+L) Poly-HRP Secondary Antibody (32230; Invitrogen) and Immobilon Western Chemiluminescent HRP Substrate (WBKLS0100; Merck Millipore) were used for signal detection using the Amersham Imager 600 (GE Healthcare).

#### Determination of association constant (*K*_AS_) for eIF4E

The dissociation constants for eIF4E were determined by fluorescent quenching titration (FQT) experiments. The 0.1 μM solution of eIF4E (1400 μL) in 50 mM HEPES/KOH buffer (pH 7.20) containing 100 mM KCl, 0.5 mM EDTA, and 1 mM DTT was incubated for 45 min at 20 °C. After incubation, aliquots (1 μM) of the tested cap analogs (m^7^GpppA_m_pG, 2, 4, 6, 8, 9, 10b, and 11) were added, and fluorescence measurements were conducted. The samples were excited at 280 nm (5 nm bandwidth) and detected at 337 nm (10 nm bandwidth). The association constants (*K*_AS,_ μM^−1^) were determined by fitting a previously described equation to the obtained data points.^31^ For each cap analog, FQT experiments were performed in triplicate. *K*_D_ values (*K*_AS_^−1^) are presented as weighted averages.

#### RNA decapping assay

Short 27-nt capped RNAs were synthesized according to the general procedure (C). Short 27-nt RNA transcripts (20 ng) capped with m^7^GpppA_m_pG (cap1), 4, or 9 were incubated with 11 nM hDCP1/DCP2 complex in 50 mM Tris/HCl (pH 8), 50 mM NH_4_Cl, 0.01% Igepal, 1 mM DTT, 5 mM MgCl_2_, and 2 mM MnCl_2_ at 37 °C for the indicated durations. Reactions were terminated by adding equal volume of loading dye (4.5 M urea, 50% formamide, 20 mM EDTA, 0.03% bromophenol blue, and 0.03% xylene cyanol) and flash freezing. Then, samples were resolved by PAGE using denaturing 15% acrylamide/7 M urea/TBE gel. Bands were stained with SYBR Gold (Invitrogen) and visualized using a Typhoon FLA 9500 (GE Healthcare) (Fig. S2†). Band intensities corresponding to capped and uncapped RNAs were quantified densitometrically using 1D Gel Image Analysis Software TotalLab CLIQS.

## Author contributions

M. K., J. K. and J. J. designed the study, M. K. performed chemical syntheses, RRL experiments and fluorescence quenching titration experiments, K. D. performed experiments in cells, M. B. performed decapping assays, M. W. provided chemical reagents. M. K., K. D., J. K. and wrote the first draft of the manuscript. The manuscript was written through contributions of all authors. All authors have given approval to the final version of the manuscript.

## Conflicts of interest

There are no conflicts to declare.

## Supplementary Material

RA-013-D3RA00026E-s001
