# Supplementary material for: Towards superior mRNA caps accessible by click chemistry: synthesis and translational properties of triazole-bearing oligonucleotide cap analogs
Source: RSC Adv. 2023 Apr 25;13(19):12809–24. doi: 10.1039/d3ra00026e (PMC10126820; doi:10.1039/d3ra00026e)
Supplement: RA-013-D3RA00026E-s001 [file RA-013-D3RA00026E-s001.pdf]

Electronic Supplementary Material (ESI) for RSC Advances.
This journal is © The Royal Society of Chemistry 2023

Towards superior mRNA caps accessible by click chemistry: synthesis and translational properties of triazole-bearing oligonucleotide cap analogs

Mateusz Kozarski [a, b], Karolina Drazkowska[b], Marcelina Bednarczyk [a, b], Marcin Warminski [a], Jacek Jemielity [b*], Joanna Kowalska [a*]

[a]Division of Biophysics, Institute of Experimental Physics, Faculty of Physics, University of Warsaw, Pasteura 5, 02-093 Warsaw, Poland

[b]Centre of New Technologies, University of Warsaw, Banacha 2c, 02-097 Warsaw, Poland

List of contents

MATERIALS AND METHODS **2**

TABLES **2**

TABLE S1. HPLC METHODS USED WITH AGILENT TECHNOLOGIES SERIES 1200 OR SHIMADZU RF-20A 2
TABLE S2. THE AFFINITY OF CAP ANALOGS FOR EIF4E AT 20 °C DETERMINED USING FQT EXPERIMENTS. 3
TABLES S3. NMR SPECTRA, HR MS, HPLC PROFILES OF SYNTHETIZED MODIFIED NUCLEOTIDES AND CAP ANALOGUES. 3
5′-N_3APG 12A 3
5′-N_3A_MPG 12B 7
5′-$N_3C_2H_4PA_M$PG 12C 10
M7GPPP-TR-APG 1 12
M7GPPP-TR-A_MPG 2 15
M7GPPP-CH_2-TR-APG 3 17
M7GPPP-CH_2-TR-A_MPG 4 20
M7GPPP-C_2H_4-APG 5 22
M7GPPP-C_2H_4-A_MPG 6 25
M7GPPPOCH_2-TR-APG 7 28
M7GPPPOCH_2-TR-A_MPG 8 31
M7GPPP-TR-$C_2H_4PA_M$PG 9 34
M7GPPPG-CARB-CH_2-TR-APG (BOTH ISOMERS) 10A AND 10B 37
2′-OME-3′-OC_3H_3-ADENOSINE 42
5′-MONOPHOSHAPTE 2′-OME-3′-OC_3H_3 ADENOSINE (2′-OME-3′-OC_3H_3AMP) 44
M7GPPPA-2′OME-3′-OC_3H_3 47
M7GPPPA_M-3′-OCH_2-TR-G 11 50

FIGURES **53**

FIGURE S1. MRNA PURITY 53
FIGURE S2. LUCIFERASE ACTIVITY IN RABBIT RETICULOCYTE LYSATE 55
FIGURE S3. SUSCEPTIBILITY TO DEGRADATION BY HDCP1/DCP2 COMPLEX. 55

Materials and methods

Solvents, chemical reagents, and starting materials were purchased from commercial sources. Phosphoramidites were purchased from ChemGenes (2'-O-PivOM-5'-DMT-A (N-PAC) 3'-CEP) and Link Technologies (2'-OMe-Bz-A-CE). Solid support for solid phase synthesis was purchased from GE Healthcare (Primer SupportTM 5G ribo G 300 (309 µmol ribo G/g). C- phosphonate nucleotide analogues were obtained according to the previously described procedures (Walczak et al.). [1]2-Cyanoethyl 2-bromoethyl N, N-diisopropylamino Phosphite was synthesized as previously described (Hebert et al.). [2] The analytical analysis and purifications were performed using Agilent Technologies Series 1200 with UV detection at 254 nm wavelength and fluorescent detection (ex, 260 nm; em. 370 nm) and Shimadzu RF-20A. For chemical reaction analysis the Gemini 3 µm NX-C18 110Å 150 x 4.6 mm column was used with linear gradient of methanol in 0.05 M ammonium buffer, pH 5.9. For purification the Gemini 5 µm NX-C18 110 Å 150 x 10 mm column (column A) or HiChrom Vydac Denali 5 µm C18 120 Å 150 x 10 mm (column B) or Dr. Maisch VisionHT C18 Highload 25 cm × 2 cm, 10 µm (column C) were used with linear gradient of acetonitrile in 0.05 M ammonium buffer, pH 5.9. Additionally, for purification, ion-exchange chromatography on DEAD Sephadex resin A-25 (HCO_3^- form) was used. To confirm the purity and structure of intermediate and final products high resolution mass spectrometry (HRMS) and nuclear magnetic resonance (NMR) were used. Mass spectra was recorded using Thermo Scientific LTQ Orbitrap Velos spectrometer, with the negative electrospray ionization HRMS (-) ESI. NMR spectra were recorded using a Bruker AVANCE III HD 500 MHz spectrometer equipped with a high-stability temperature unit with 5 mm PAB- BOBB/19F-1H/D Z-GRD Z119470/0188 probe at 500.24MHz (^{1}H NMR) and 202.49 MHz (31P NMR) or Varian 400 MHz with OneNMR_W016 probe at 399.90 MHz (1H NMR) and 161.89 MHz (^{31}P NMR). The 1H NMR and ^{31}P NMR chemical shifts (δH and δP) were reported in ppm and referenced to the respective standards: sodium 3-(trimethylsilyl)-2,2,3,3 tetradeuteropropionate (TSP, internal standard) and 20% phosphoric acid in D_2O (external standard).

Tables

Table S1. HPLC methods used with Agilent Technologies Series 1200 or Shimadzu RF-20A

**Analytical methods**			
**Method**	**Buffer A**	**Buffer B**	**Gradient**
**A**	0.05 M ammonium buffer, pH 5.9	0.05 M ammonium buffer, pH 5.9/MeOH (v/v, 1/1)	Linear gradient 0%- 50% of buffer B; 15 min; 1ml/min
**B**	0.05 M ammonium buffer, pH 5.9	0.05 M ammonium buffer, pH 5.9/MeOH (v/v, 1/1)	Linear gradient 0%- 100% of buffer B; 15 min; 1ml/min
**C**	0.05 M ammonium	0.05 M ammonium buffer,	Linear gradient 0%- 50% of buffer B; 7.5 min; 1ml/min

	buffer, pH 5.9	pH 5.9/MeOH (v/v, 1/1)	
**Preparative methods**			
**D**	0.05 M ammonium buffer, pH 5.9	0.05 M ammonium buffer, pH 5.9/Acetonitryle (30%)	Linear gradient 0%- 50% of buffer B; 60 min; 4.7 ml/min

Table S2. The affinity of cap analogs for eIF4E at 20 °C determined using FQT experiments.

The experiments were performed in triplicate. The values in table are presented as weighted averages.

Cap No.	K_{AS} (μM^{-1})
m^7GpppA_mpG	29.6 ± 2.3
**2**	23.8 ± 3.6
**4**	29.7 ± 6.0
**6**	30.7 ± 6.9
**8**	20.3 ± 3.4
**9**	136.1 ± 13.2
**10b**	35.3 ± 8.0

Tables S3. NMR spectra, HR MS, HPLC profiles of synthetized modified nucleotides and cap analogues.

***5'*-N$_3$ApG 12a**

Structure	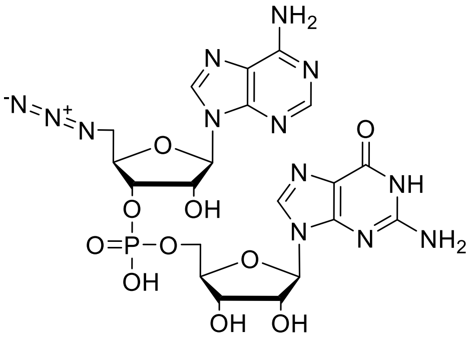

HPLC Profile

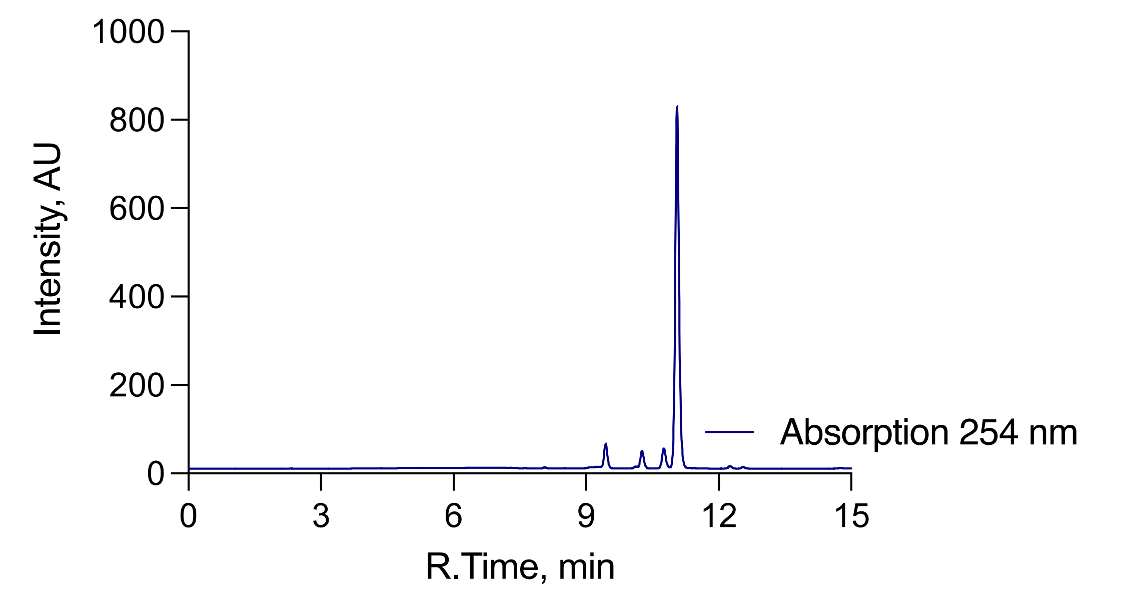

HR MS

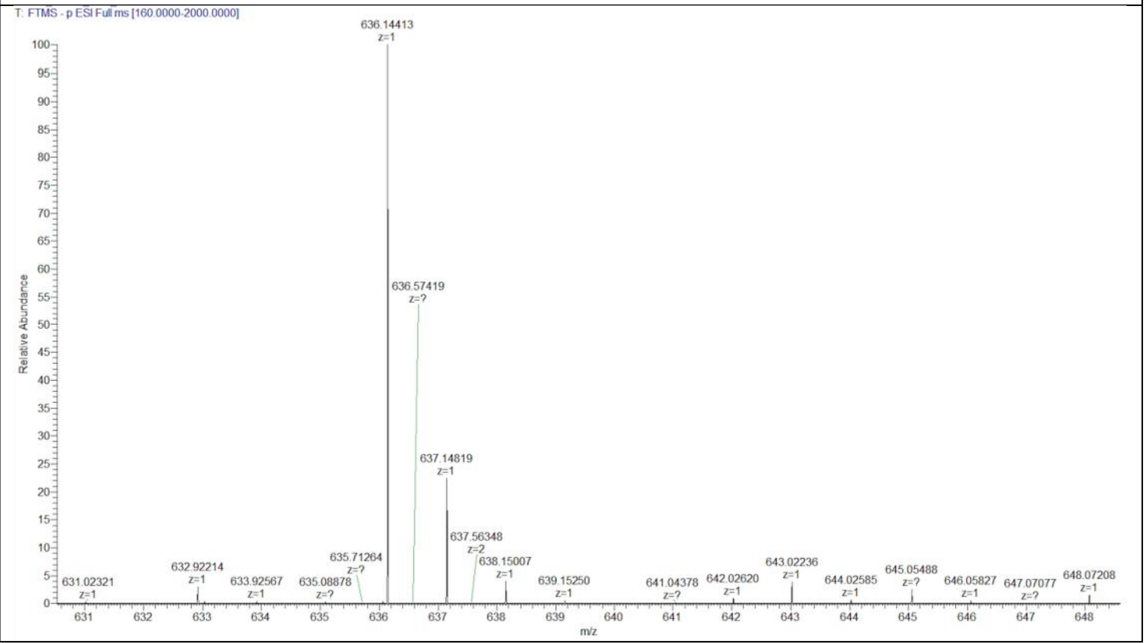

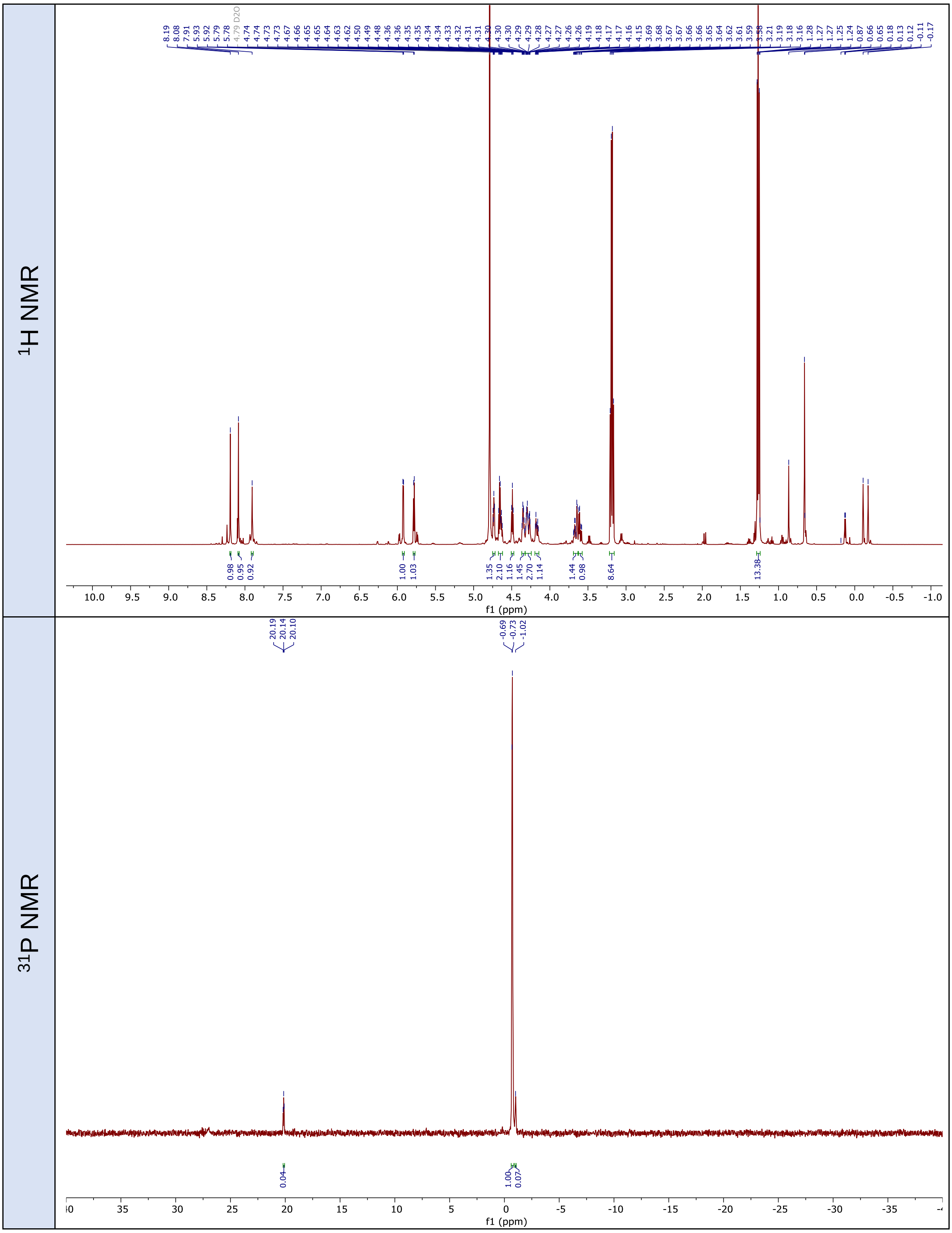

1H NMR
8.19
8.08
7.91
5.93
5.92
5.79
5.78
4.79 D2O
4.74
4.74
4.73
4.73
4.67
4.66
4.65
4.65
4.64
4.63
4.62
4.50
4.49
4.48
4.36
4.36
4.35
4.35
4.34
4.34
4.33
4.32
4.31
4.31
4.30
4.30
4.30
4.29
4.28
4.27
4.27
4.26
4.26
4.19
4.18
4.17
4.17
4.16
4.15
3.69
3.68
3.67
3.67
3.66
3.66
3.65
3.64
3.62
3.61
3.59
3.58
3.21
3.19
3.18
3.16
1.28
1.27
1.27
1.25
1.24
0.87
0.66
0.65
0.18
0.13
0.12
-0.11
-0.17
0.98
0.95
0.92
1.00
1.03
1.35
2.10
1.16
1.45
2.70
1.14
1.44
0.98
8.64
13.38
f1 (ppm)
31P NMR
20.19
20.14
20.10
-0.69
-0.73
-1.02
0.04
1.00
0.07
f1 (ppm)

^{1}H-^{1}H COSY NMR

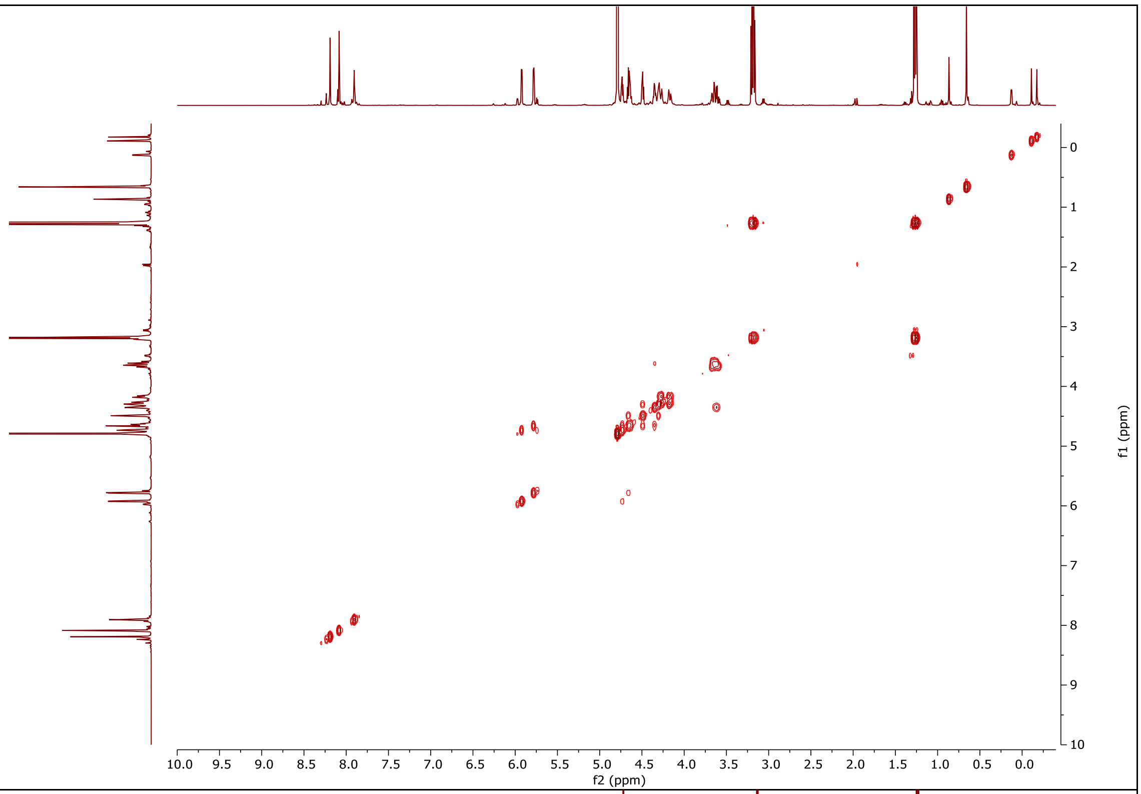

^{1}H-^{13}C HSQC NMR

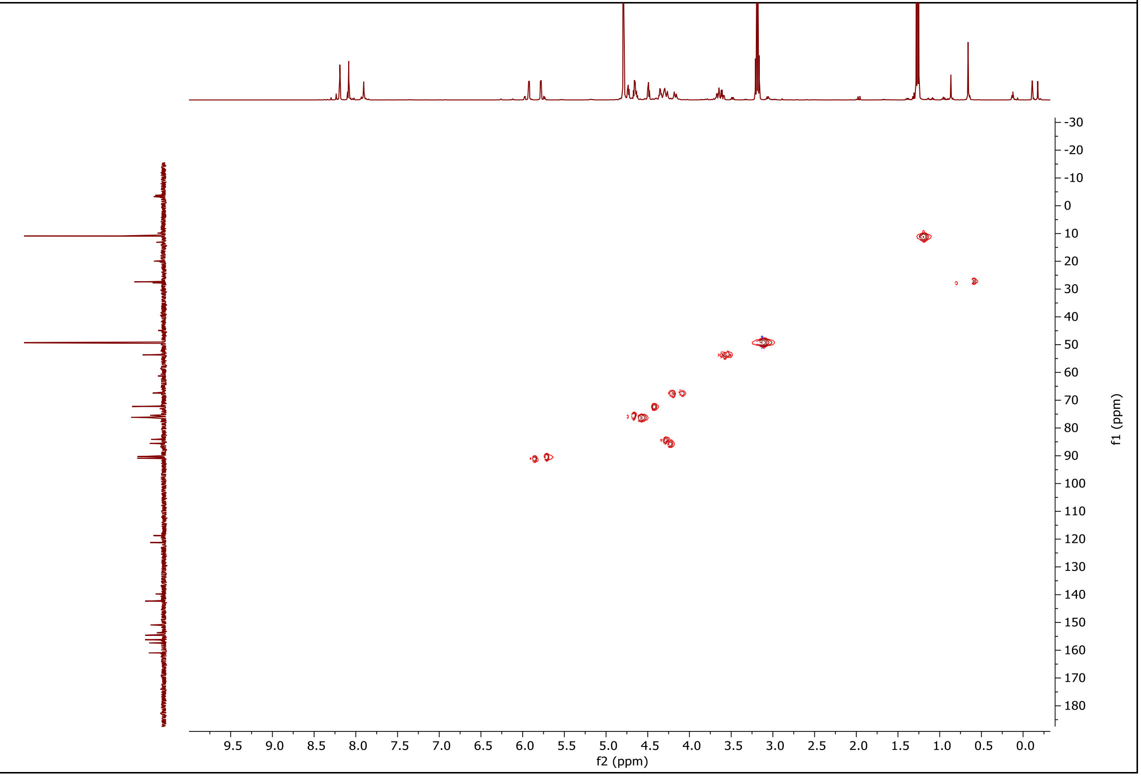

5'-N$_3$A$_m$pG 12b

Structure

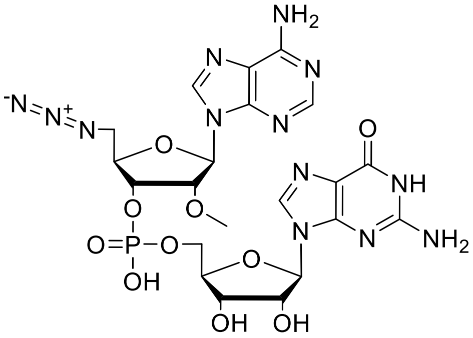

HPLC Profile

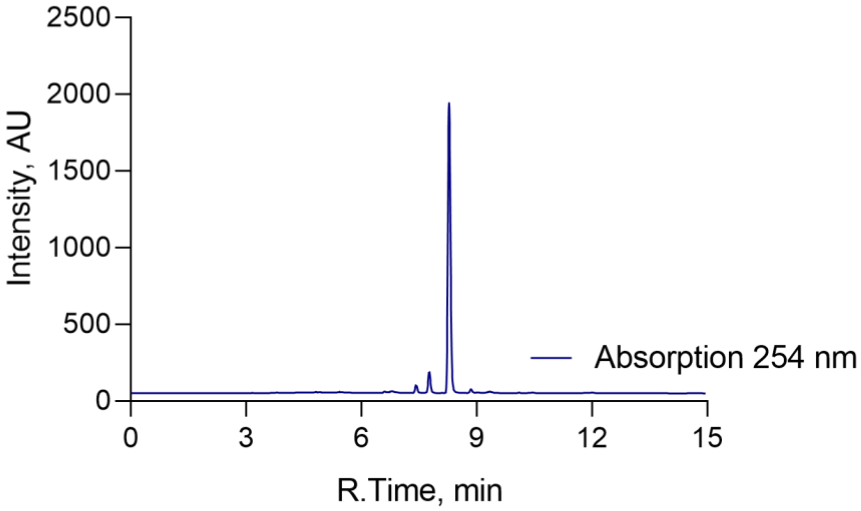

HR MS

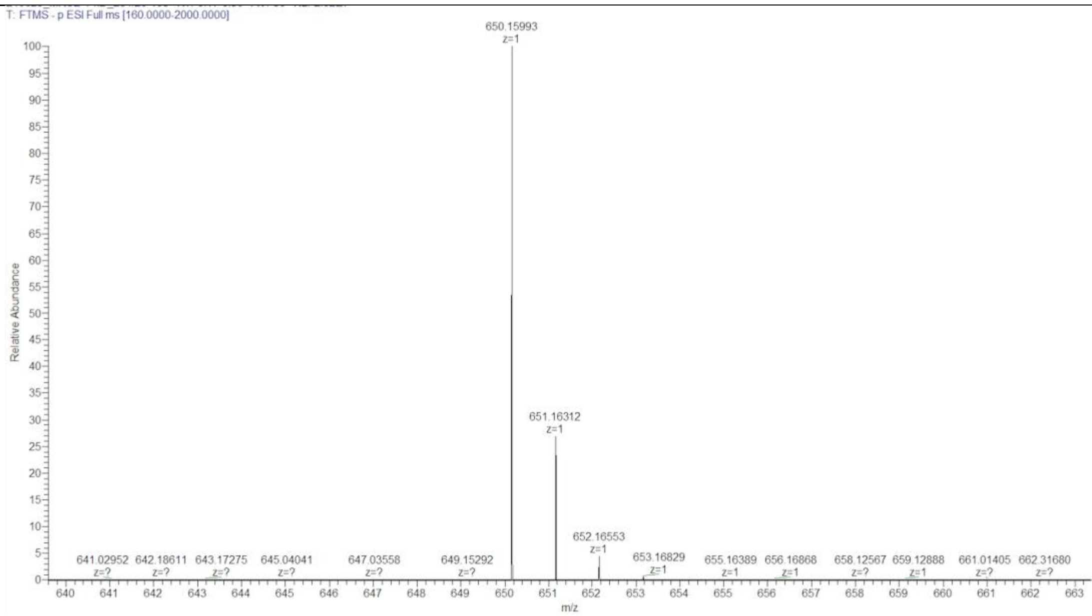

^{1}H NMR

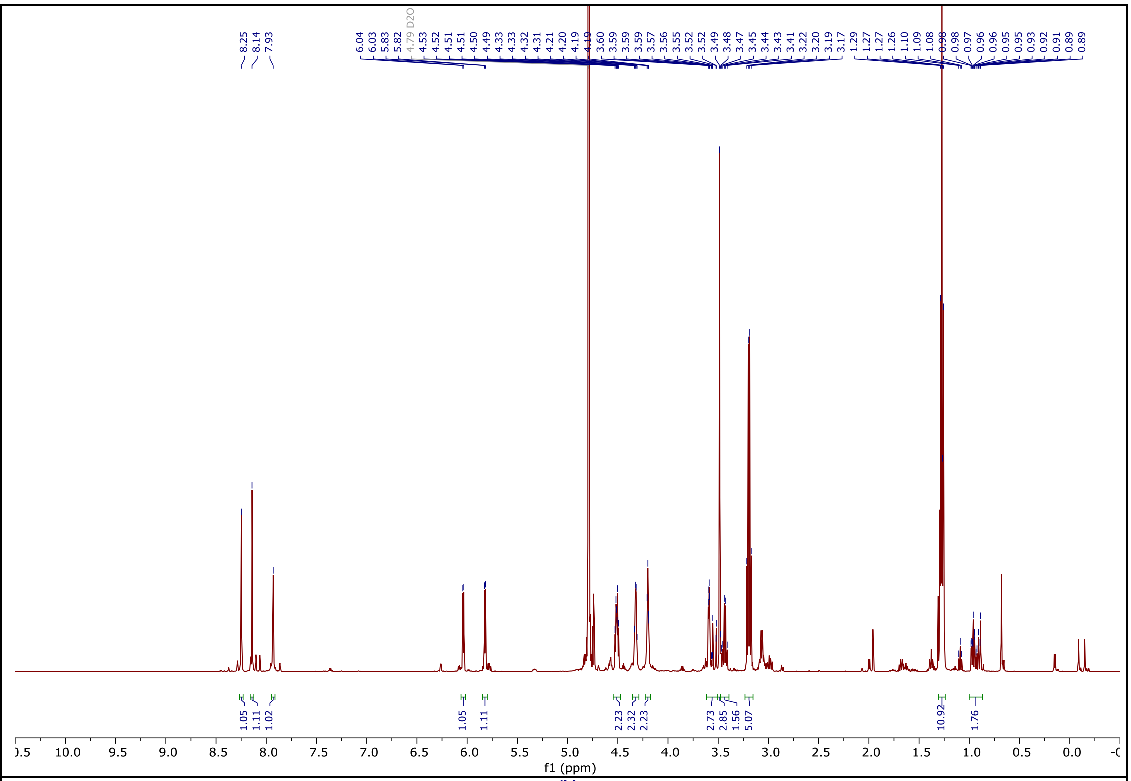

^{31}P NMR

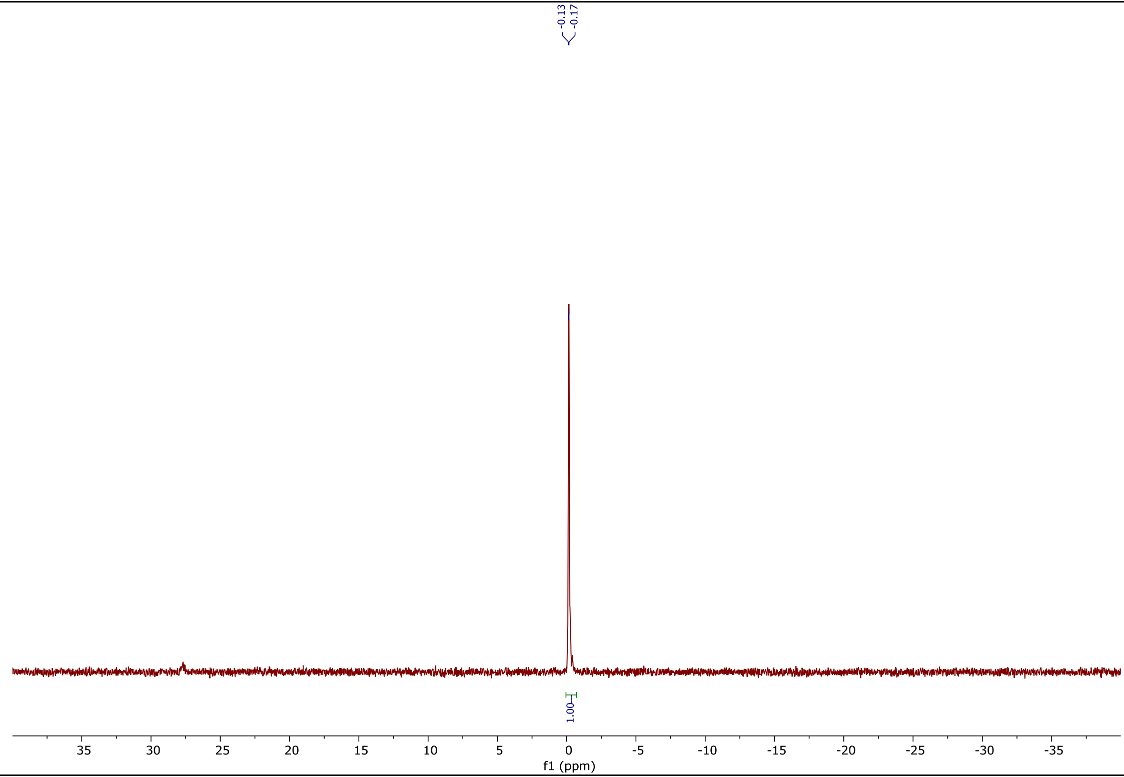

^1H-^1H COSY NMR

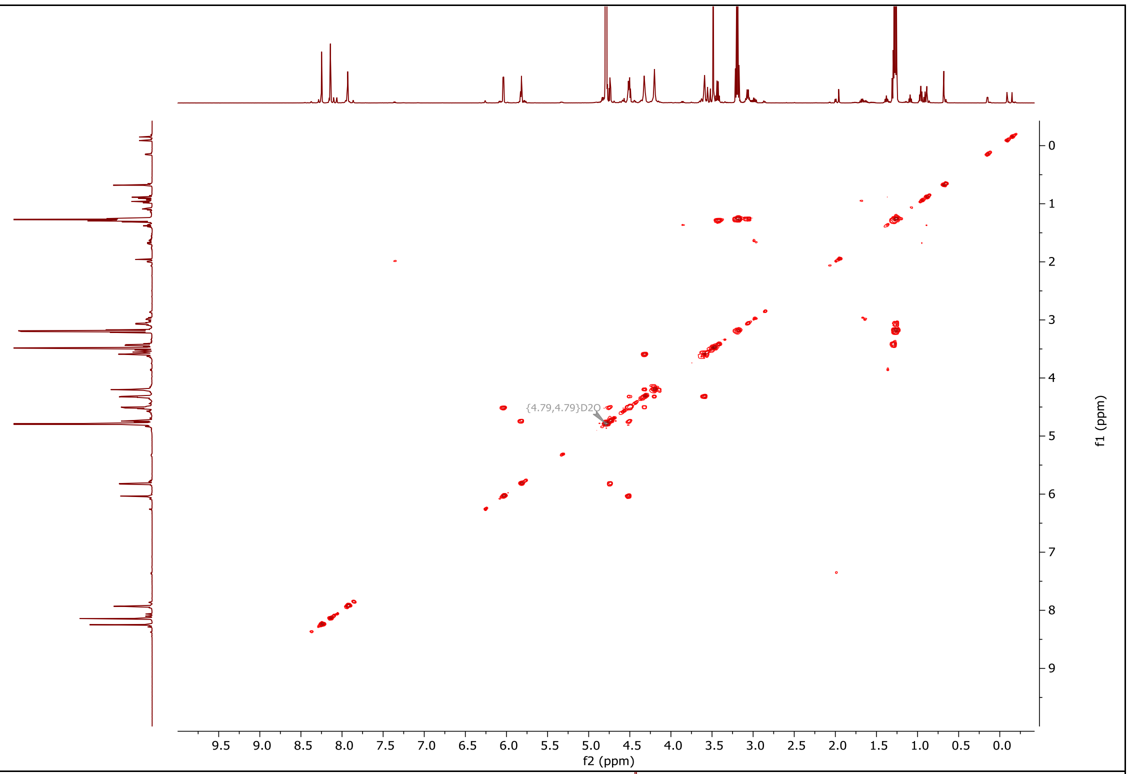

^1H-^{13}C HSQC NMR

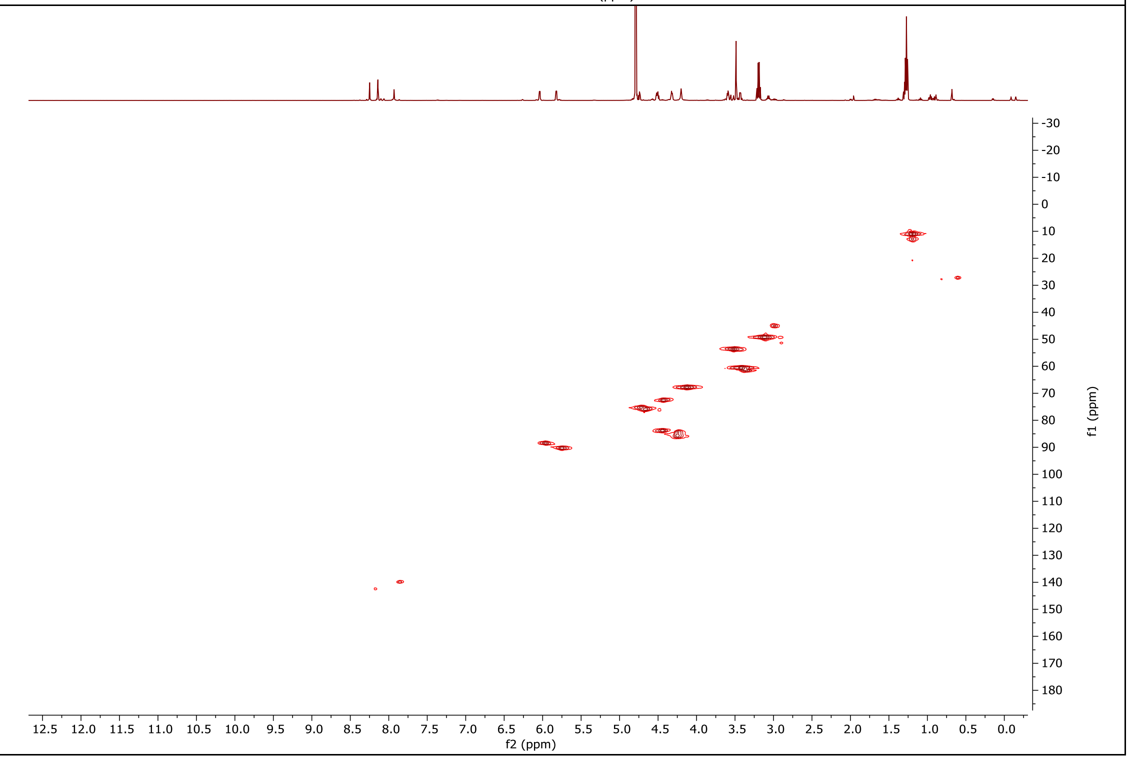

5’-$N_3C_2H_4$pA$_m$pG 12c

Structure

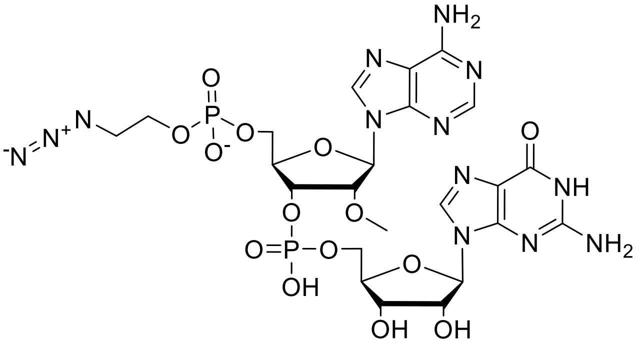

HPLC Profile

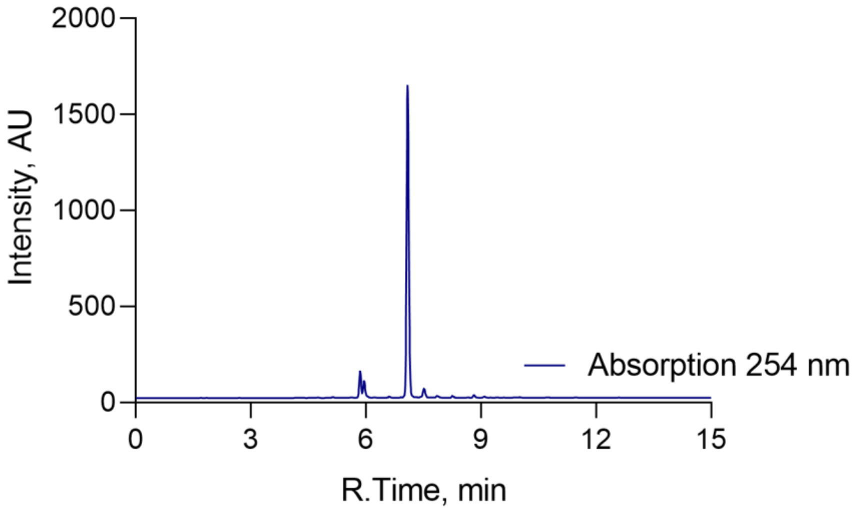

HR MS

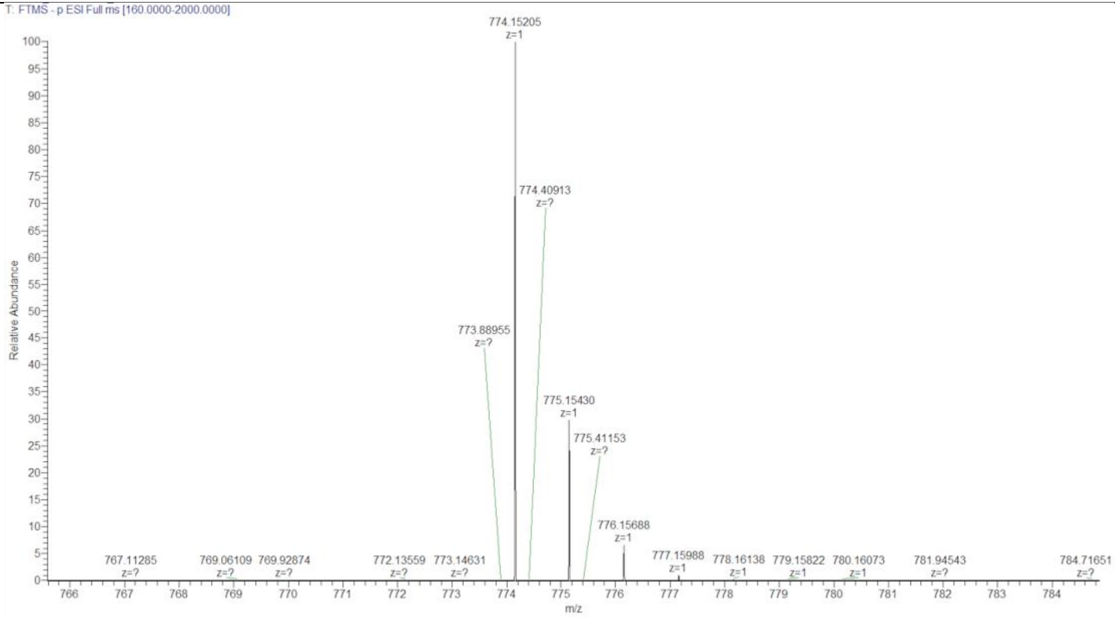

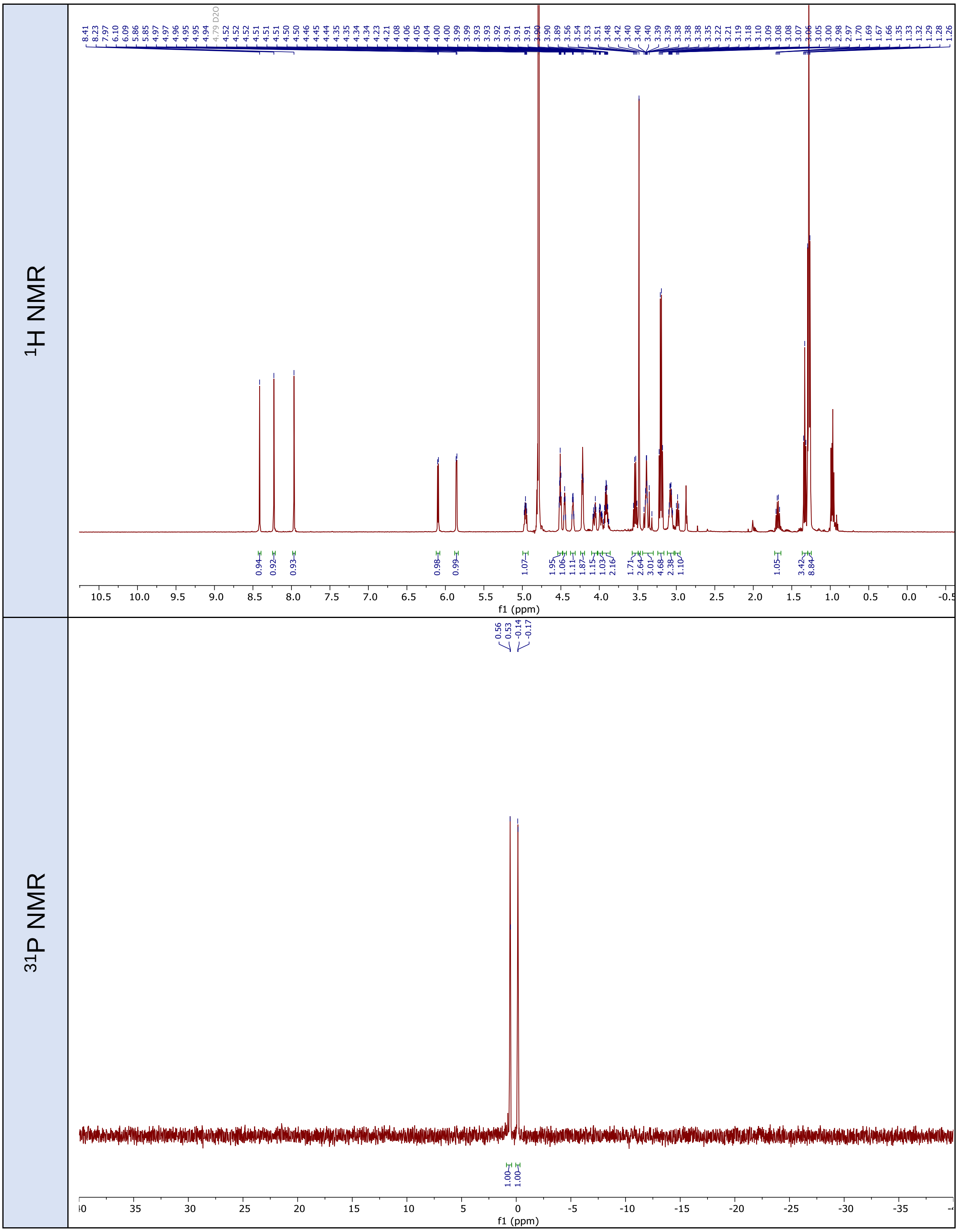

1H NMR
f1 (ppm)
31P NMR
f1 (ppm)

^{1}H-^{1}H COSY NMR

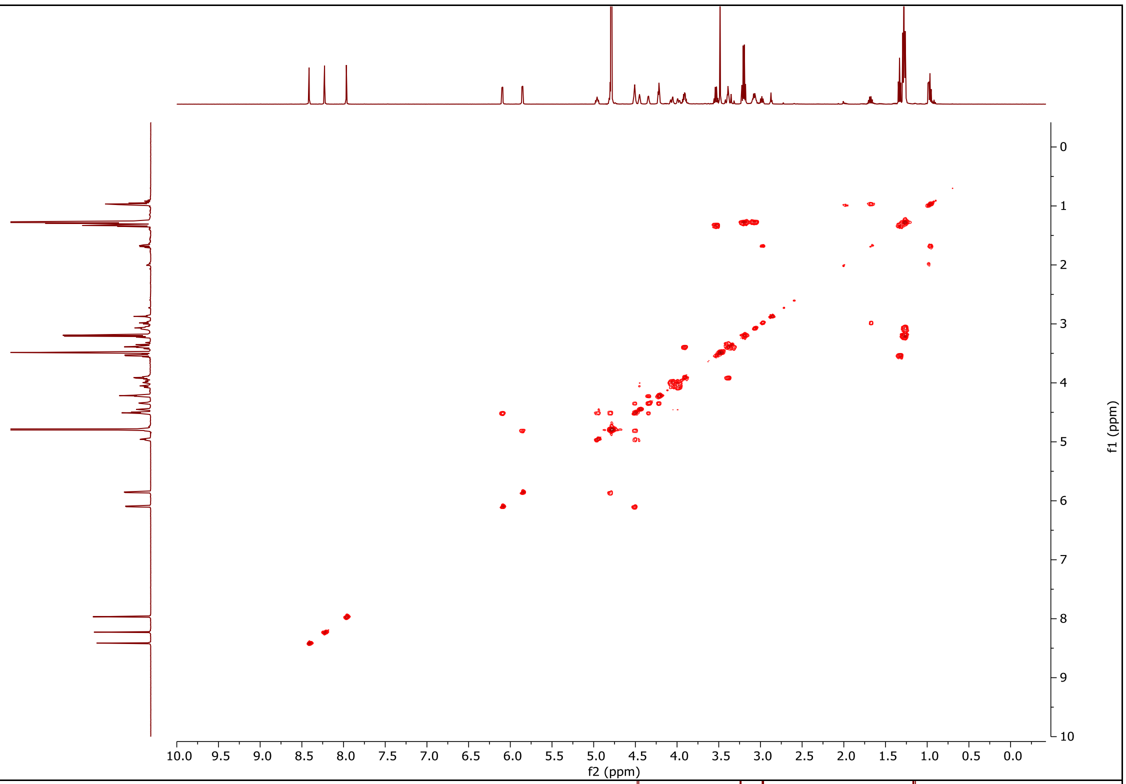

^{1}H-^{13}C HSQC NMR

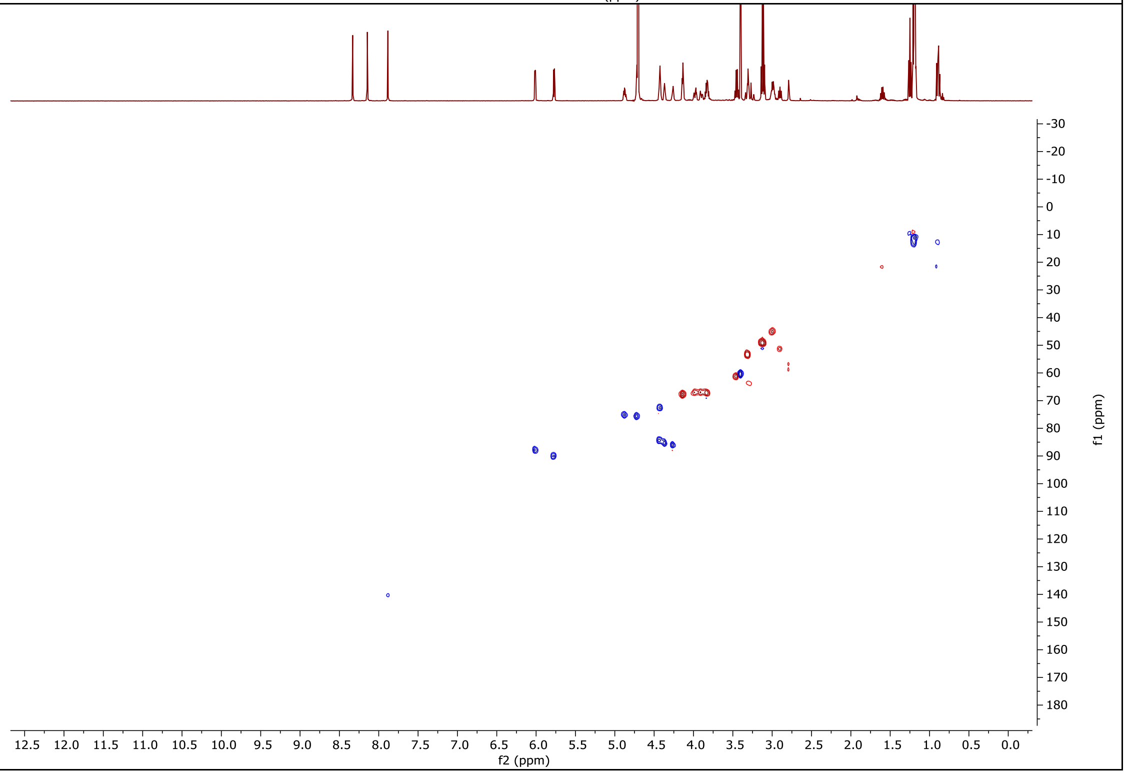

m^{7}Gppp-tr-ApG 1

Structure

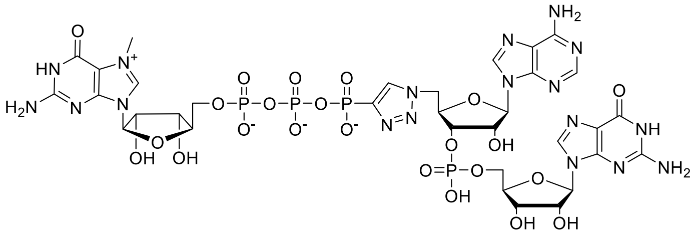

HPLC Profile

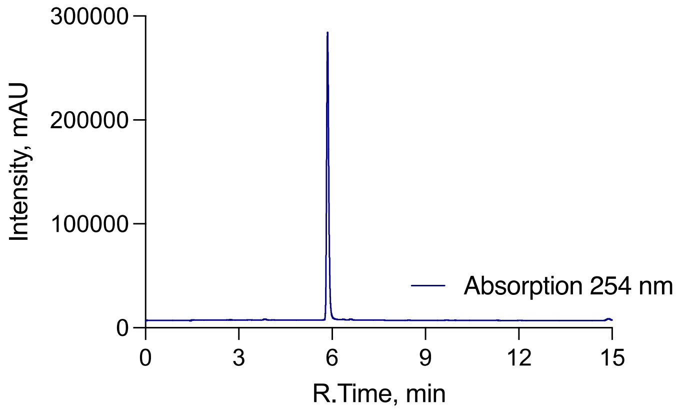

HR MS

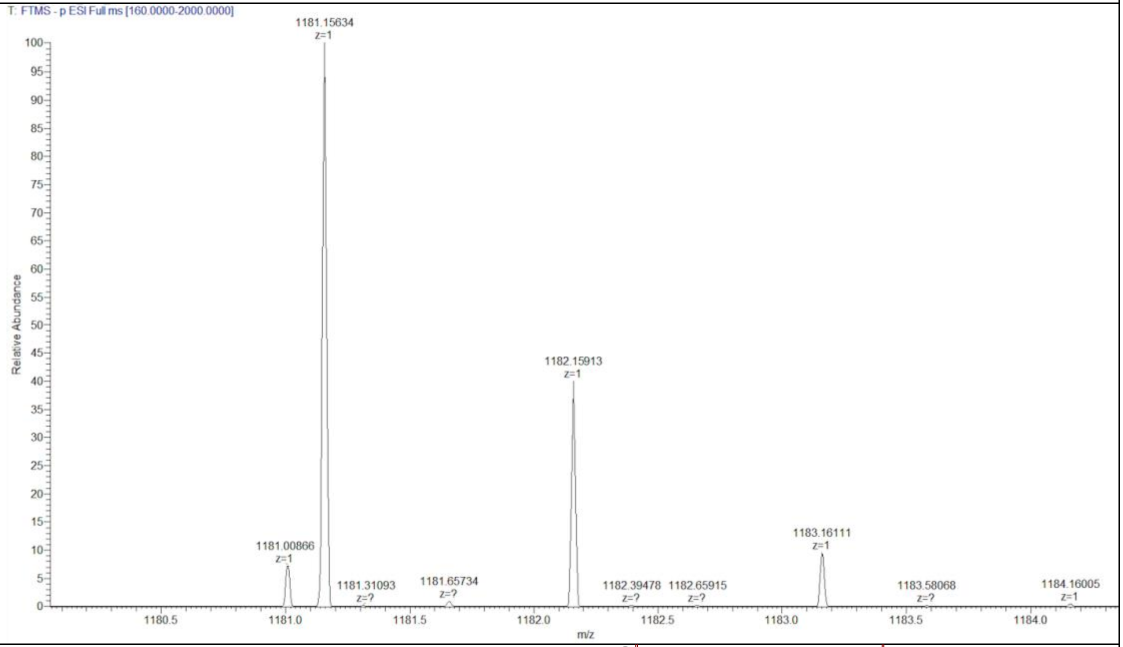

^{1}H NMR

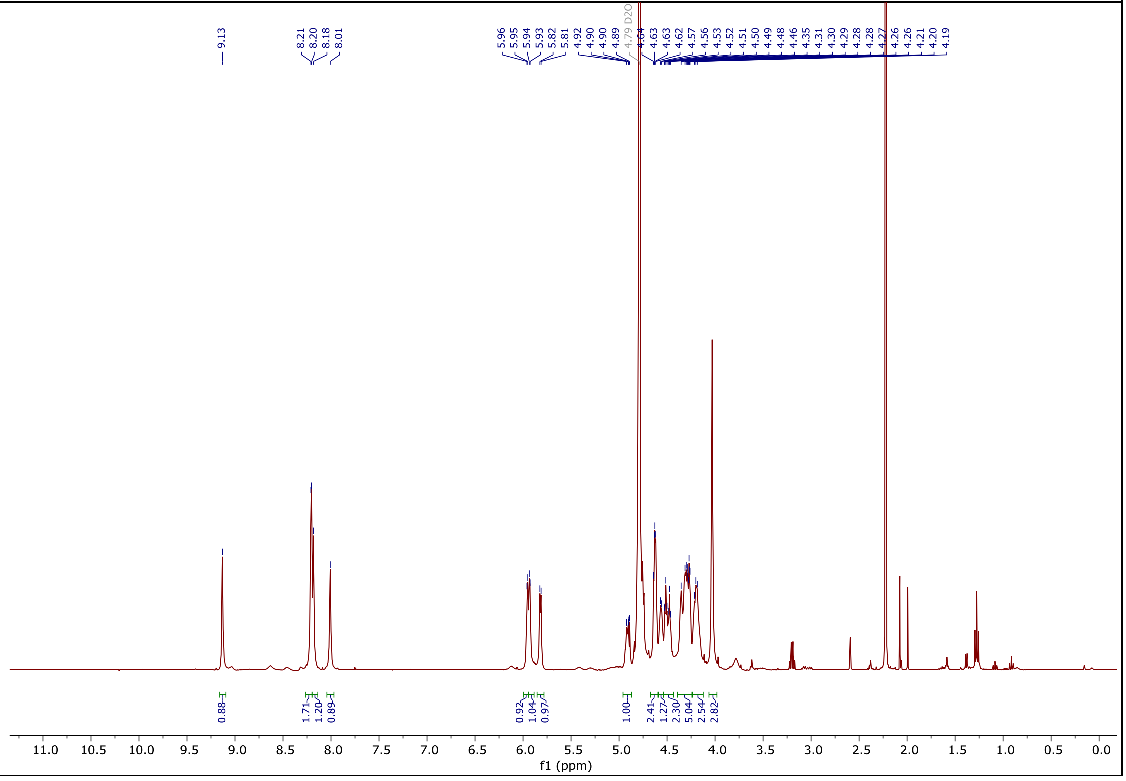

^{31}P NMR

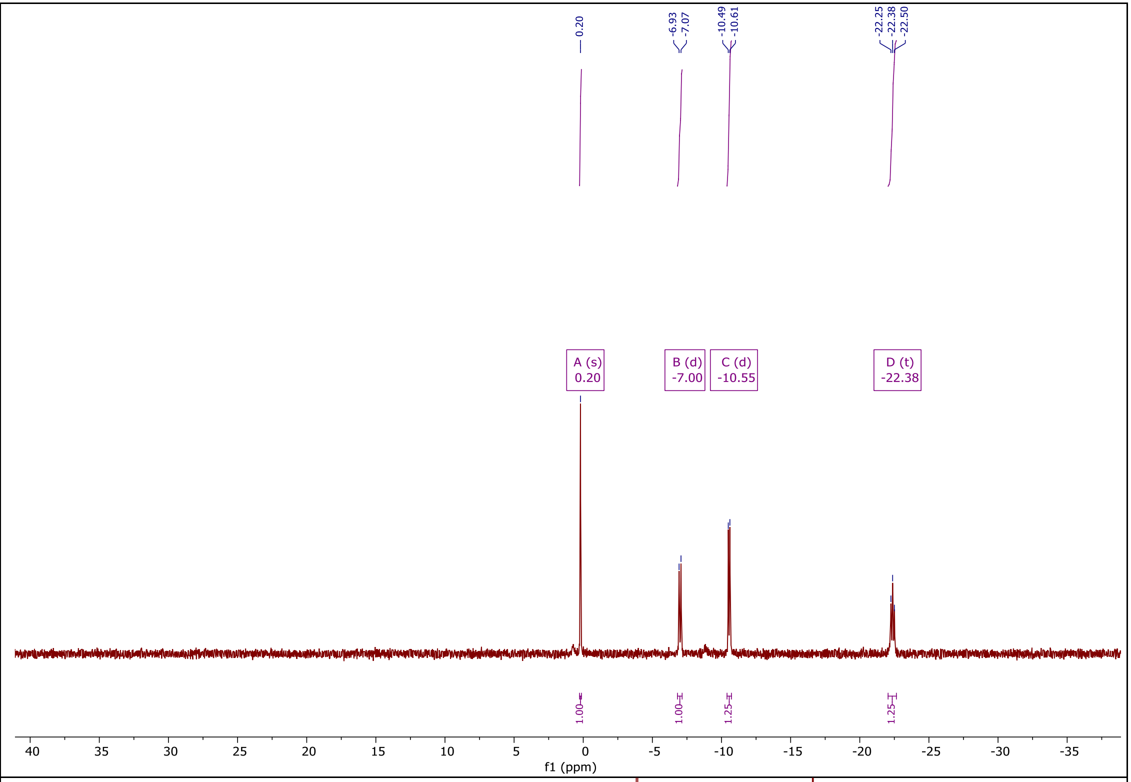

^{1}H-^{1}H COSY NMR

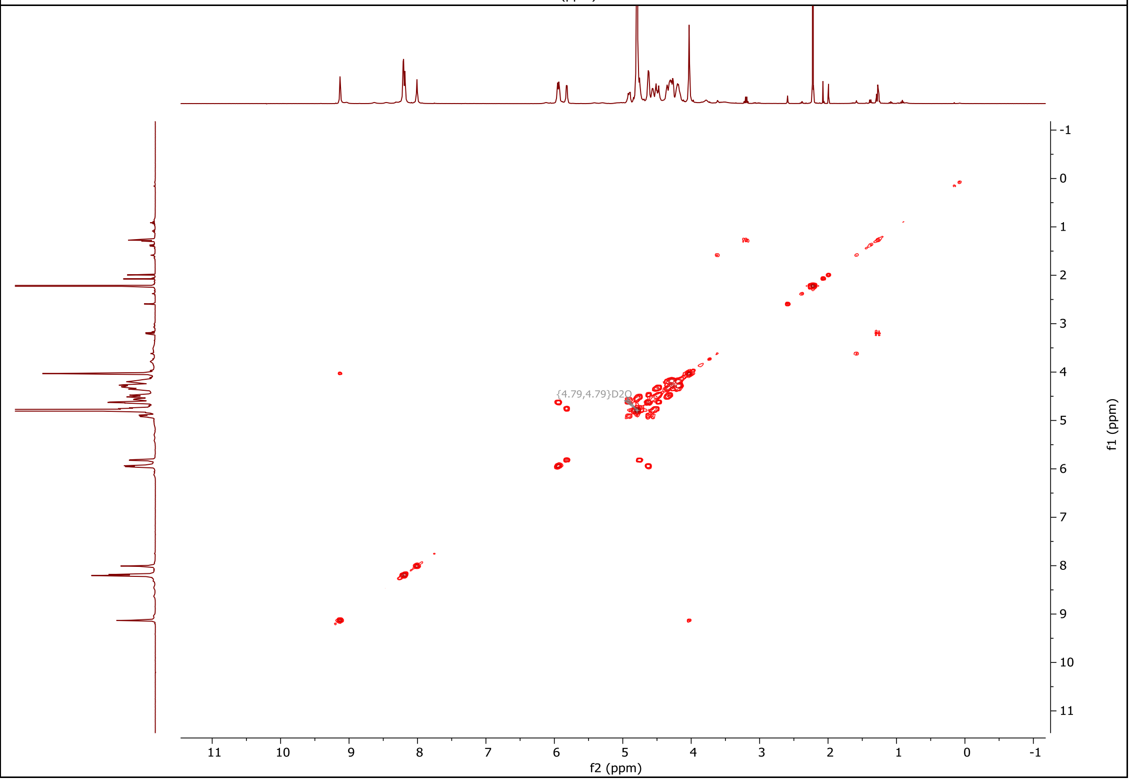

m⁷Gppp-tr-AmpG 2

Structure

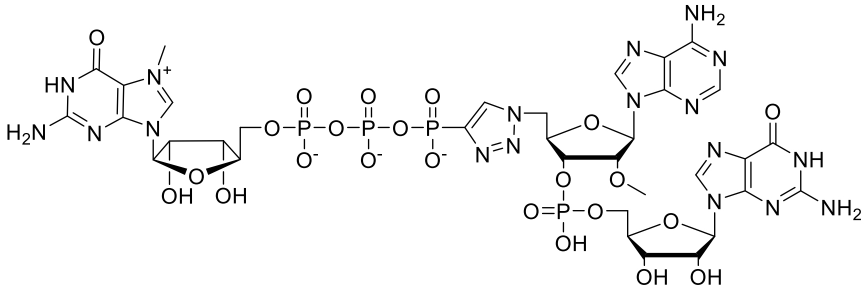

HPLC Profile

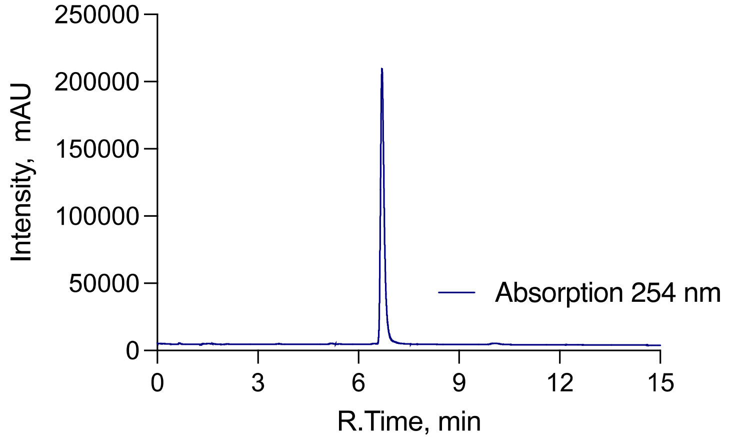

HR MS

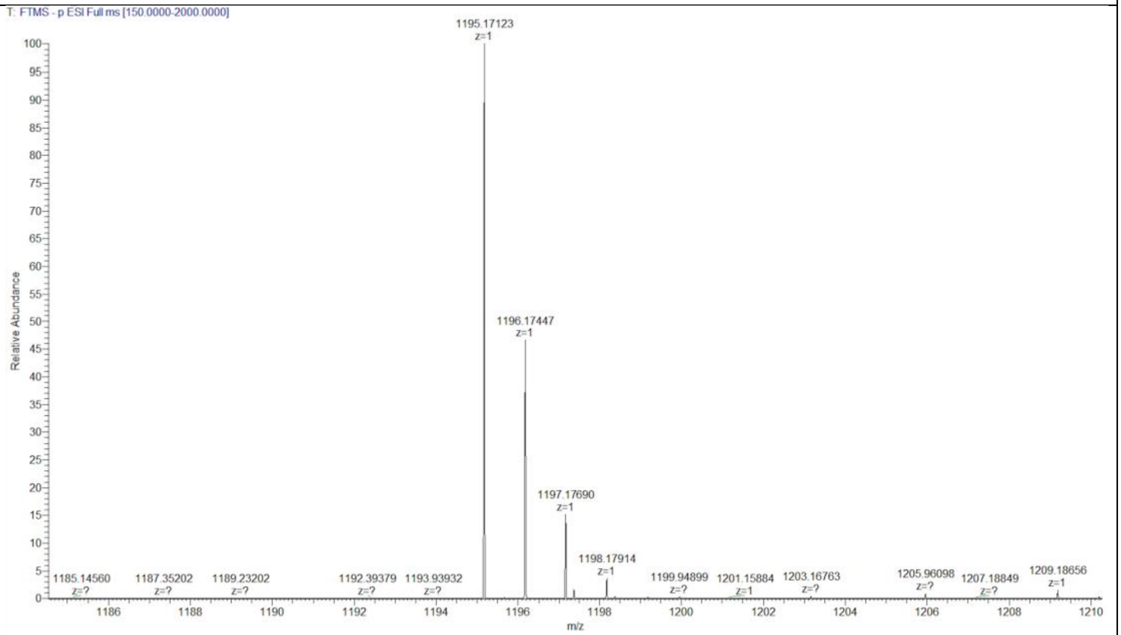

^{1}H NMR

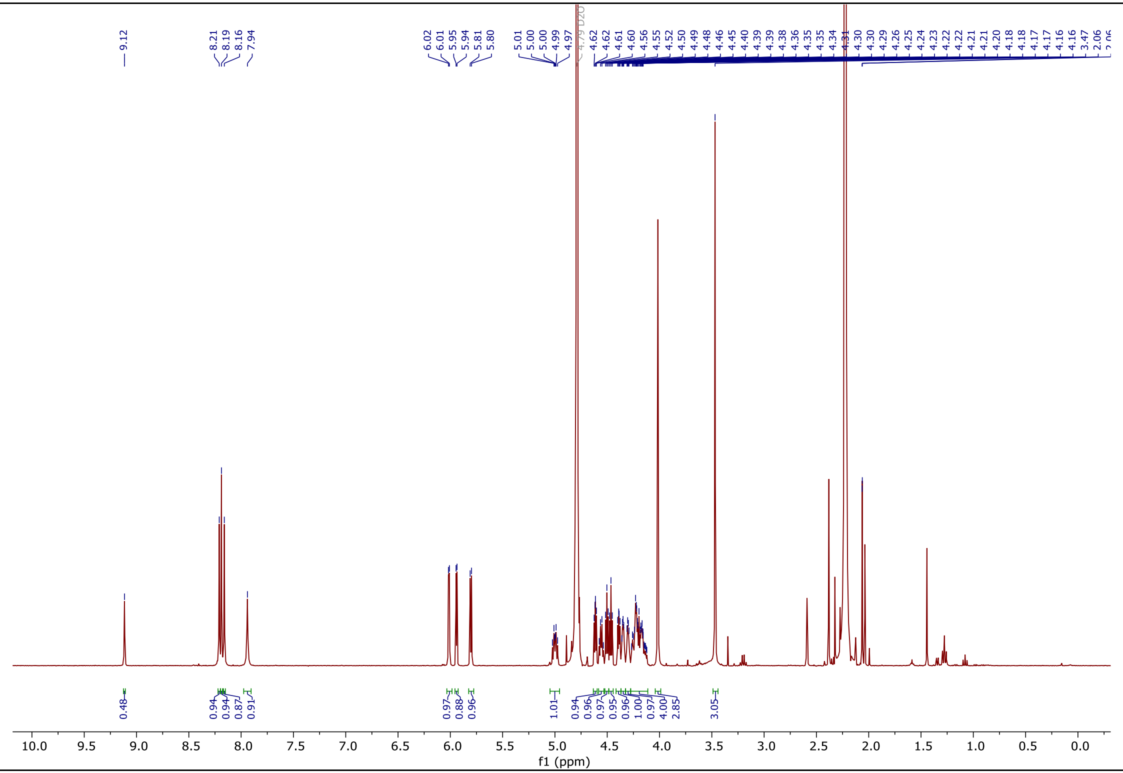

^{31}P NMR

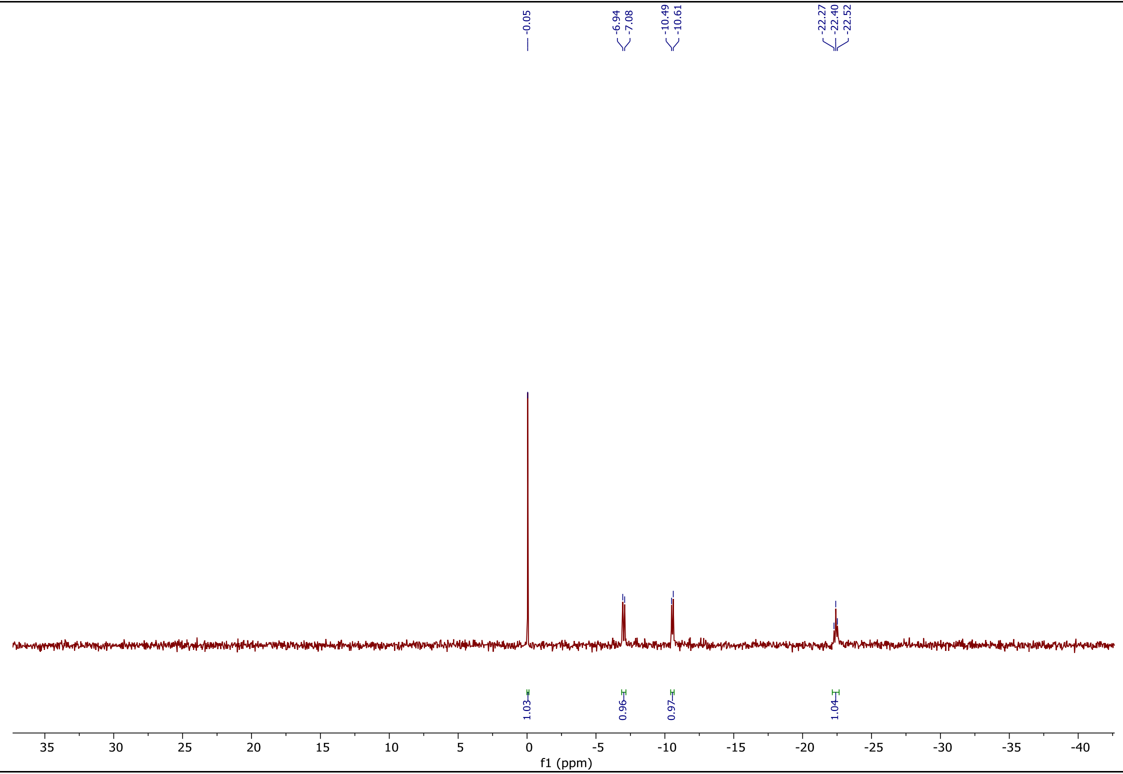

^1H-^1H COSY NMR

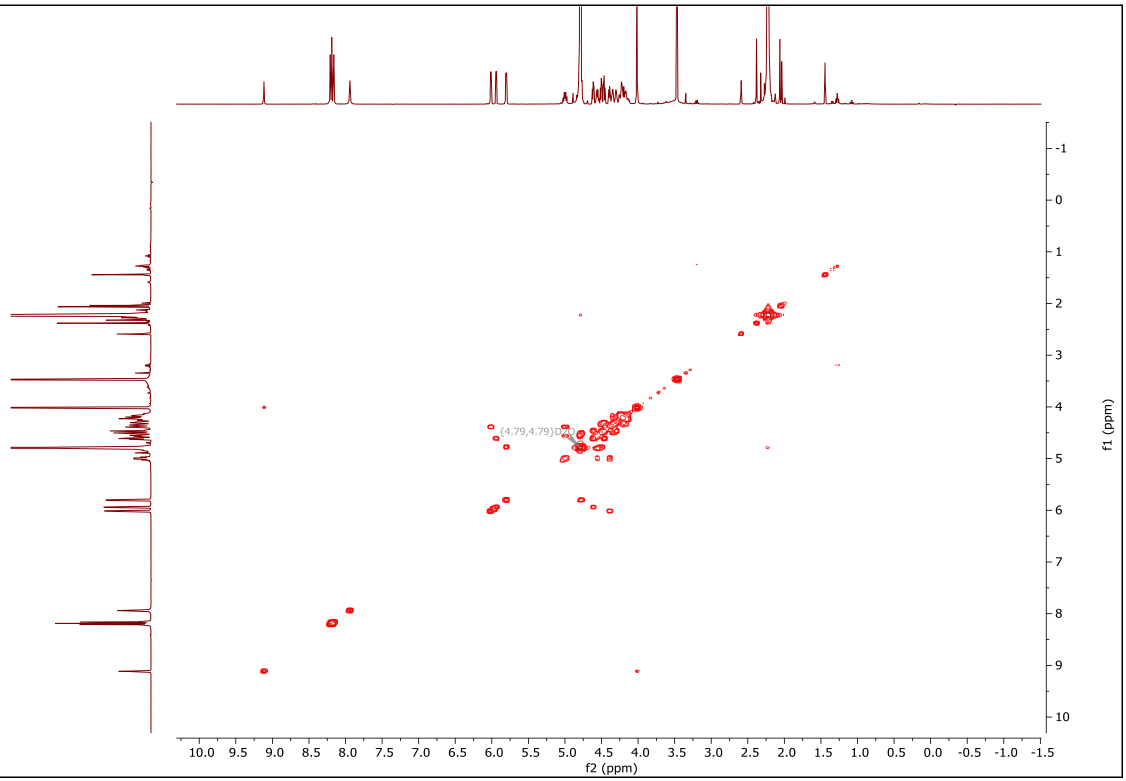

m[7]Gppp-CH[2]-tr-ApG 3

Structure

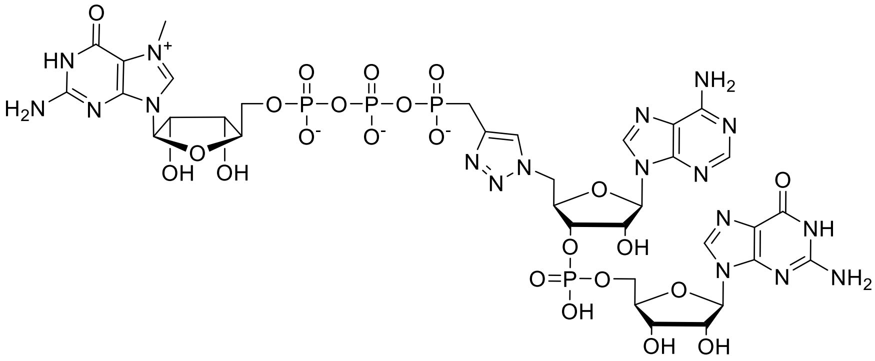

HPLC Profile

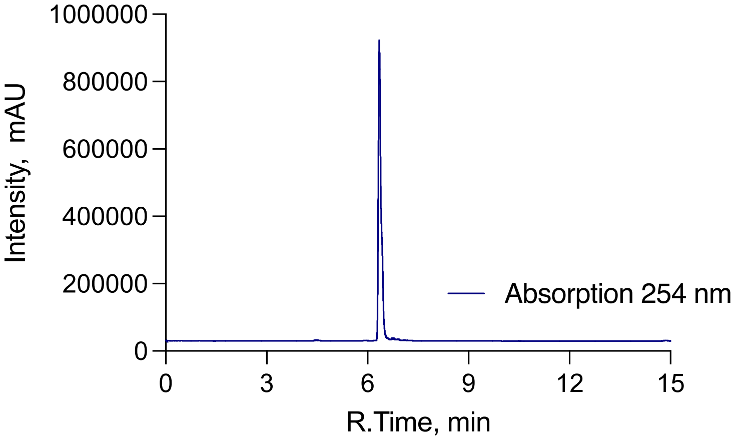

HR MS

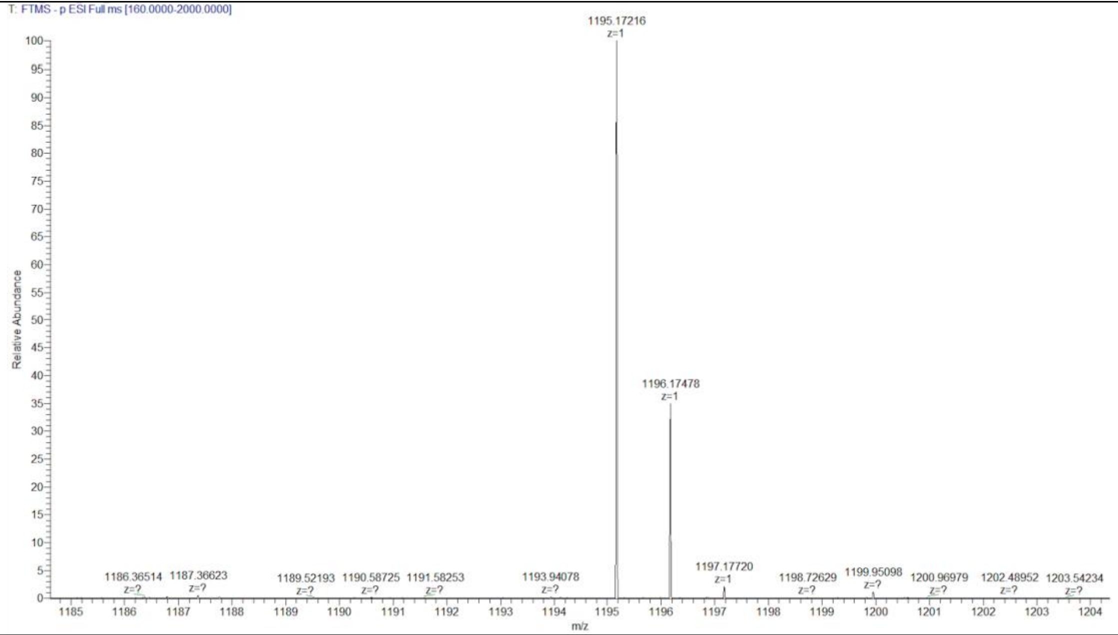
T: FTMS - p ESI Full ms [160.0000-2000.0000]
1195.17216
z=1
1196.17478
z=1
1197.17720
z=1
Relative Abundance
m/z

^{1}H NMR

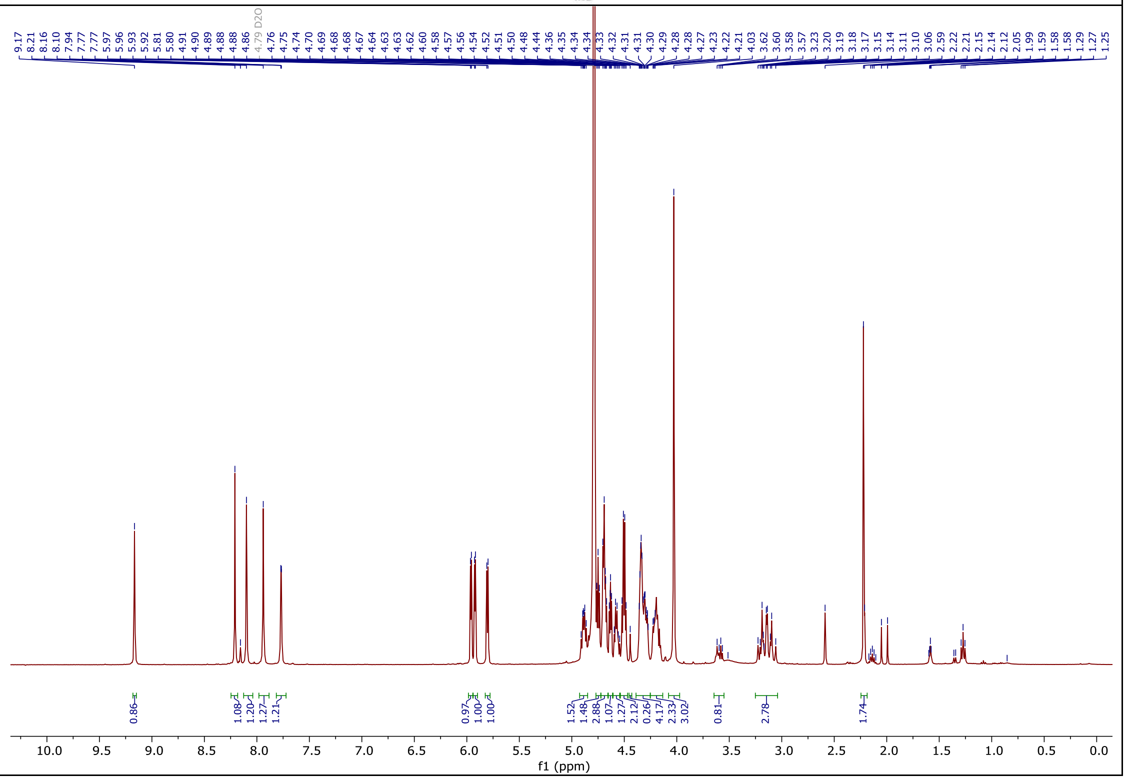
D2O
f1 (ppm)

^{31}P NMR

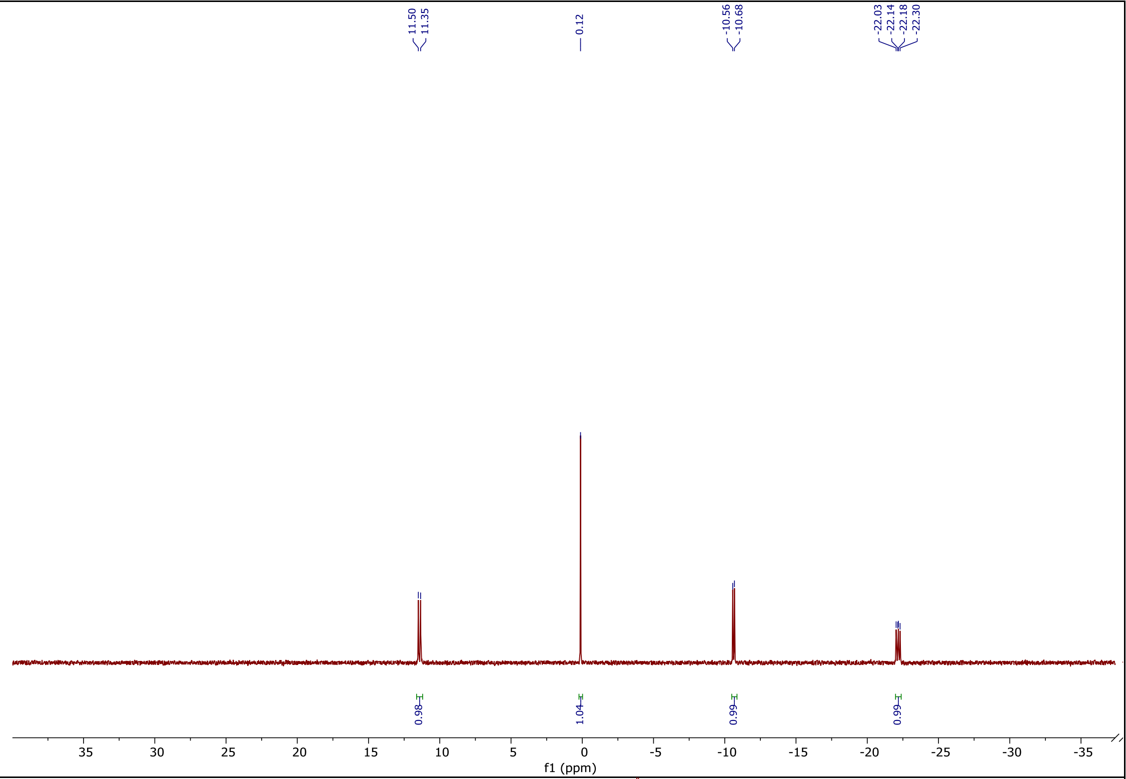

^{1}H-^{1}H COSY NMR

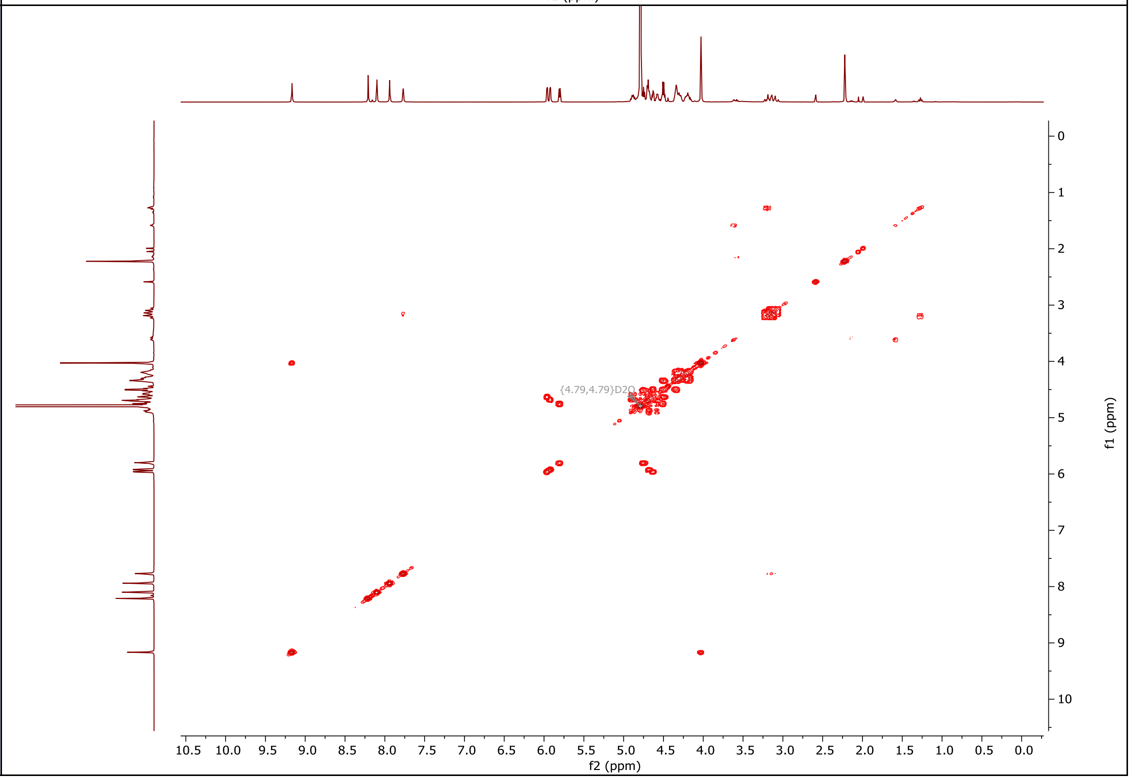

m^7Gppp-CH_2-tr-A_mpG 4

Structure

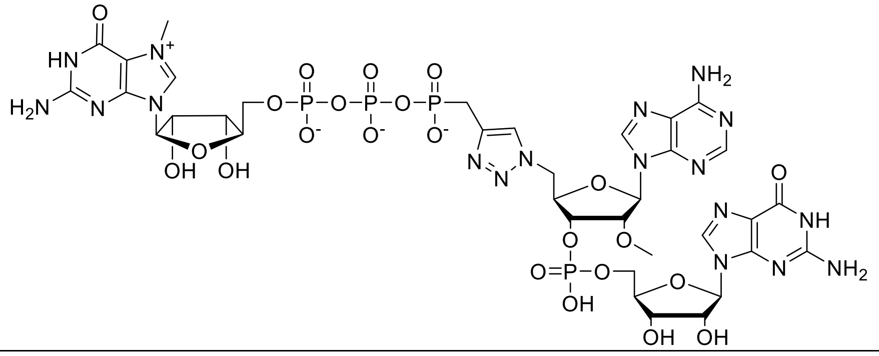

HPLC Profile

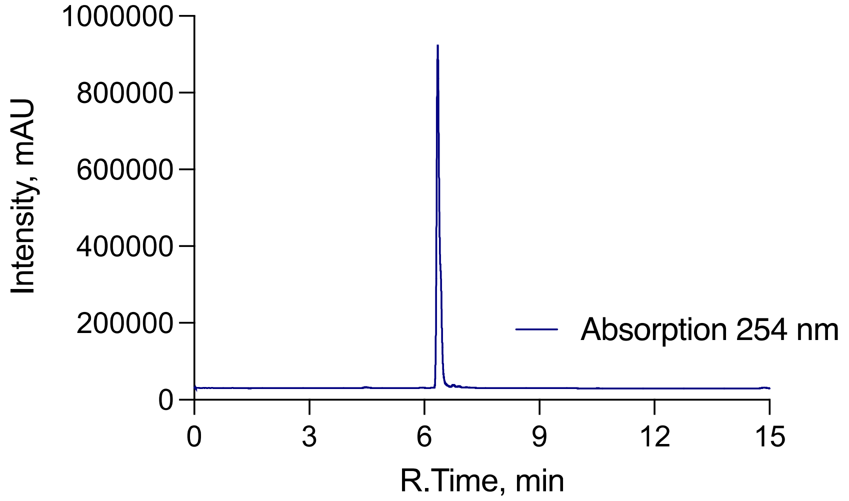

HR MS

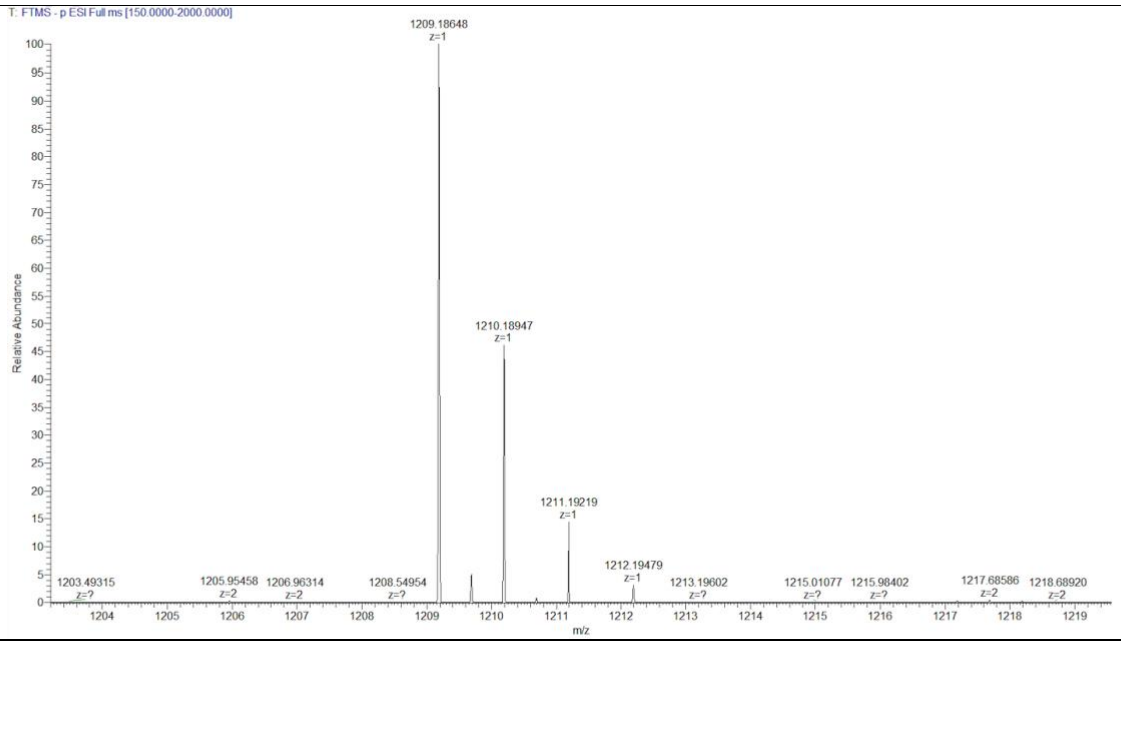

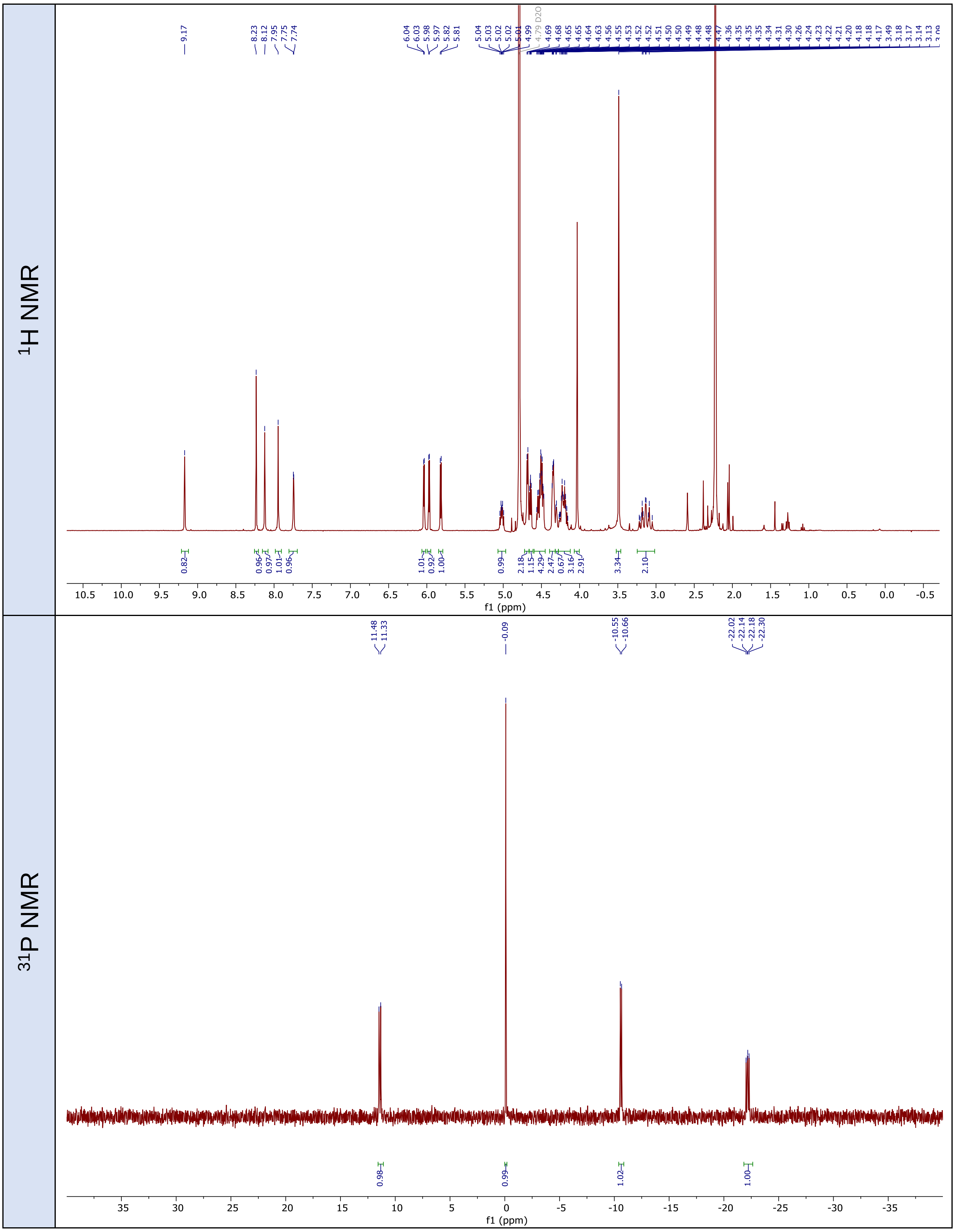

^{1}H NMR
^{31}P NMR
f1 (ppm)
f1 (ppm)

^1H-^1H COSY NMR

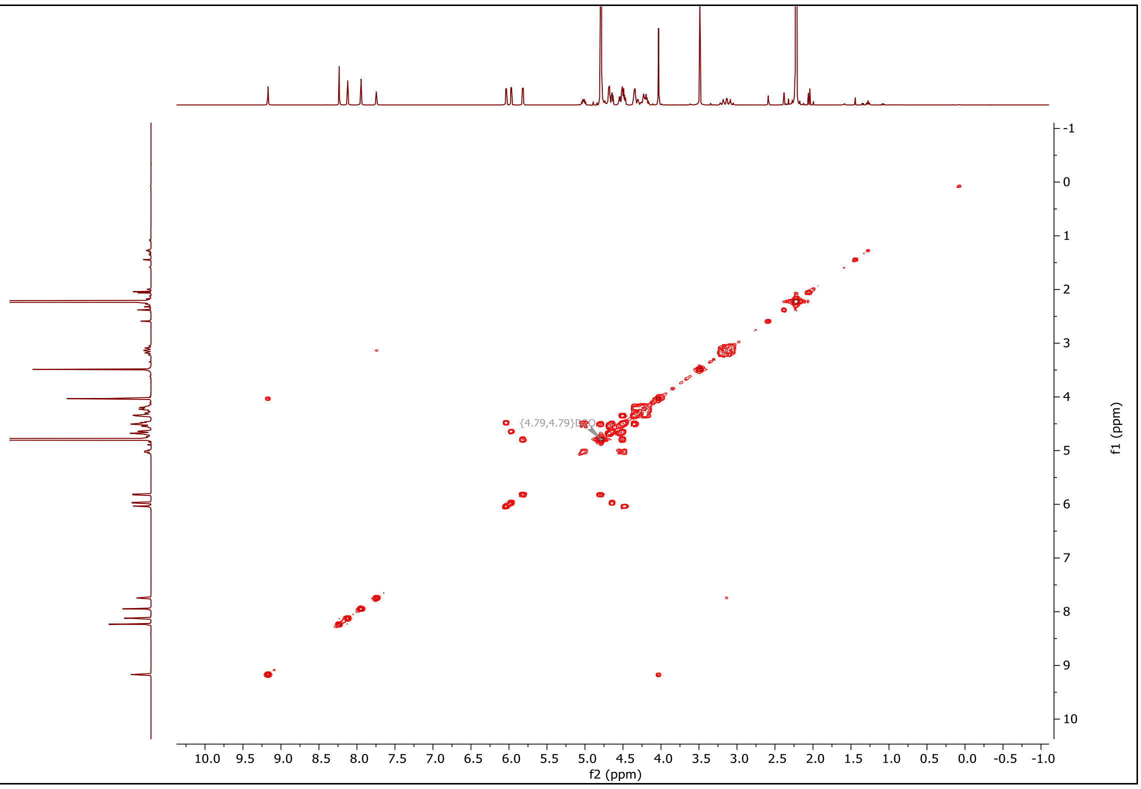

m^7Gppp-C_2H_4-ApG 5

Structure

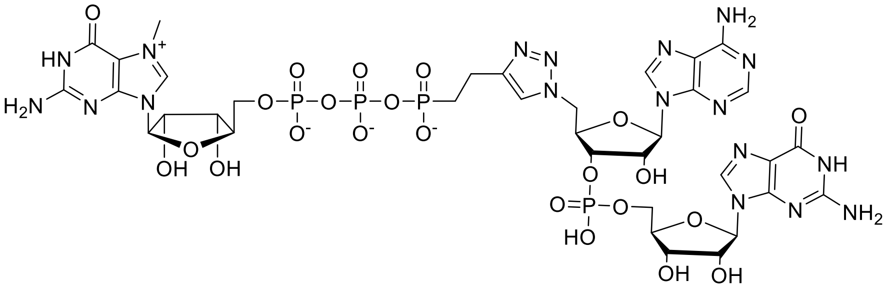

HPLC Profile

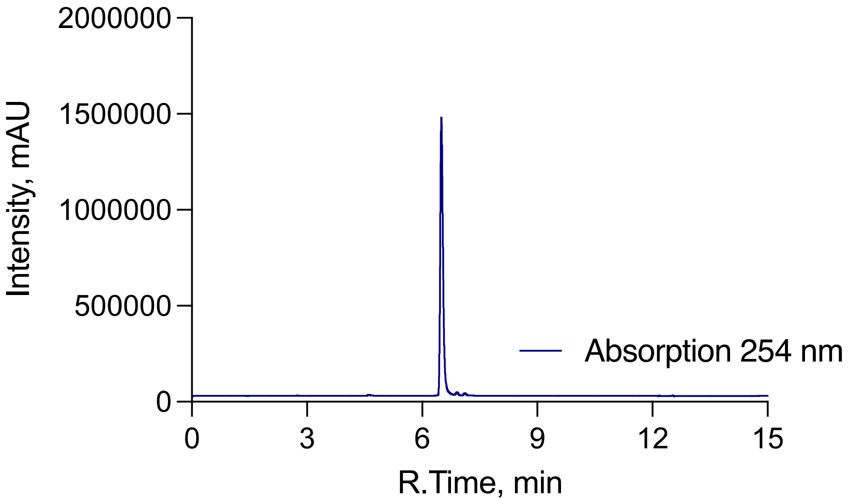

HR MS

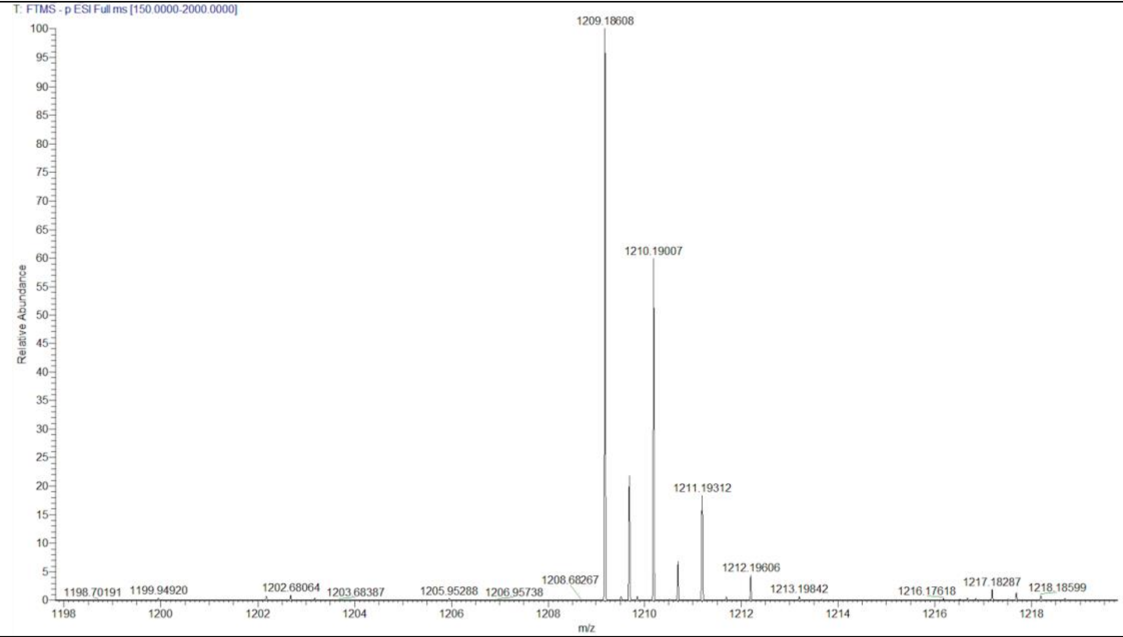

T: FTMS - p ESI Full ms [150.0000-2000.0000]
1209.18608
1210.19007
1211.19312
1212.19606
1213.19842
1198.70191
1199.94920
1202.68064
1203.68387
1205.95288
1206.95738
1208.68267
1216.17618
1217.18287
1218.18599
Relative Abundance
m/z

^{1}H NMR

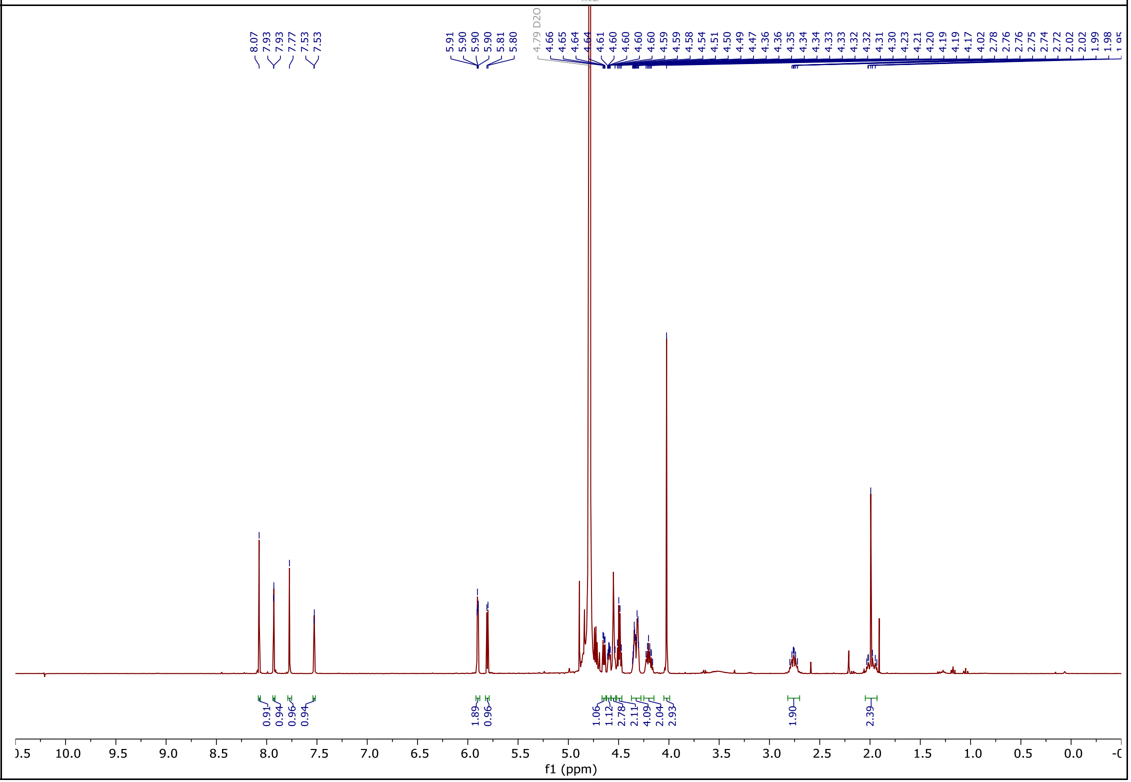

4.79 D2O
f1 (ppm)

^{31}P NMR

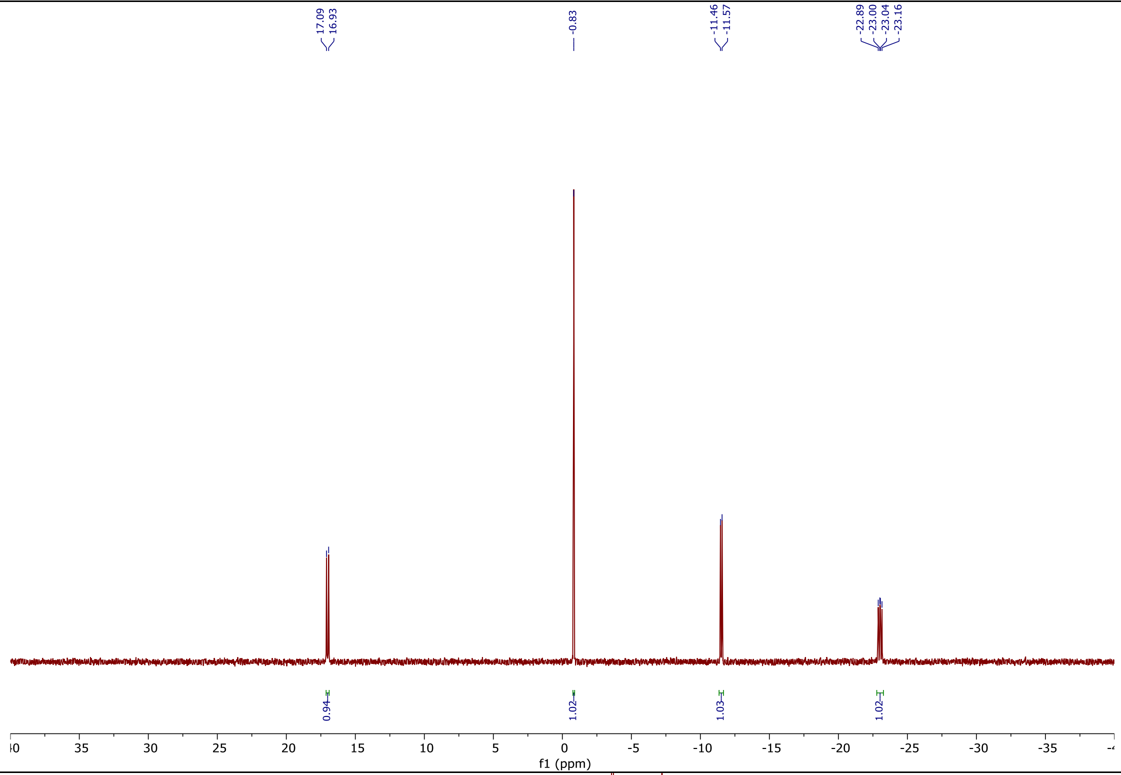

^{1}H-^{1}H COSY NMR

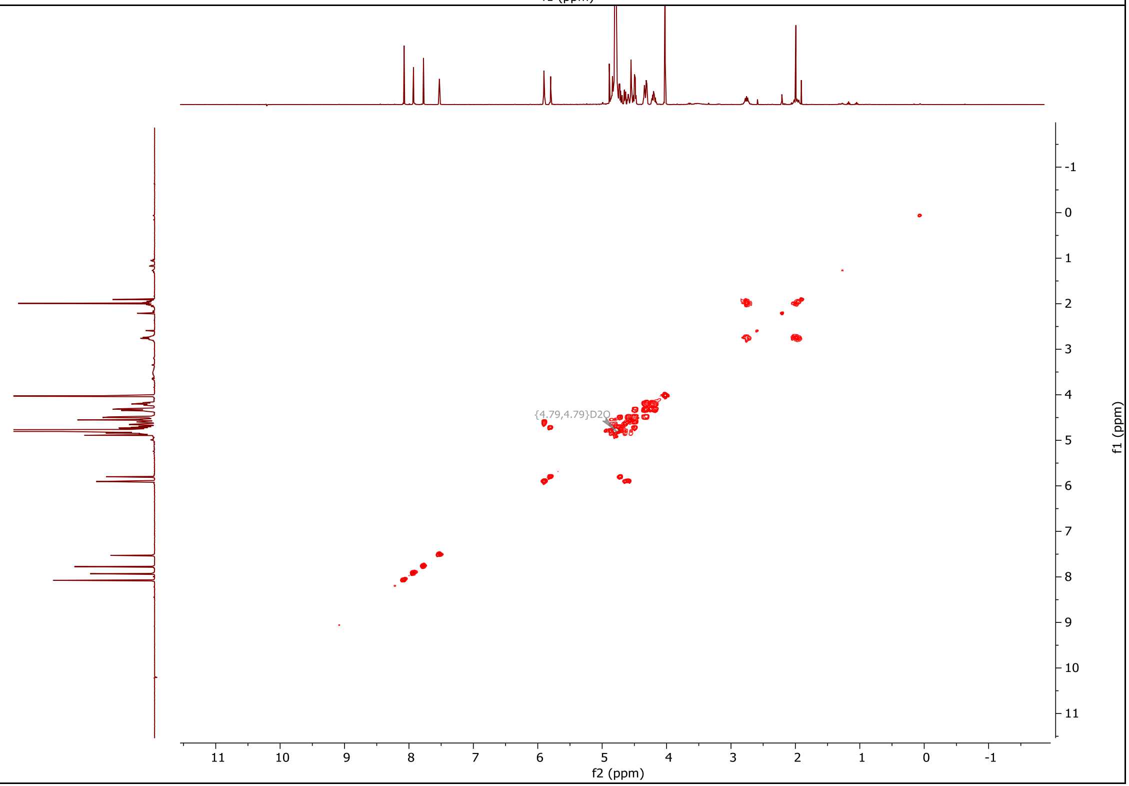

^{1}H-^{13}C HSQC NMR

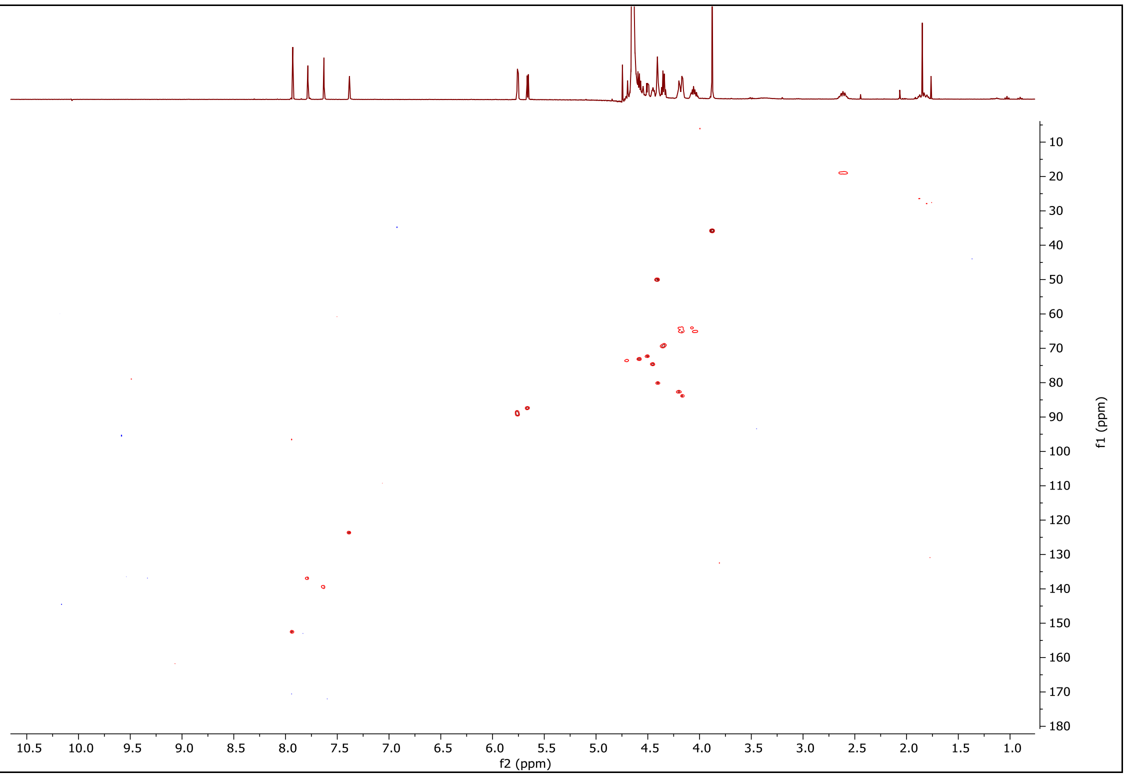

m^7Gppp-C_2H_4-A_mpG 6

Structure

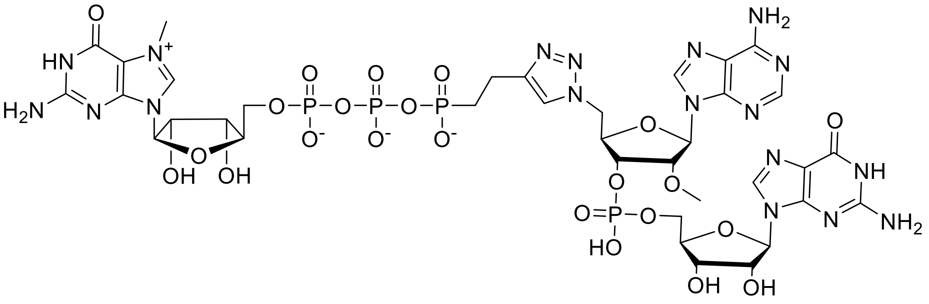

HPLC Profile

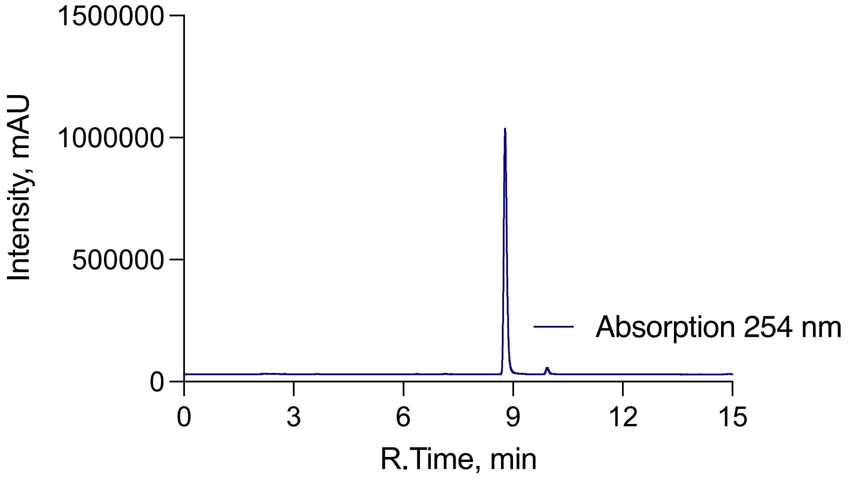

HR MS

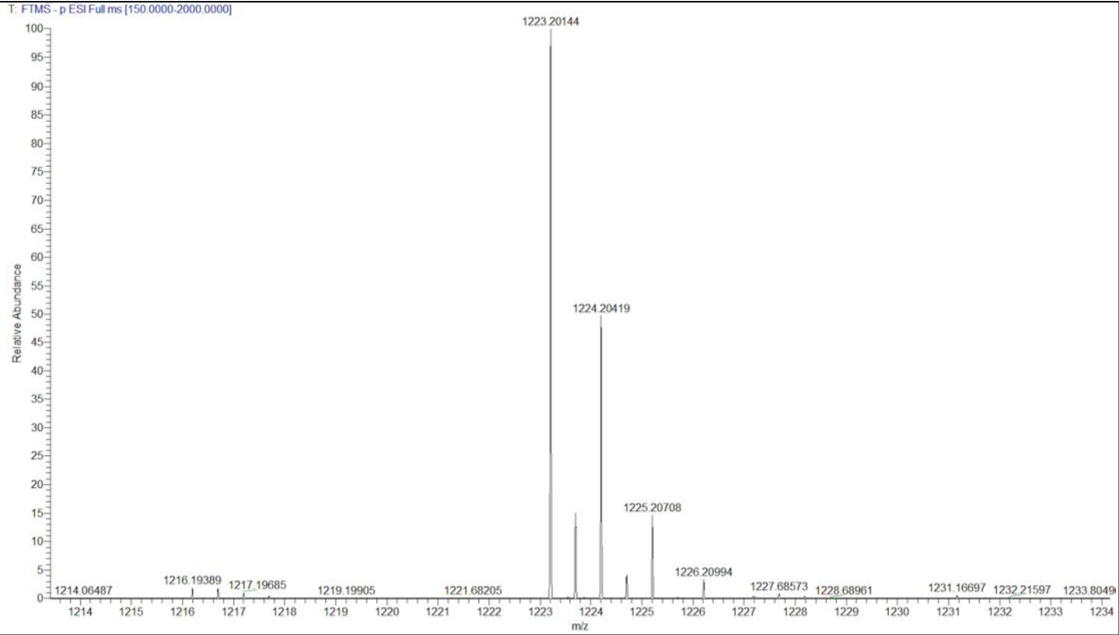

T: FTMS - p ESI Full ms [150.0000-2000.0000]
Relative Abundance
m/z
1223.20144
1224.20419
1225.20708
1226.20994
1214.06487
1216.19389
1217.19685
1219.19905
1221.68205
1227.68573
1228.68961
1231.16697
1232.21597

^{1}H NMR

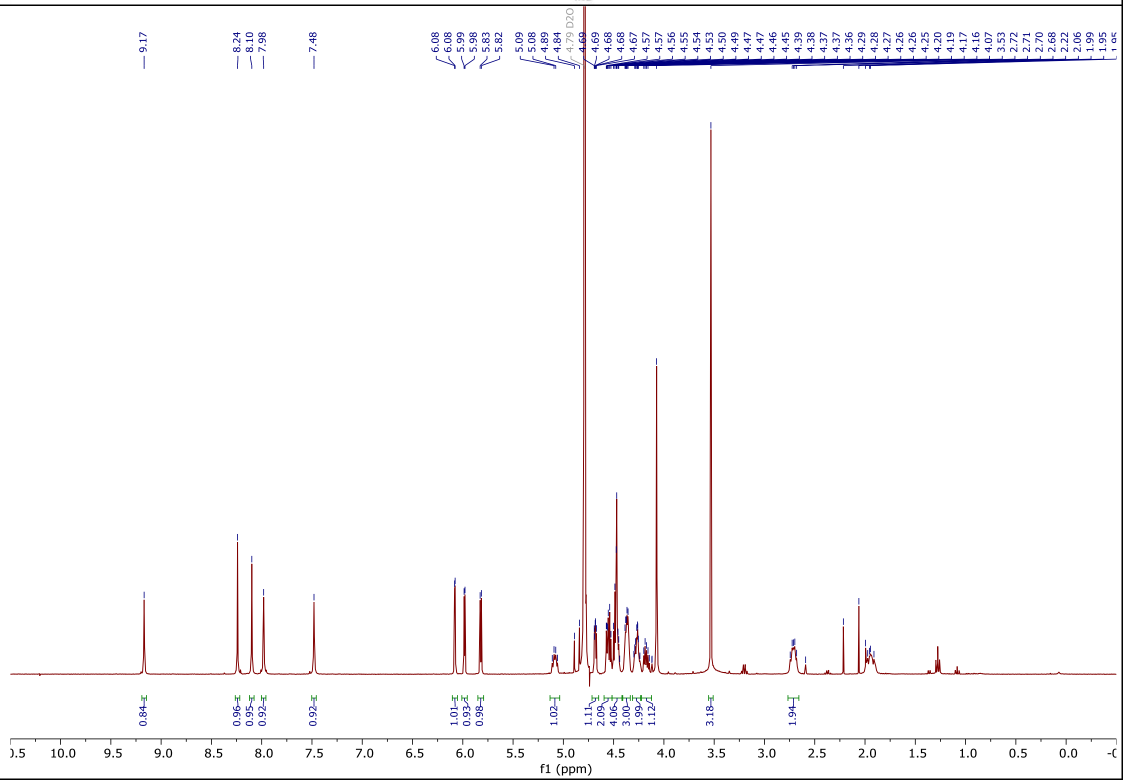

9.17
8.24
8.10
7.98
7.48
4.79 D2O
3.53
f1 (ppm)

^{31}P NMR

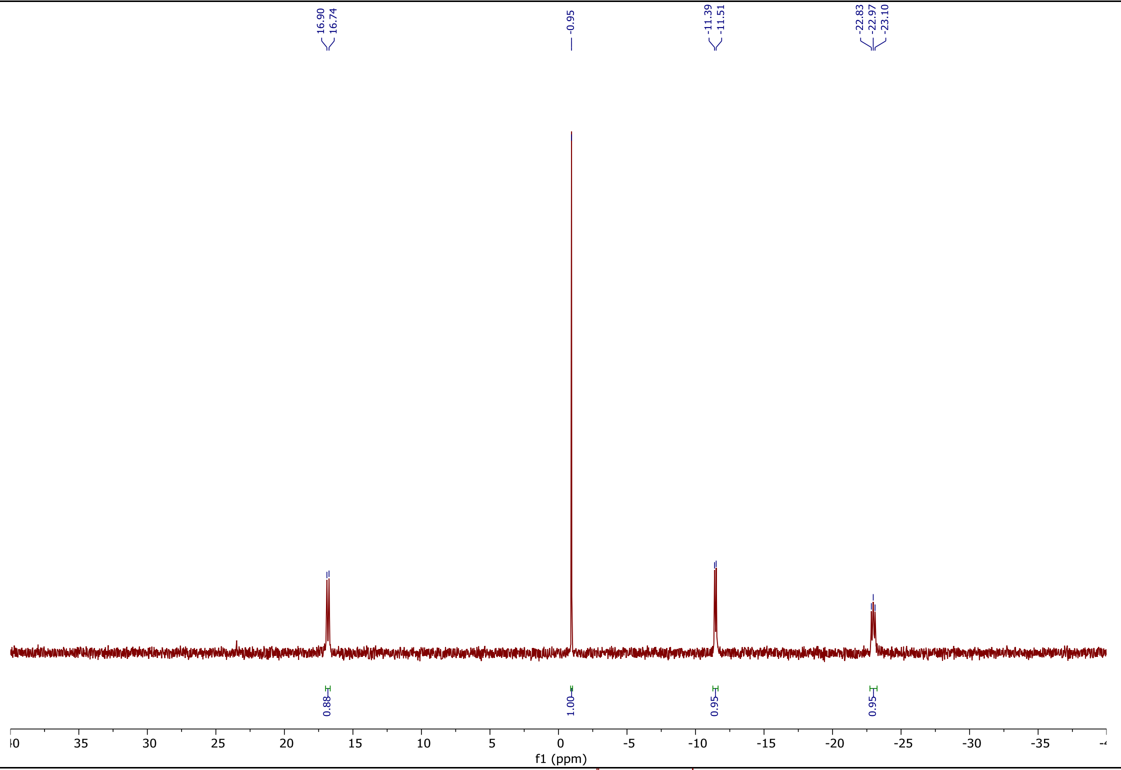

^{1}H-^{1}H COSY NMR

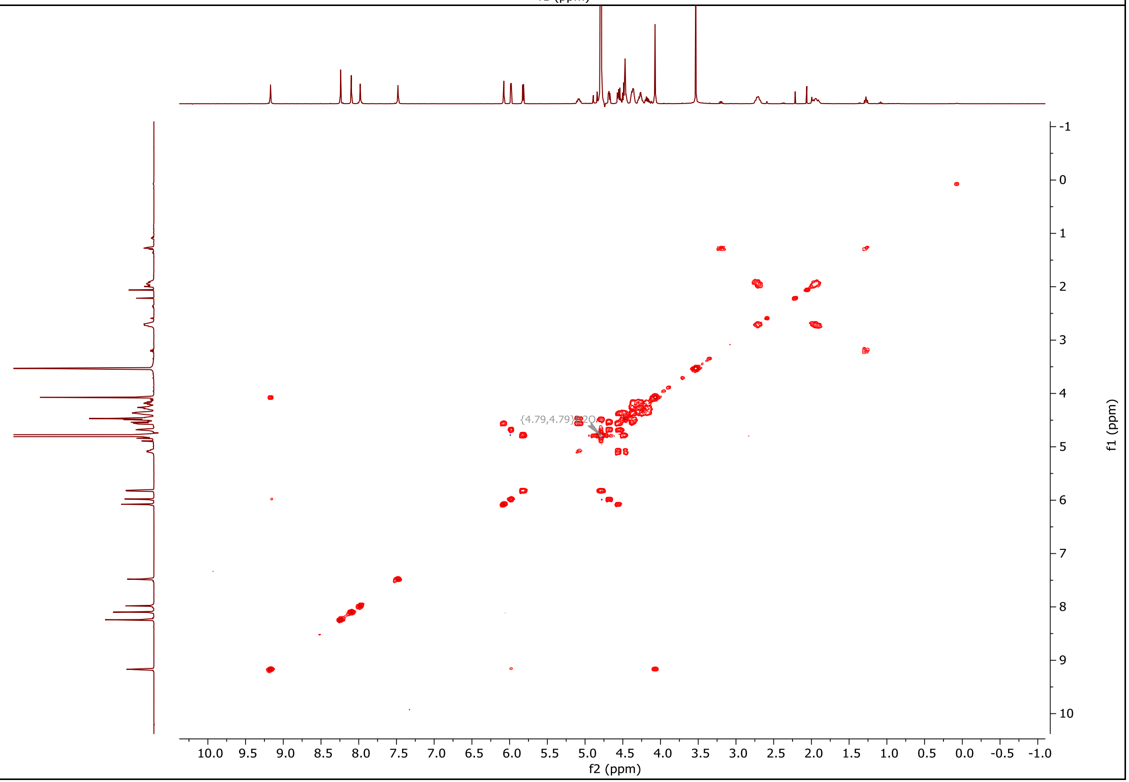

^1H-^{13}C HSQC NMR

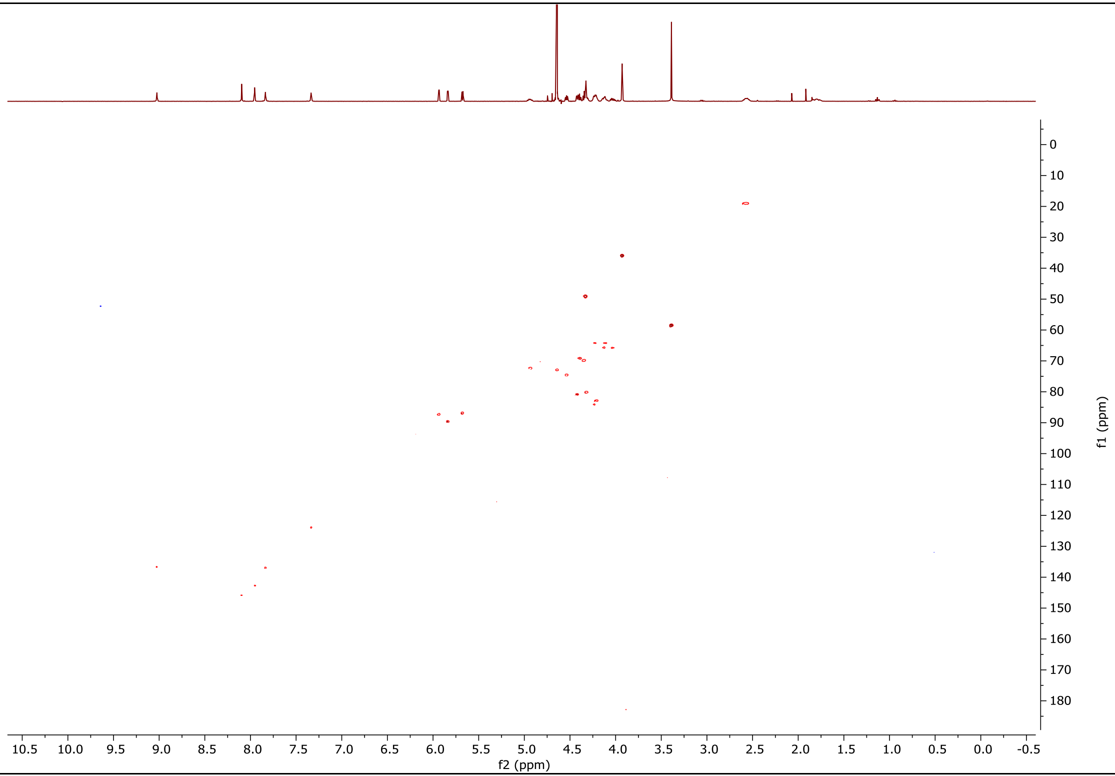

m7GpppOCH2-tr-ApG 7

Structure

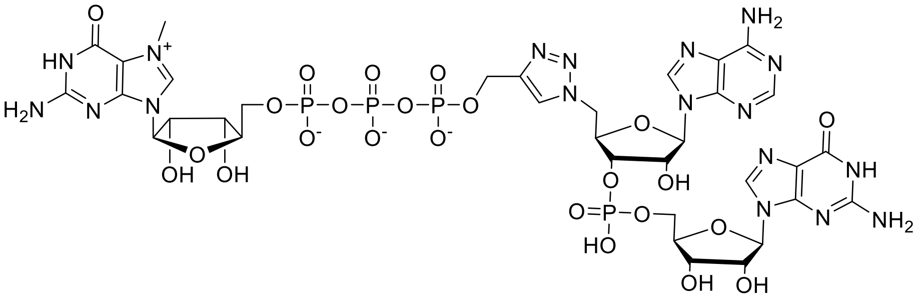

HPLC Profile

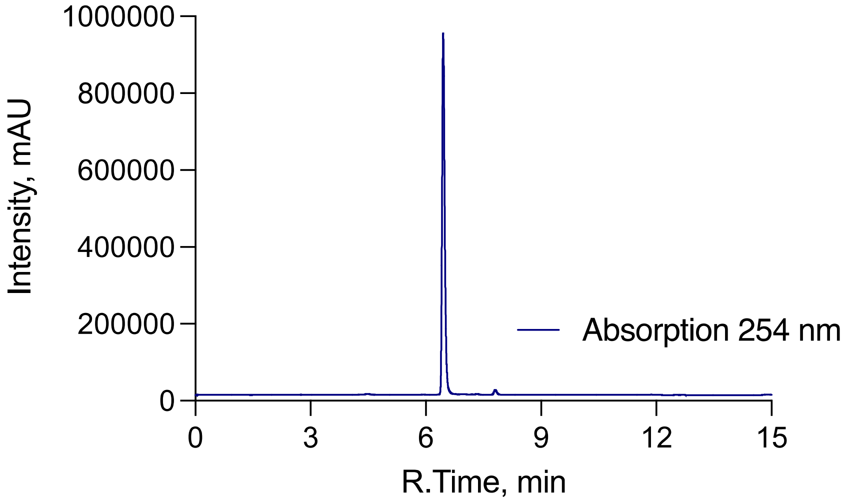

HR MS

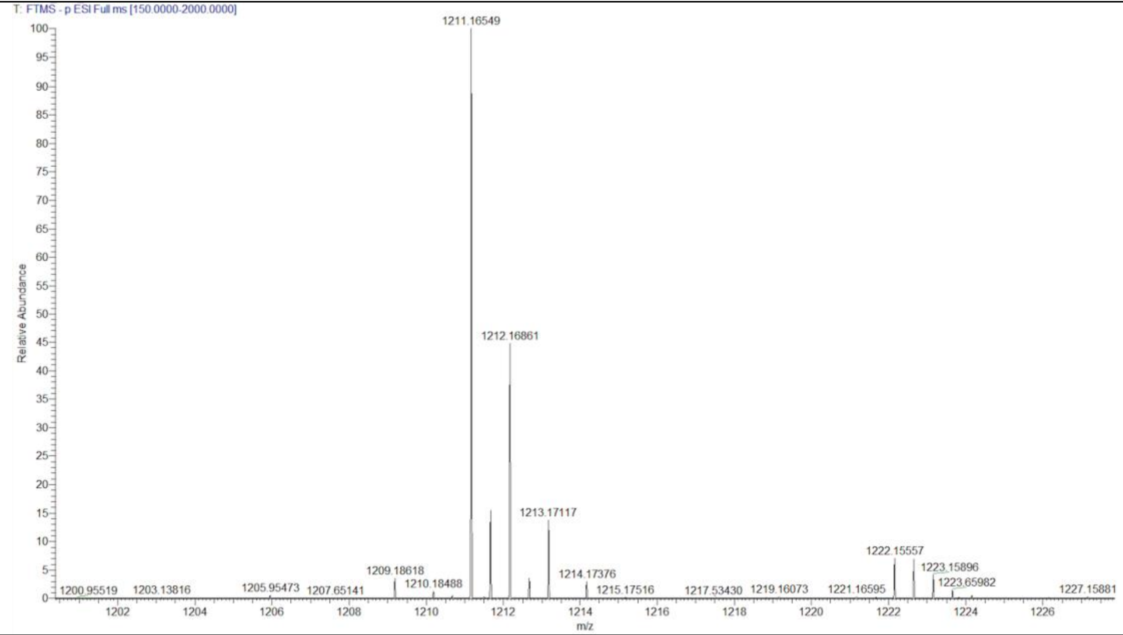
T: FTMS - p ESI Full ms [150.0000-2000.0000]
1211.16549
1212.16861
1213.17117
1214.17376
1215.17516
1209.18618
1210.18488
1200.95519
1203.13816
1205.95473
1207.65141
1217.53430
1219.16073
1221.16595
1222.15557
1223.15896
1223.65982
1227.15881
Relative Abundance
m/z

^{1}H NMR

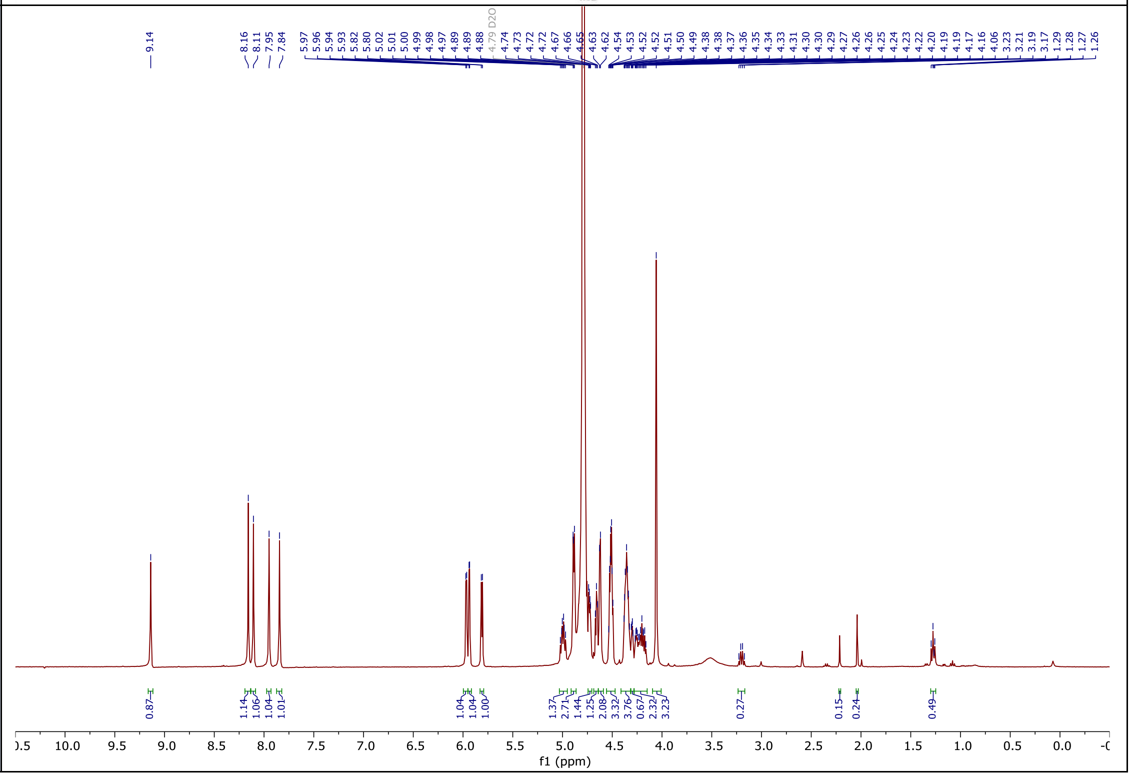
f1 (ppm)
4.79 D2O

^{31}P NMR

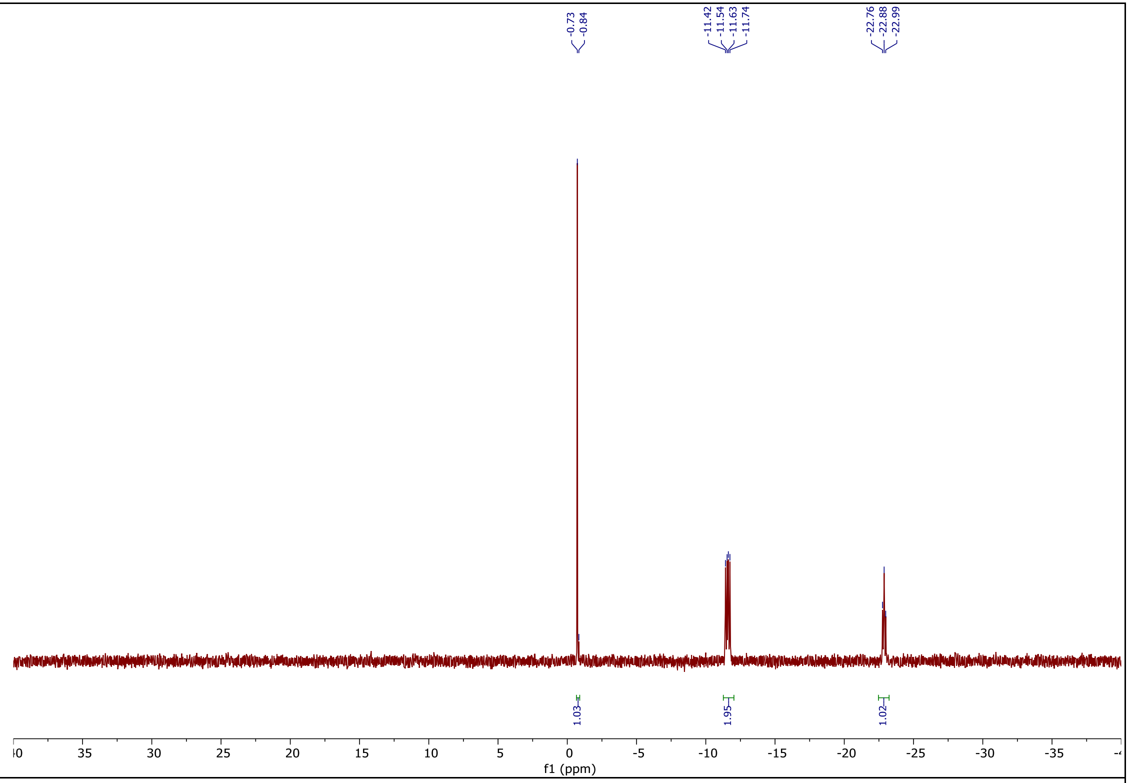

^{1}H-^{1}H COSY NMR

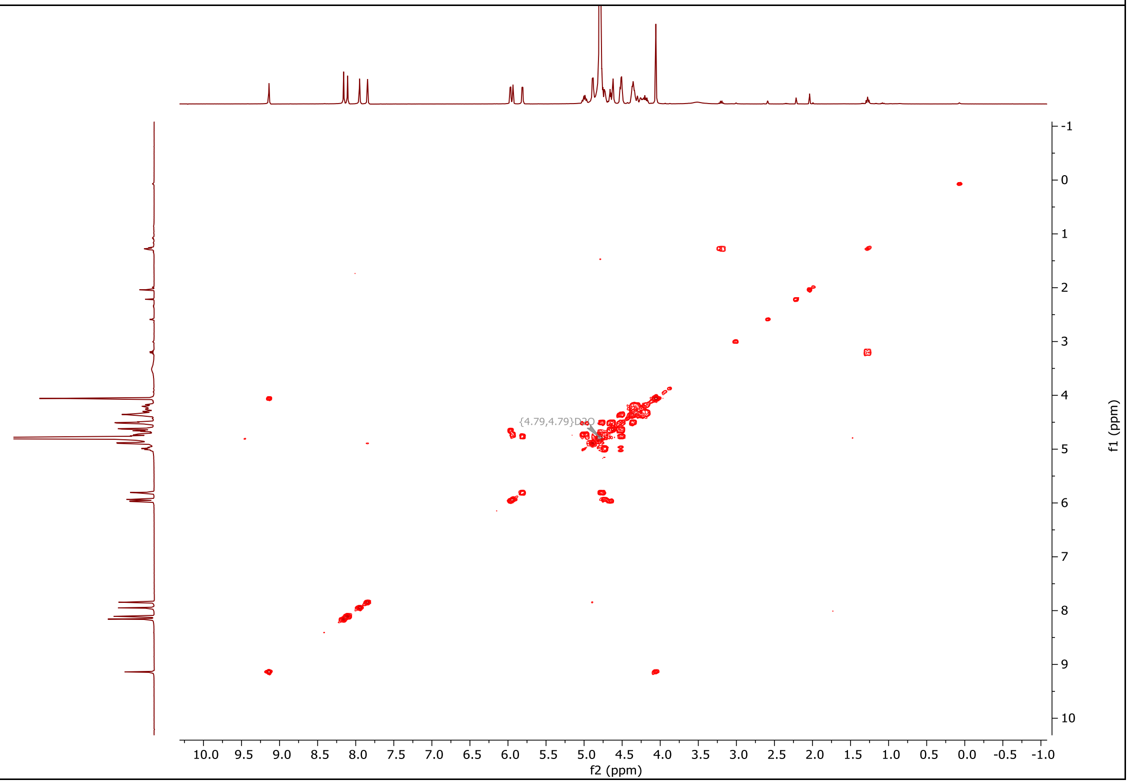

^{1}H-^{13}C HSQC NMR

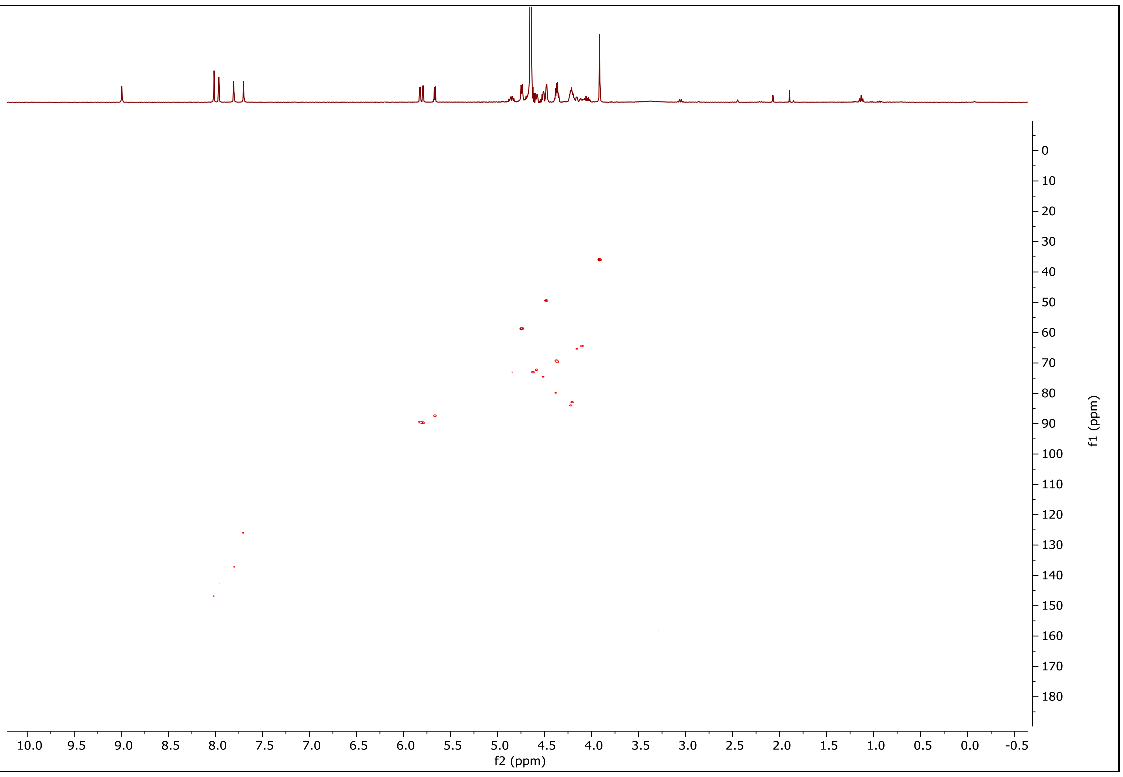

m^{7}GpppOCH$_2$-tr-A$_m$pG 8

Structure

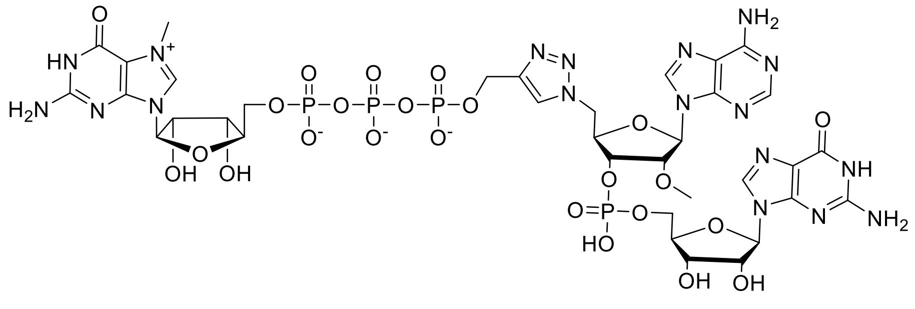

HPLC Profile

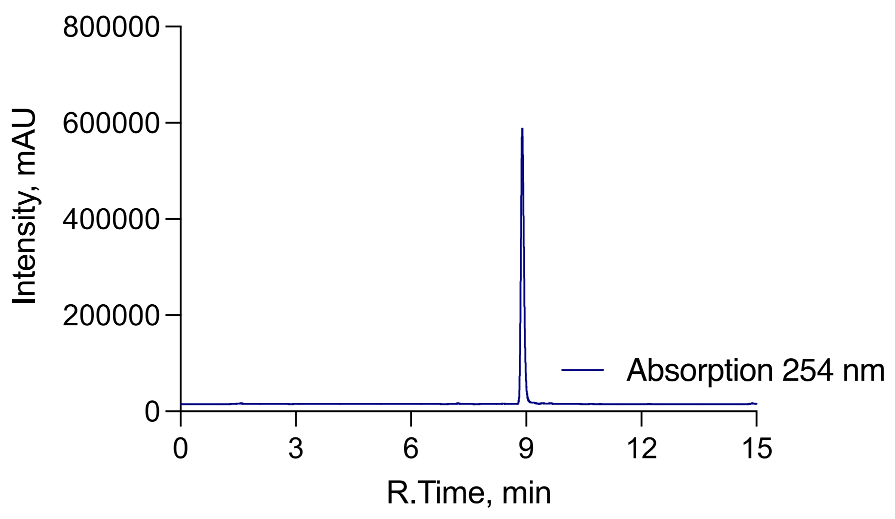

HR MS

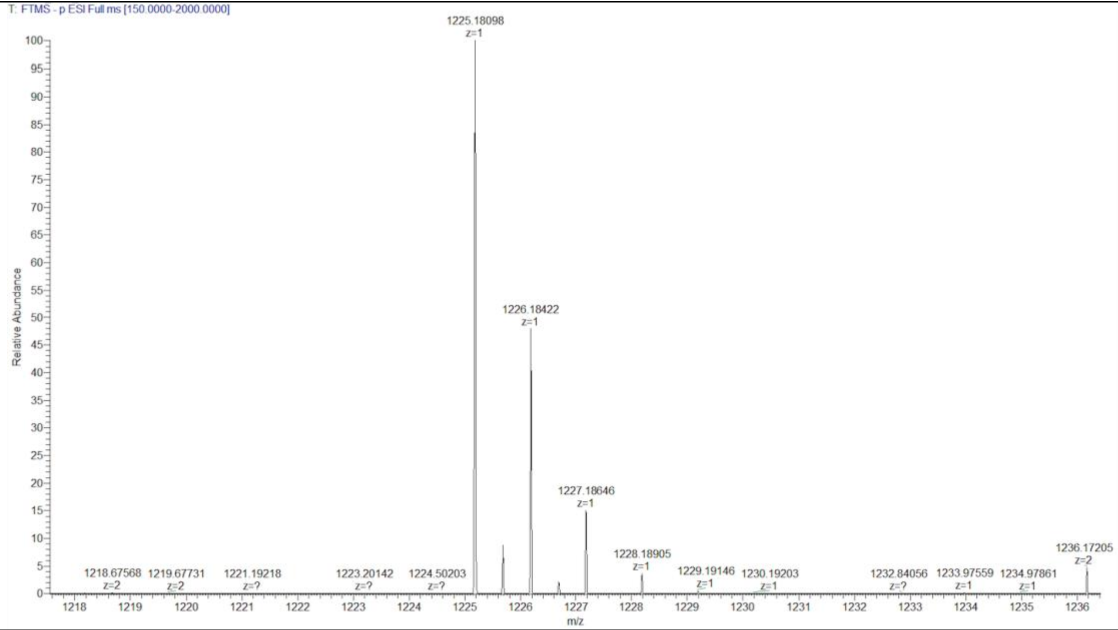
T: FTMS - p ESI Full ms [150.0000-2000.0000]
1225.18098
z=1
1226.18422
z=1
1227.18646
z=1
1228.18905
z=1
1236.17205
z=2
Relative Abundance
m/z

^{1}H NMR

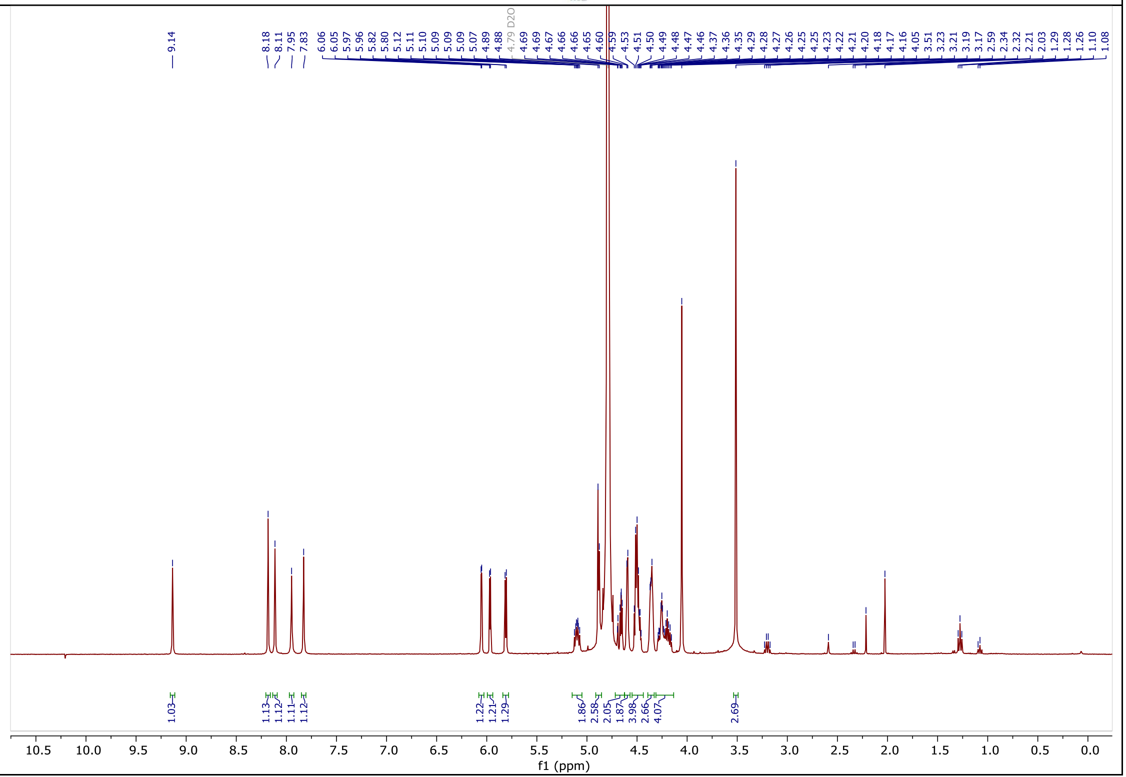
4.79 D2O
f1 (ppm)

^{31}P NMR

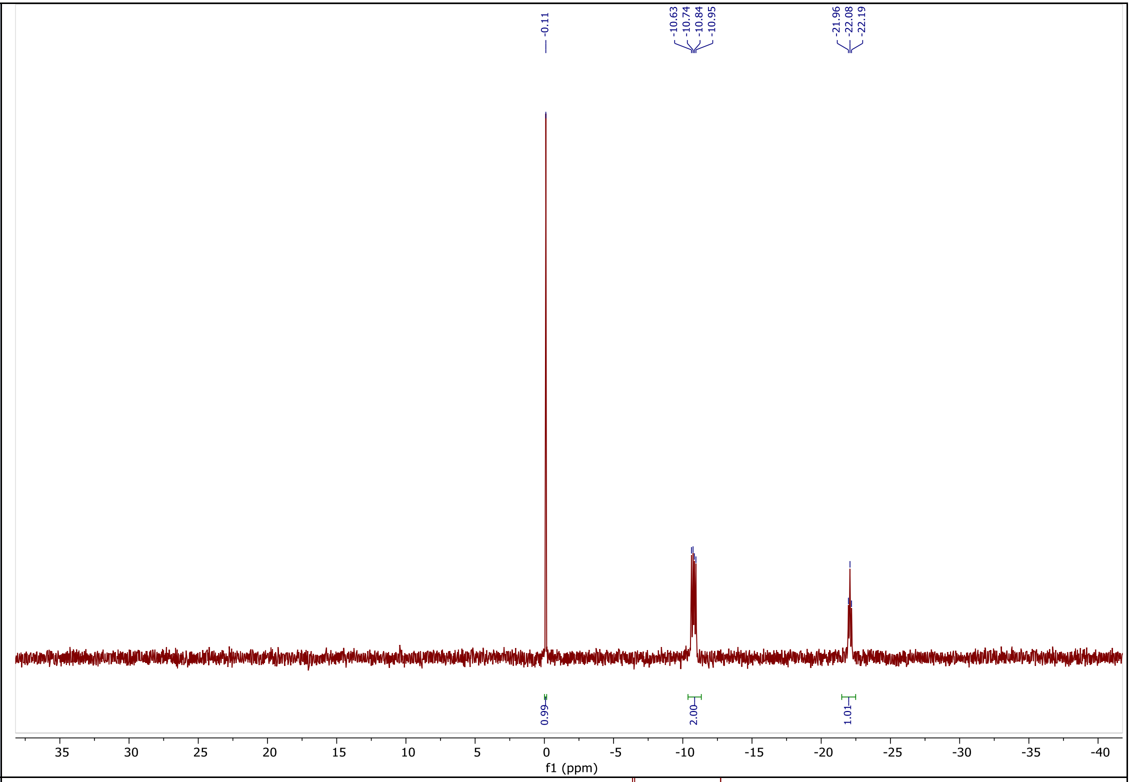

^{1}H-^{1}H COSY NMR

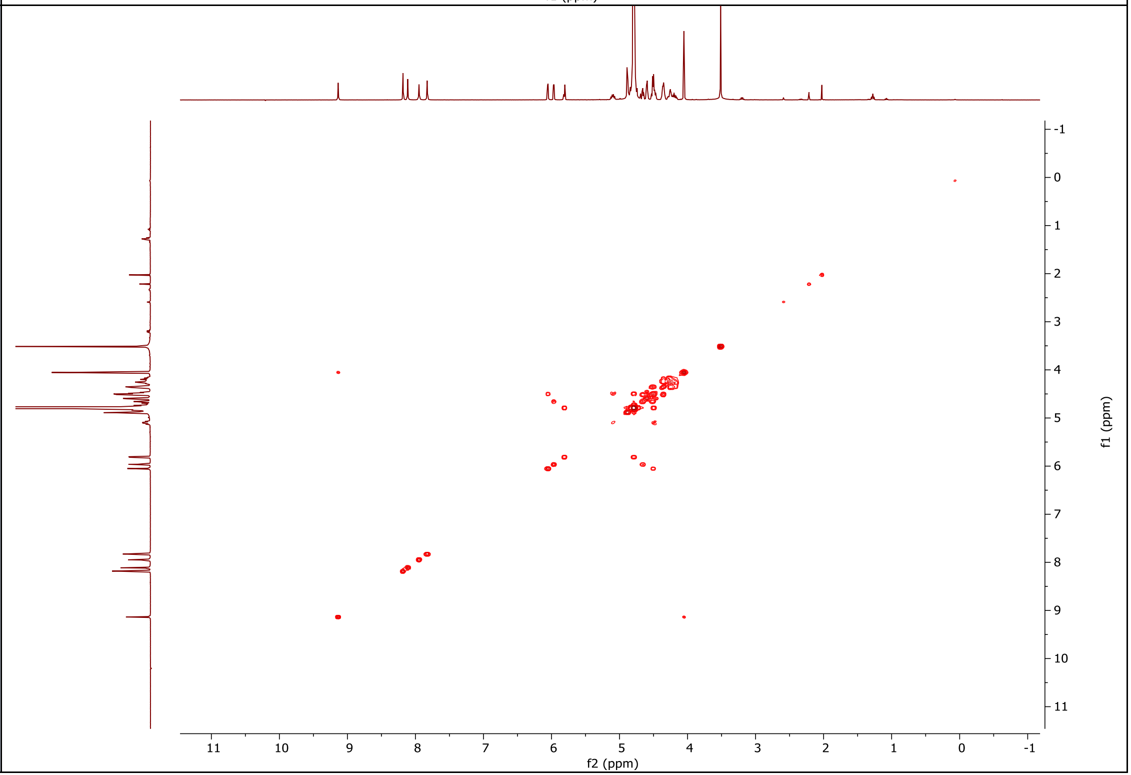

m^7Gppp-tr-C_2H_4pA_mpG 9

Structure

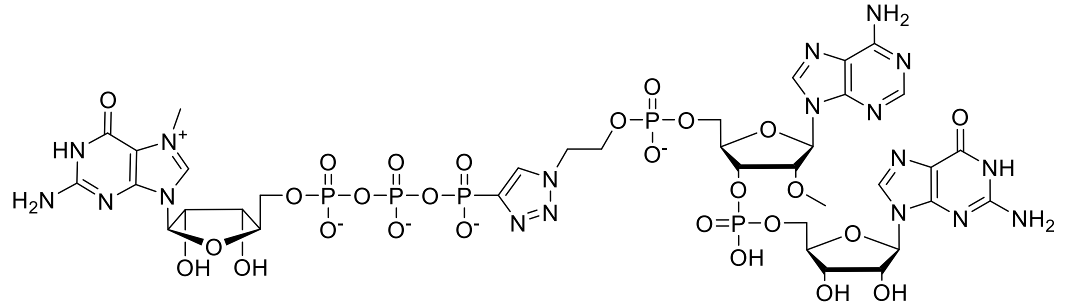

HPLC Profile

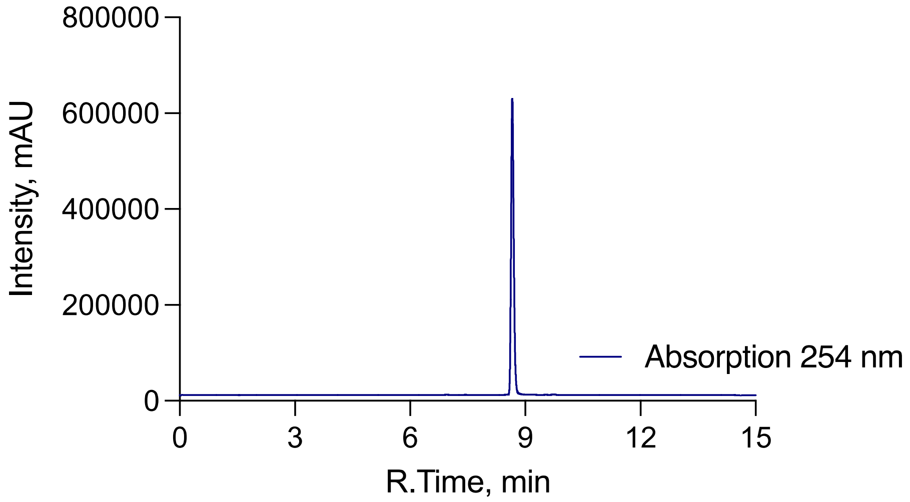

HR MS

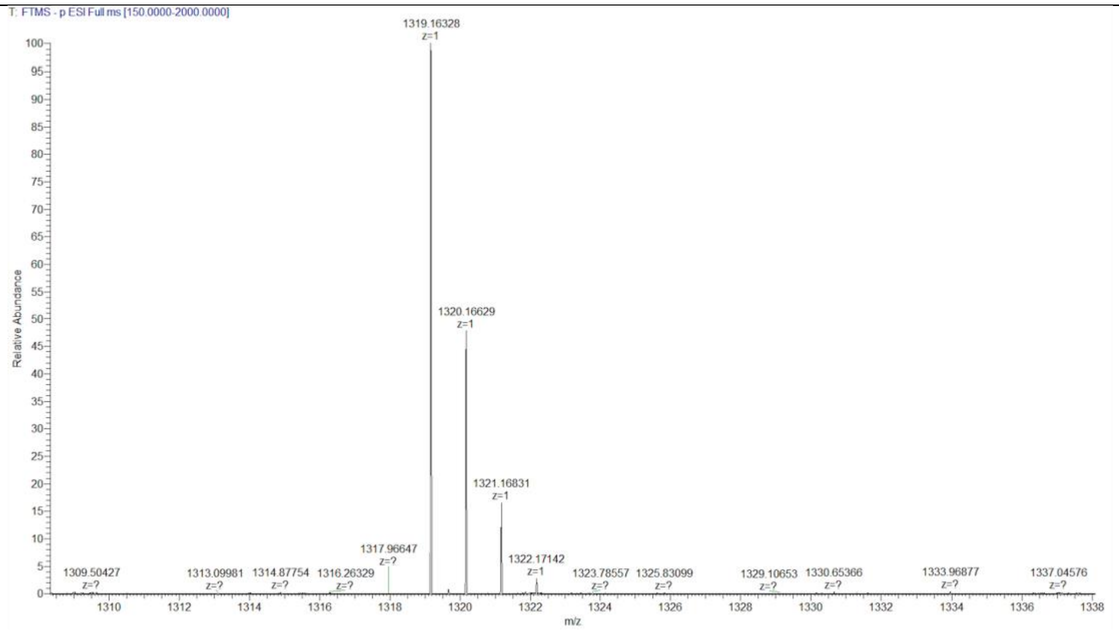

1H NMR

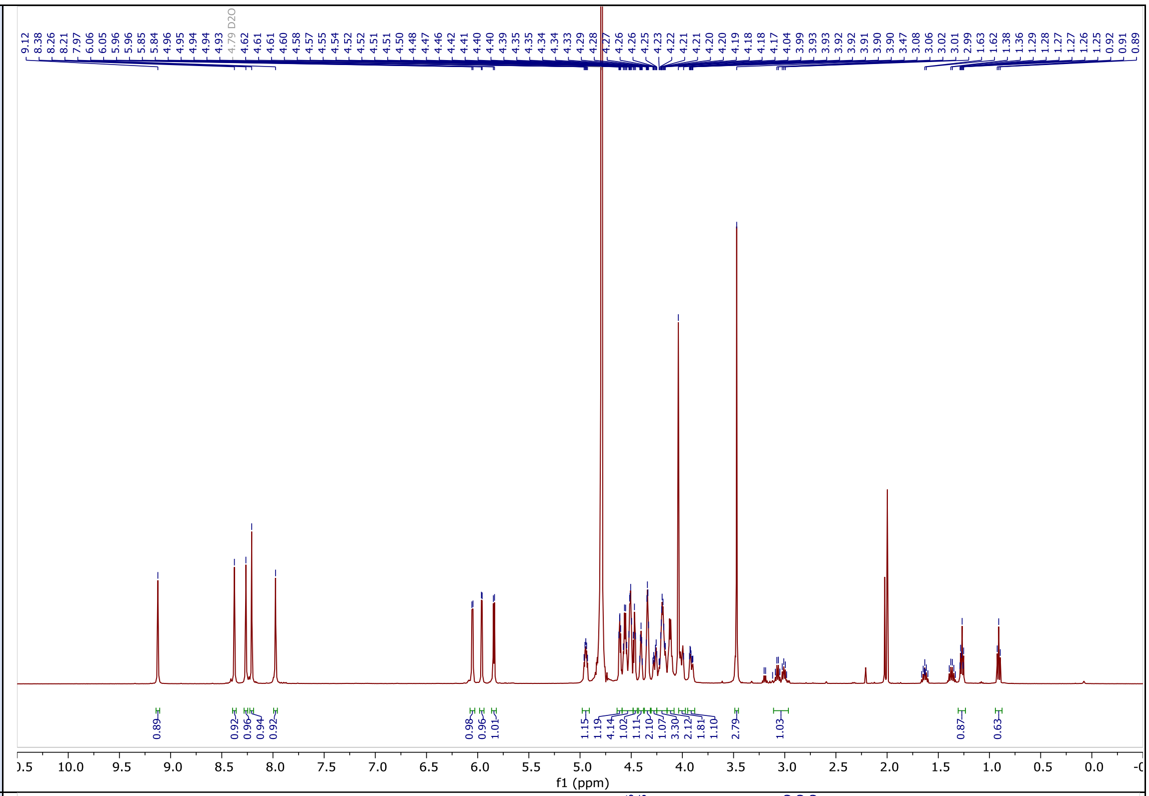

31P NMR

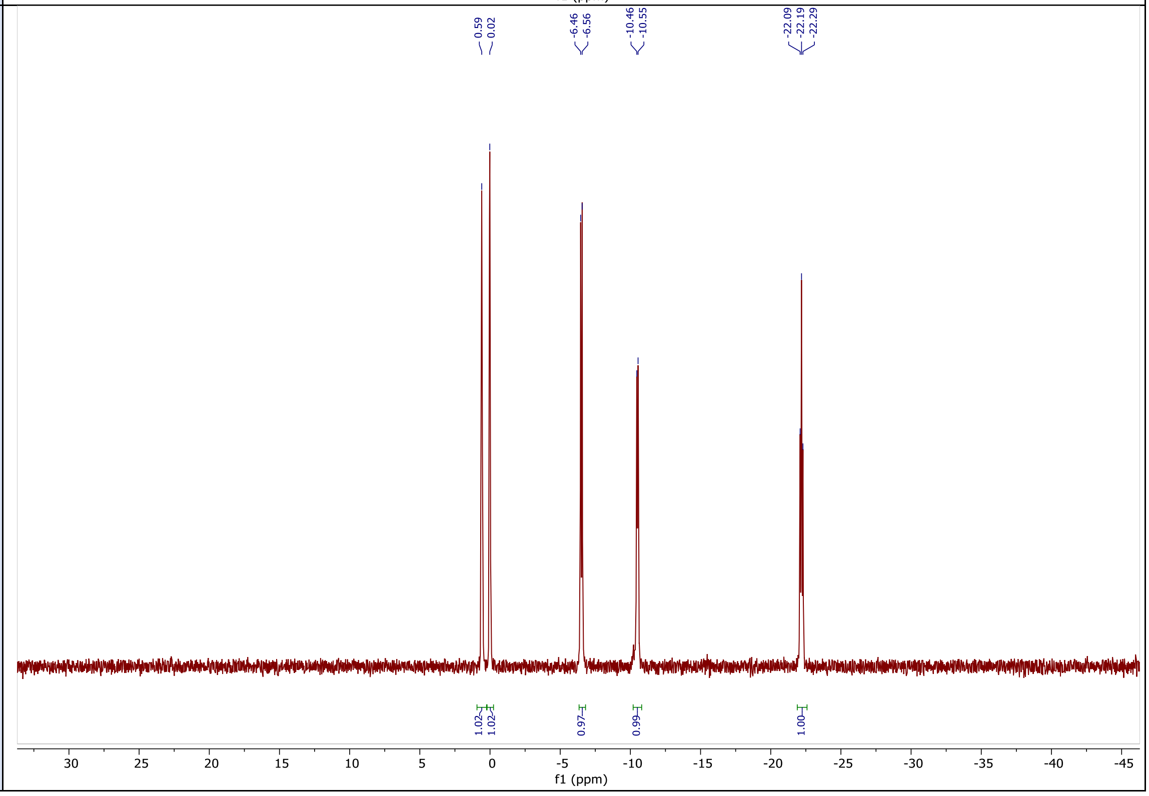

^{1}H-^{1}H COSY NMR

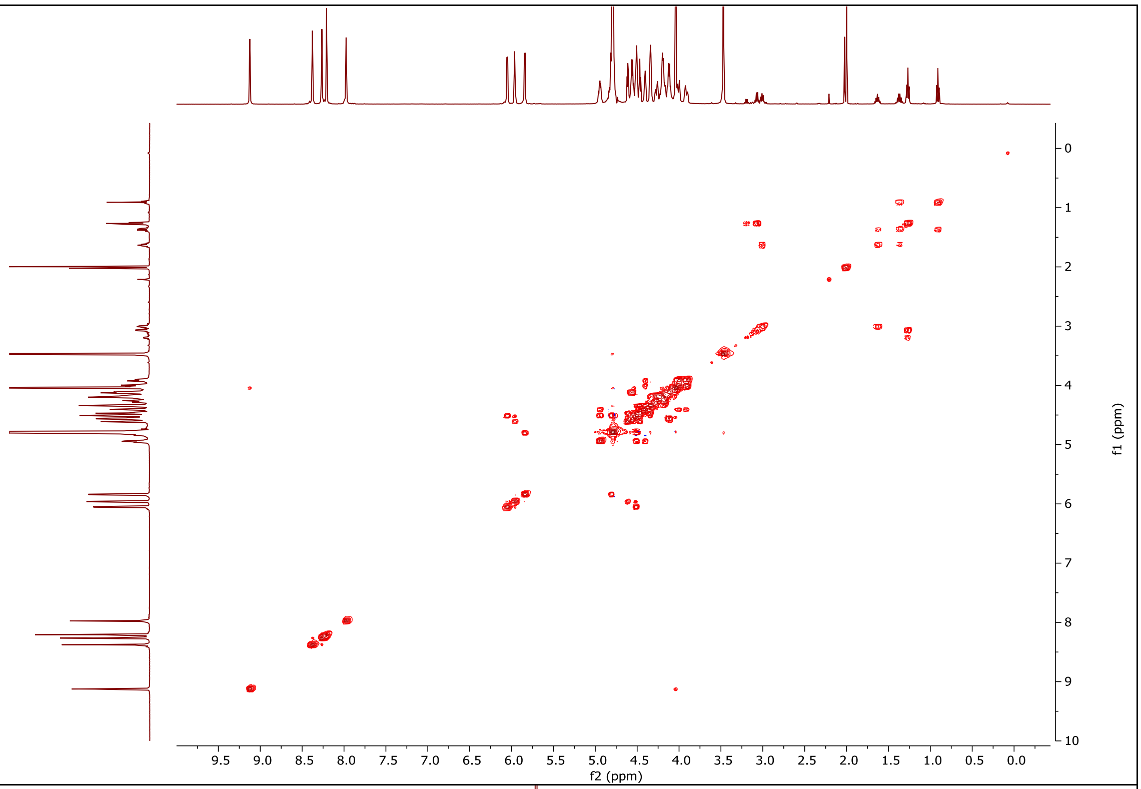

^{13}C-^{1}H HSQC NMR

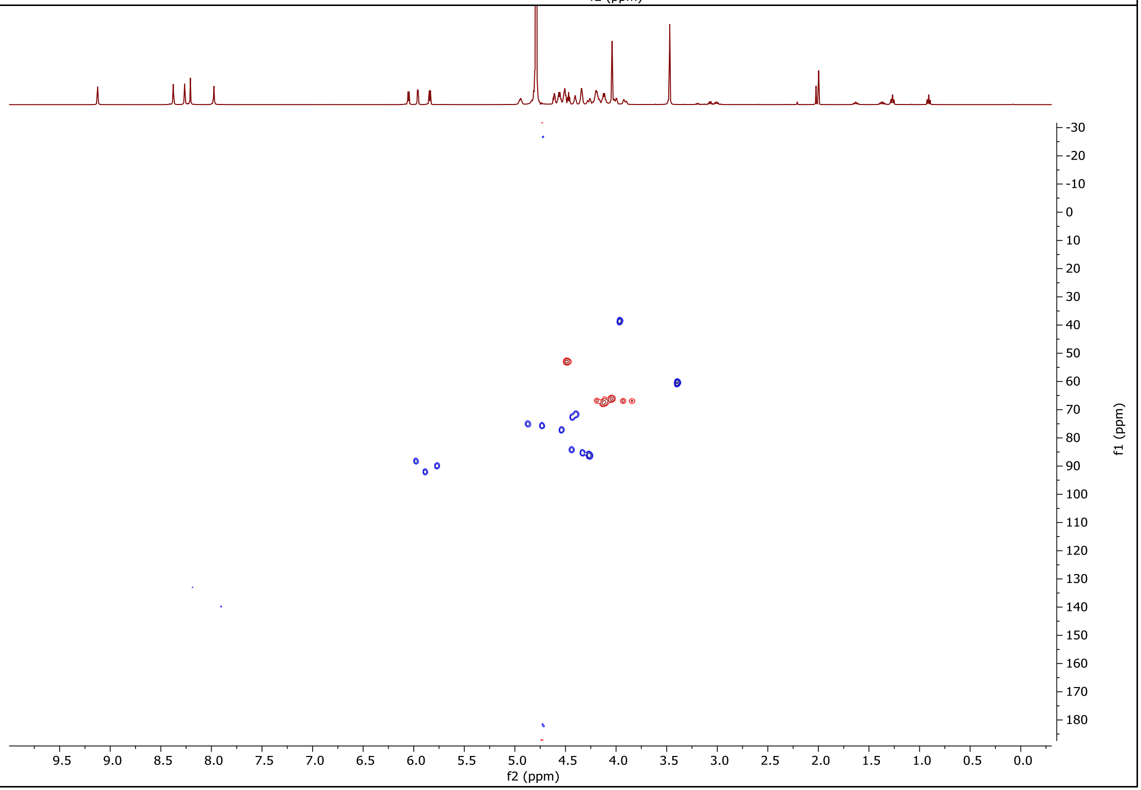

m⁷GpppG-carb-CH₂-tr-ApG (both isomers) 10a and 10b

Structure 10a	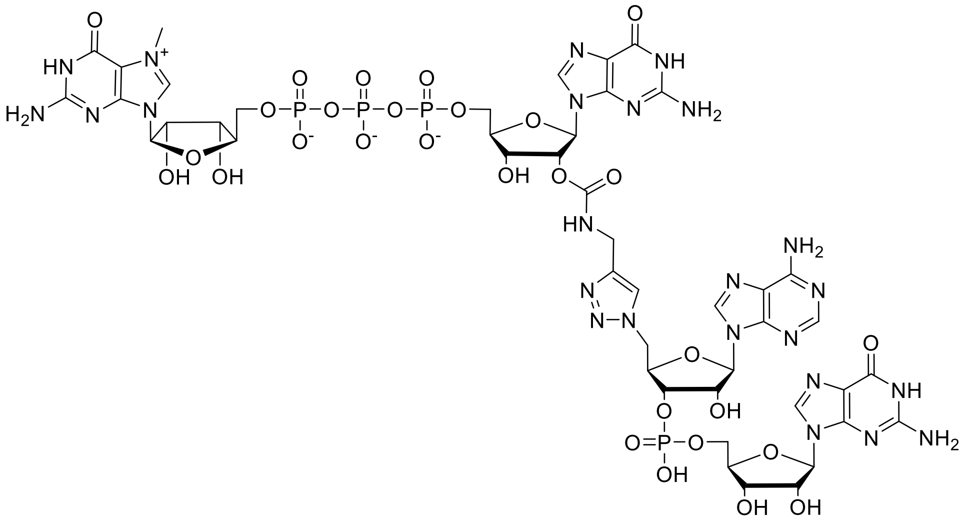
Structure 10b	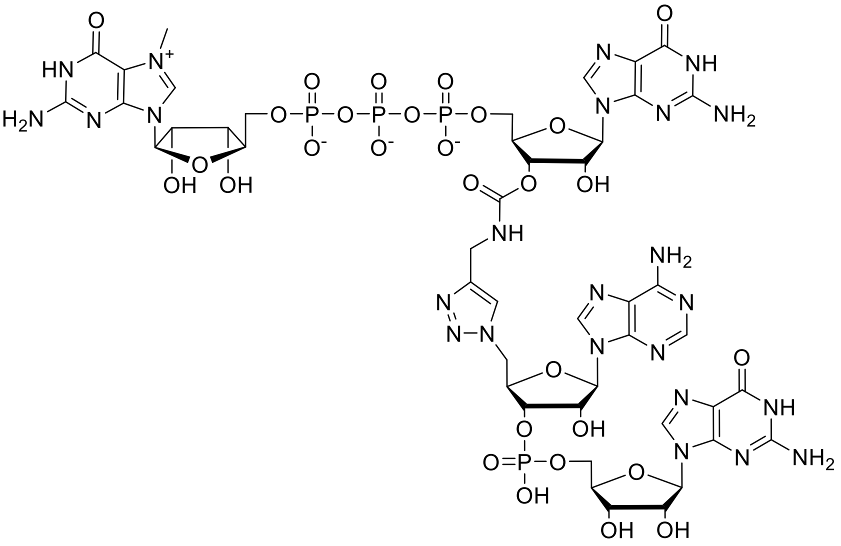
HPLC Profile (isomer 10a)	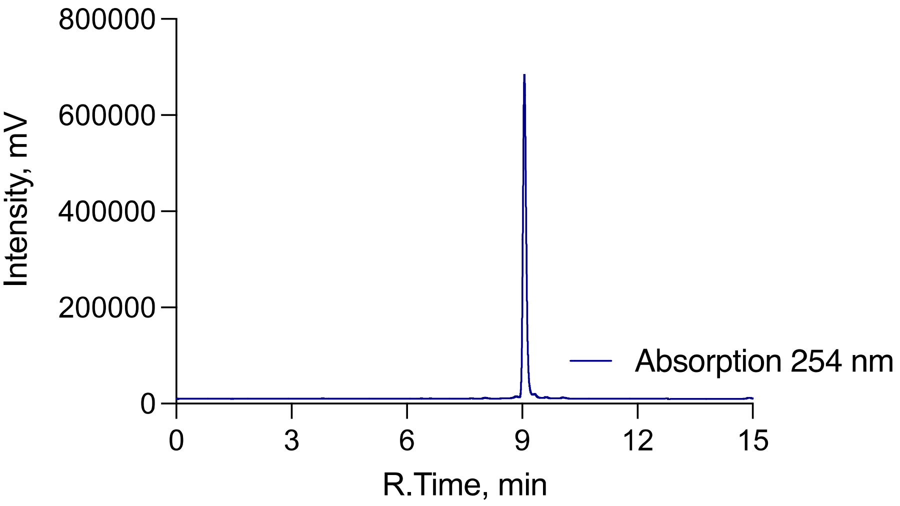

HPLC Profile (isomer 10b)	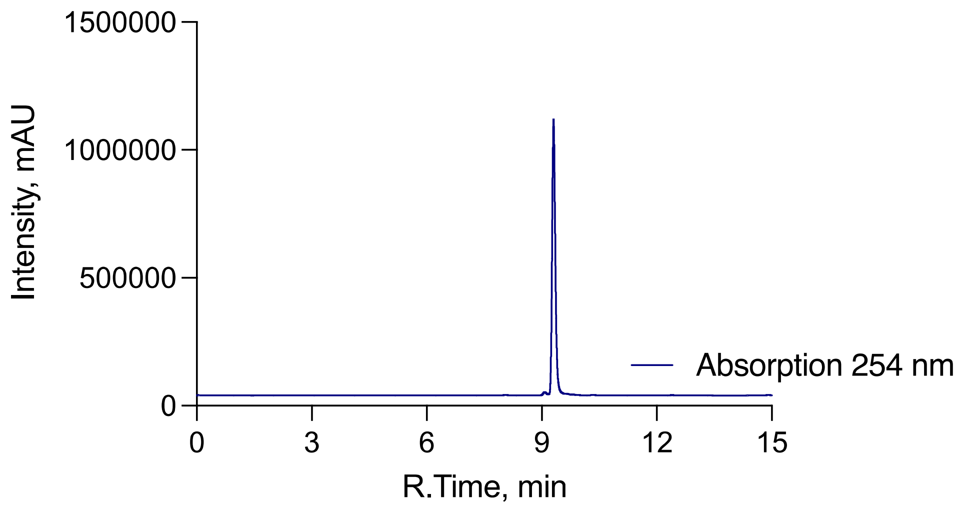
HR MS (isomer 10a)	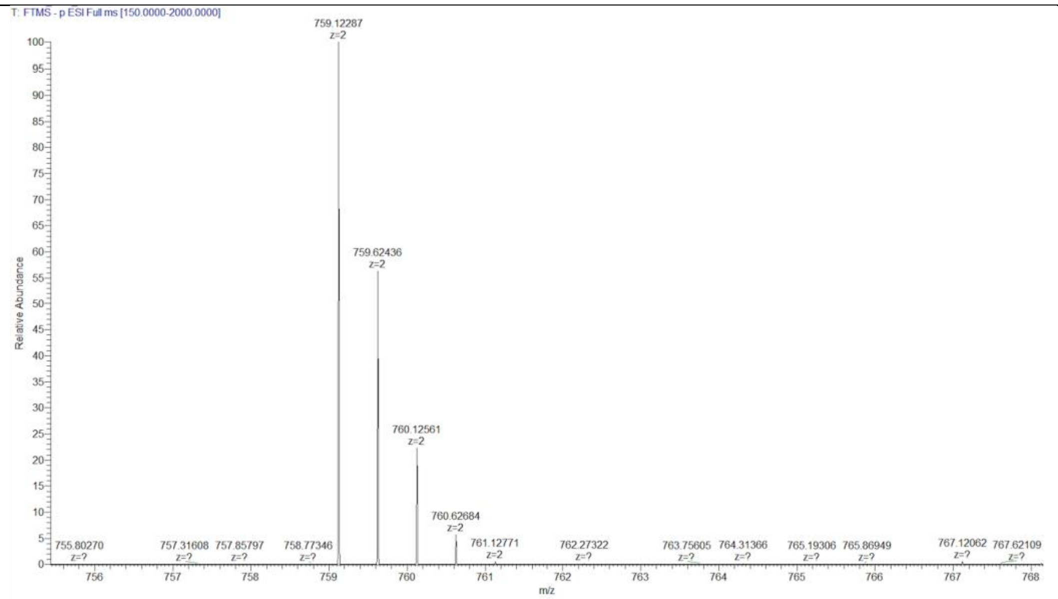
HR MS (isomer 10b)	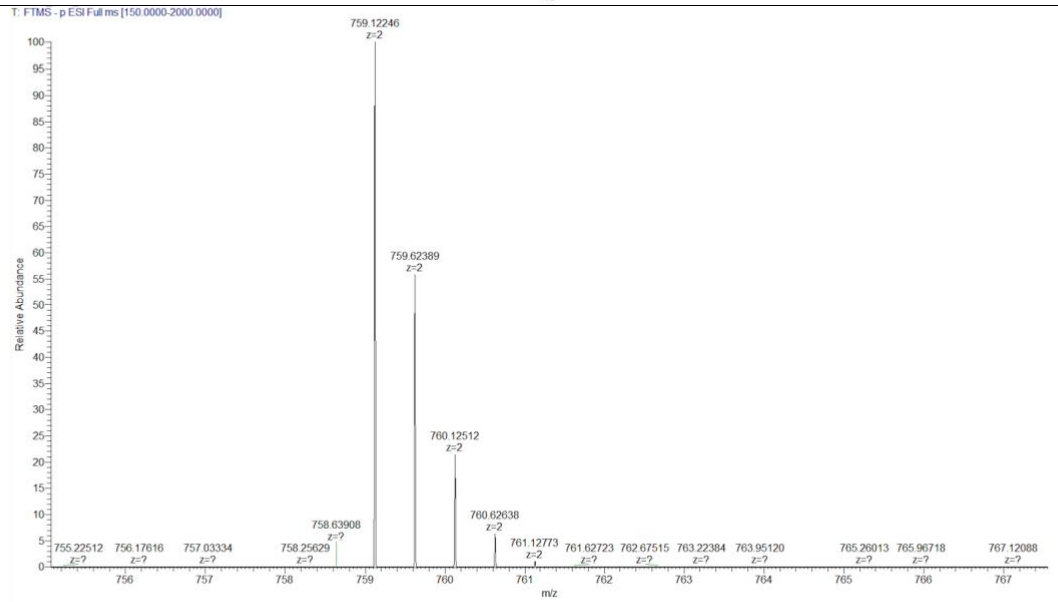

^1H NMR

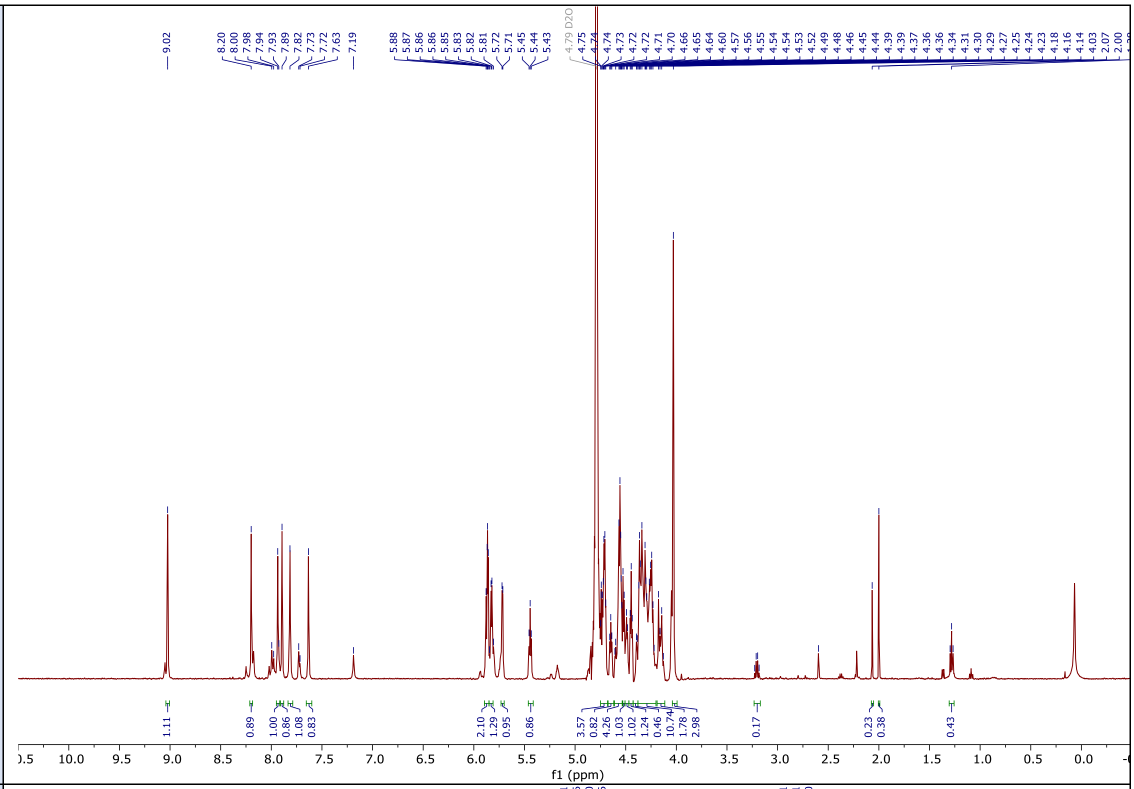

^{31}P NMR

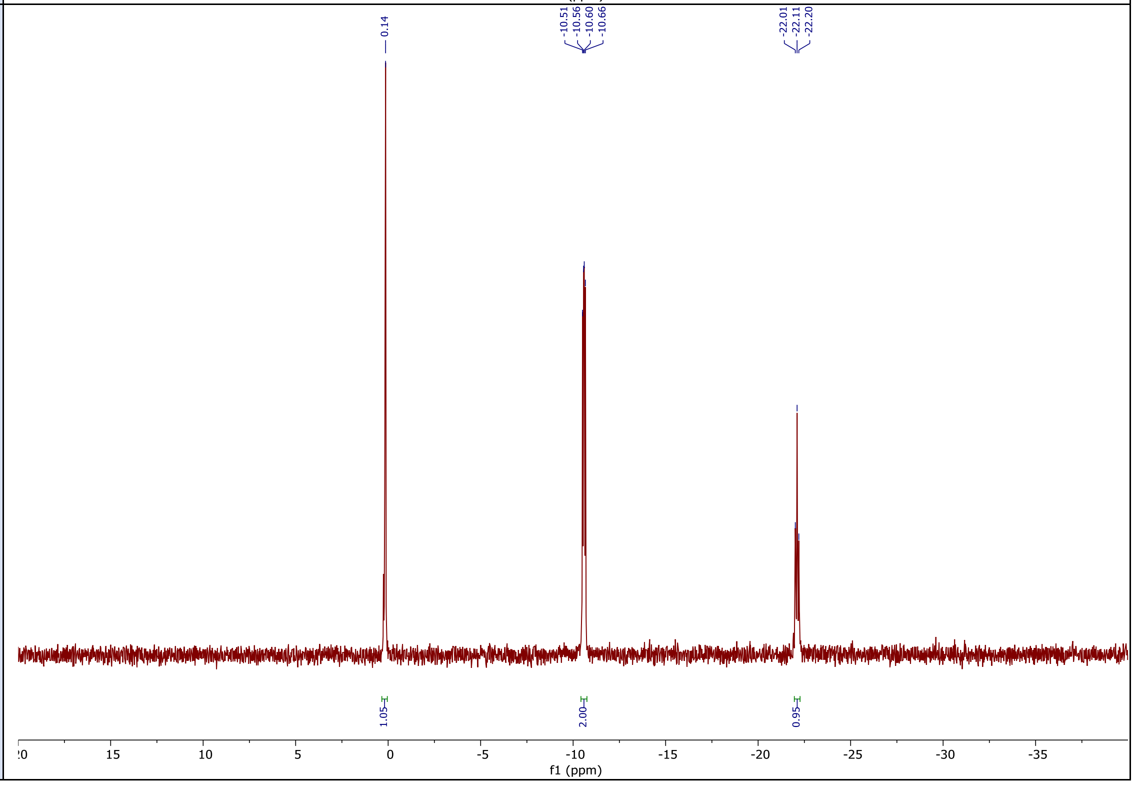

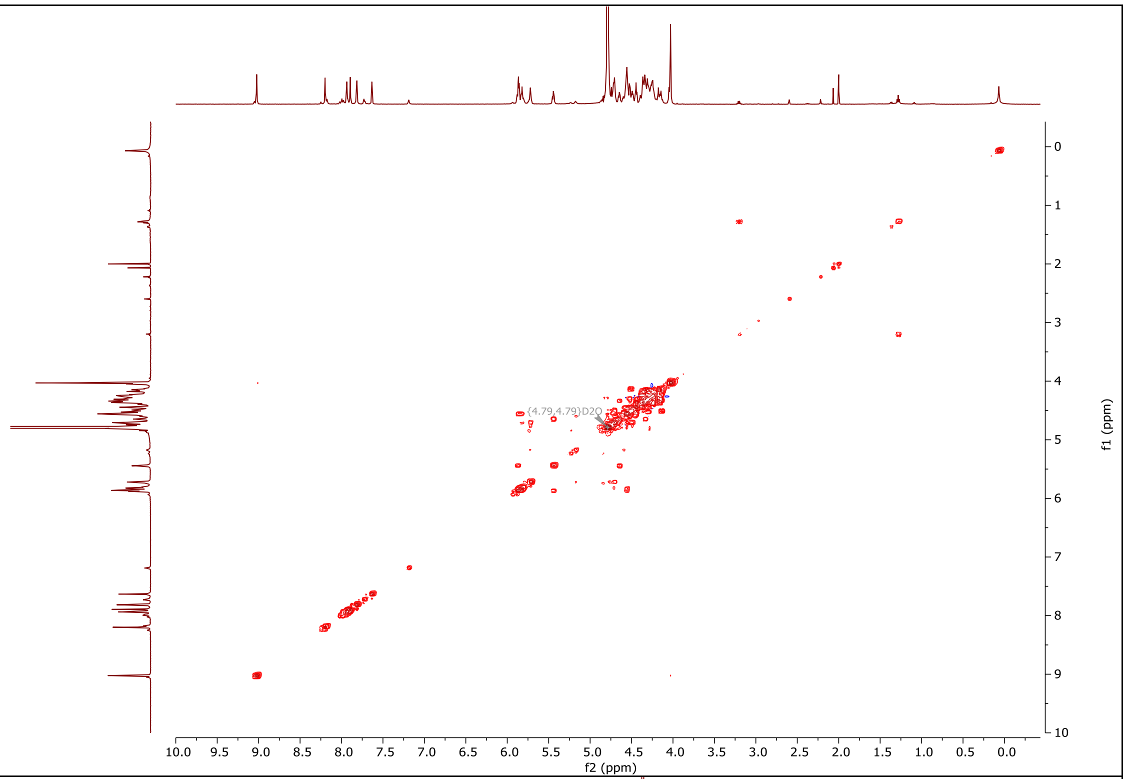

^{1}H-^{1}H COSY NMR

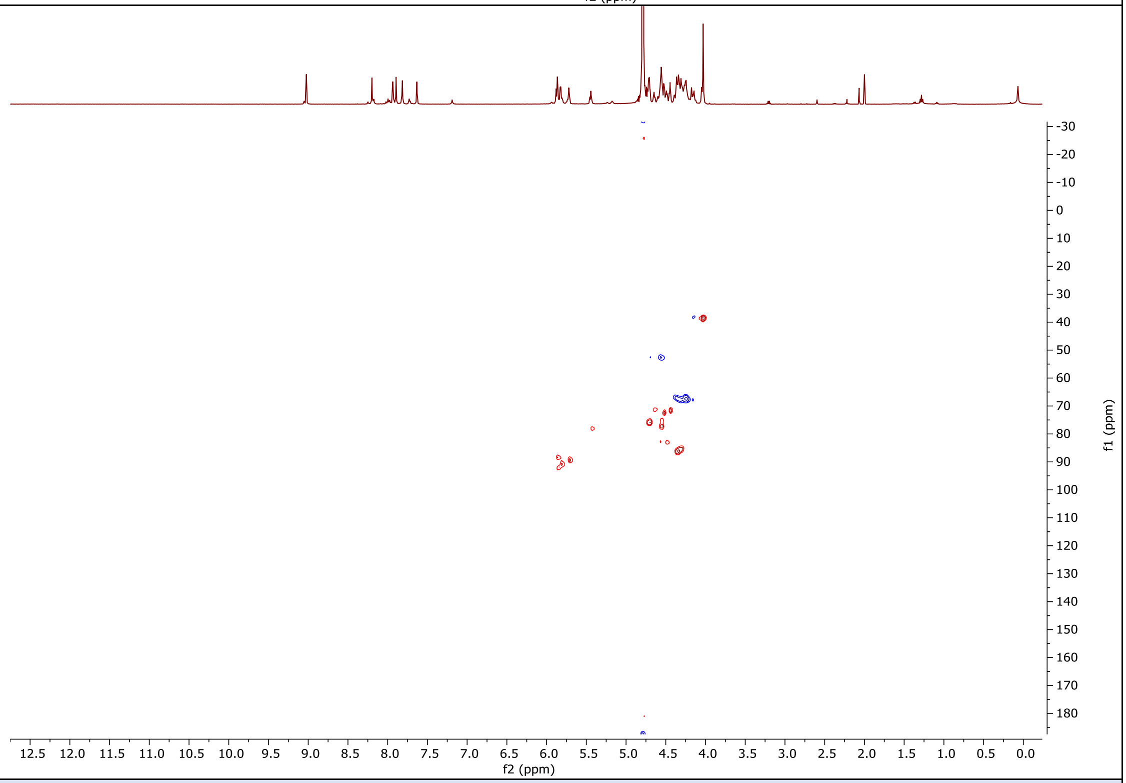

^{13}C-^{1}H HSQC

^{1}H NMR

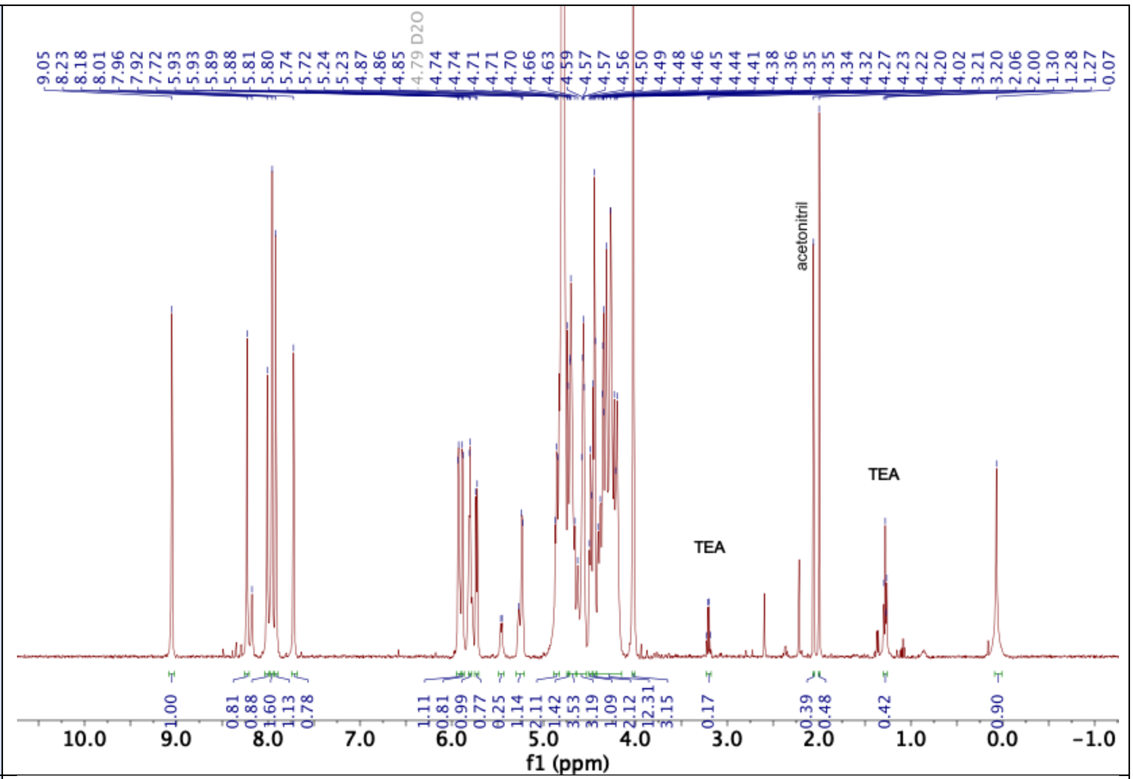

^{31}P NMR

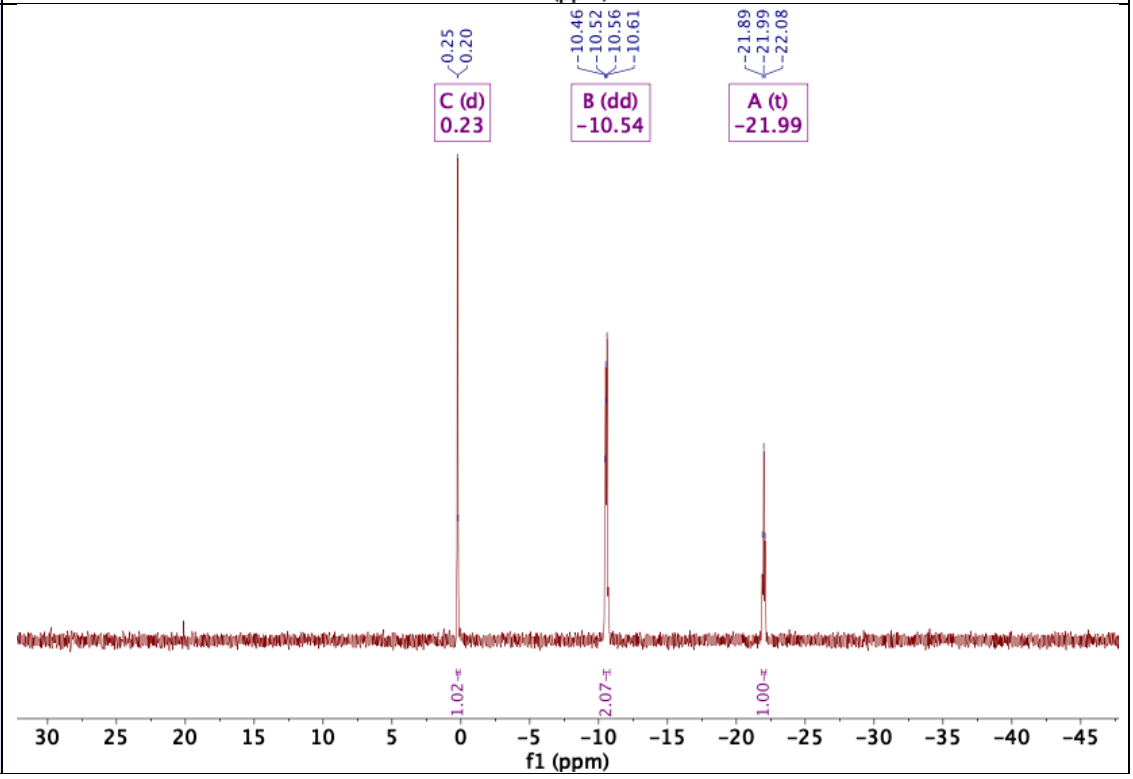

^1H-^1H COSY NMR

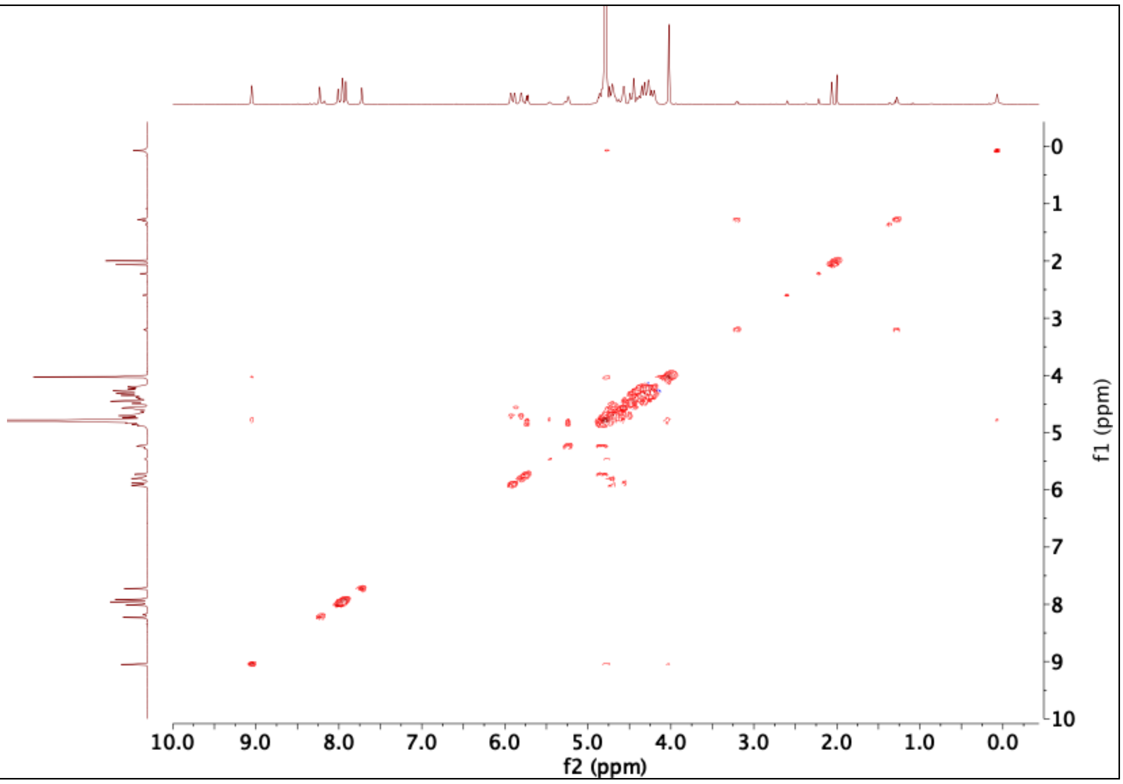

2'-OMe-3'-OC$_3$H$_3$-adenosine

Structure

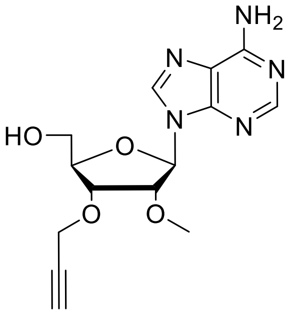

^{1}H NMR

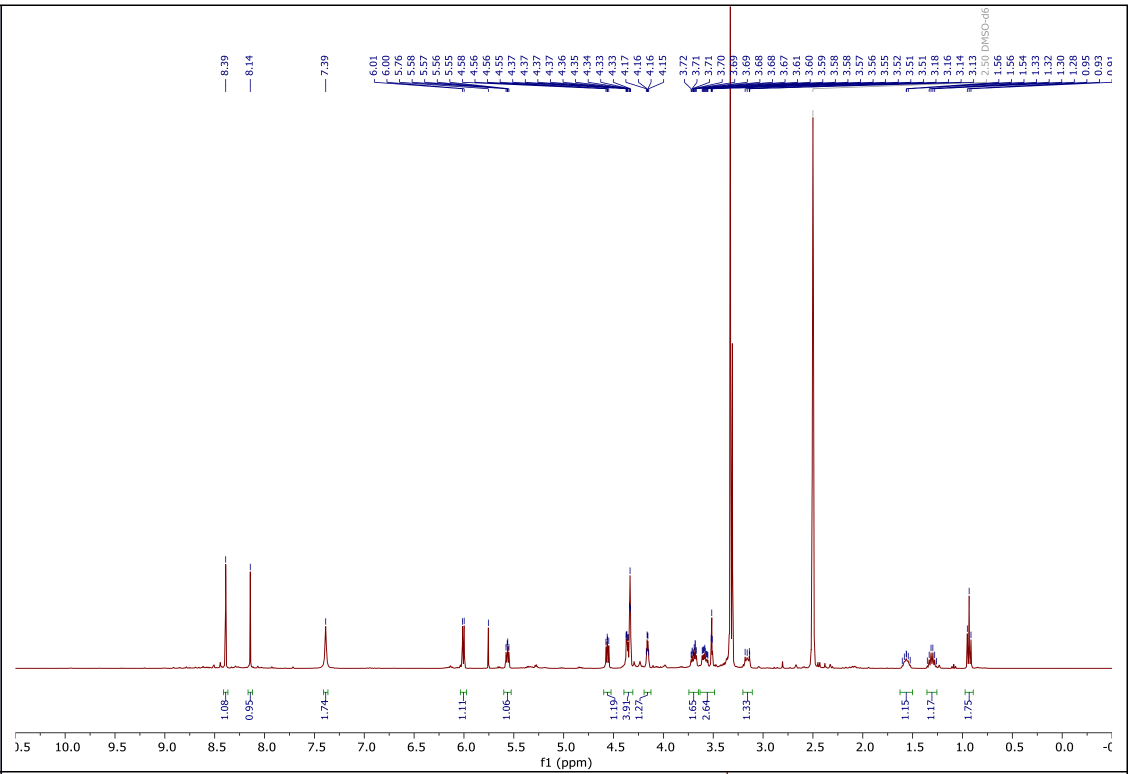

^{1}H-^{1}H COSY NMR

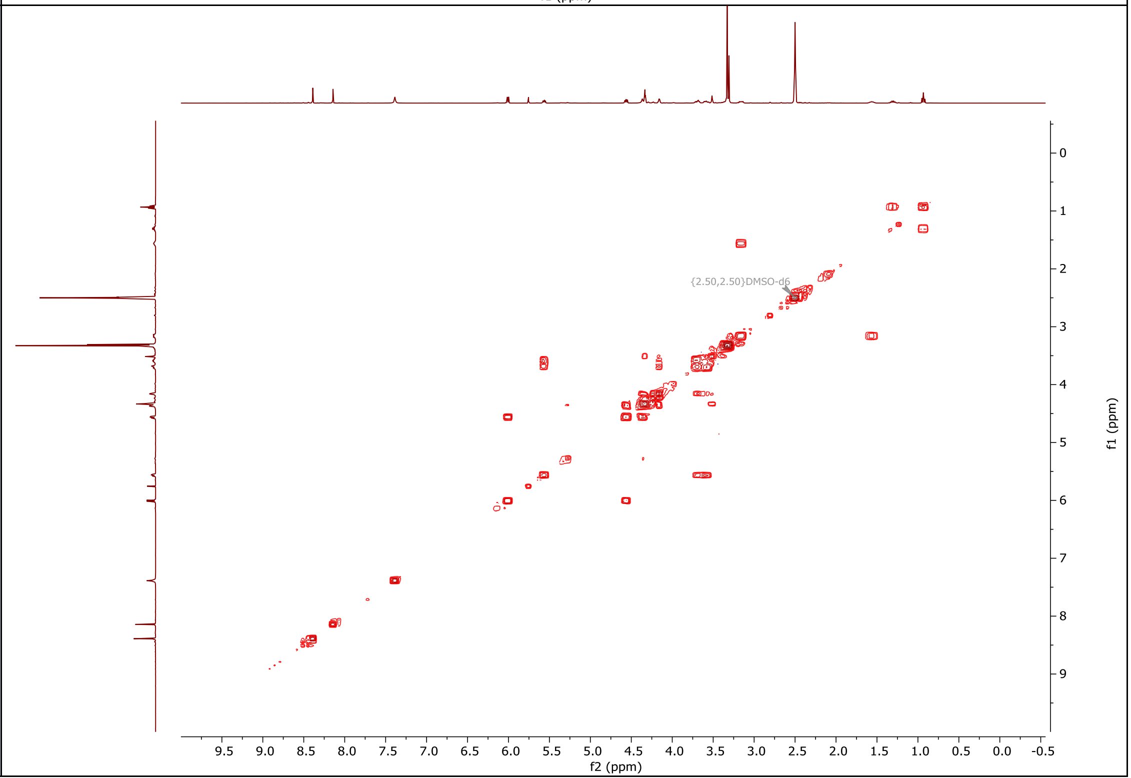

^{13}C-^{1}H HSQC

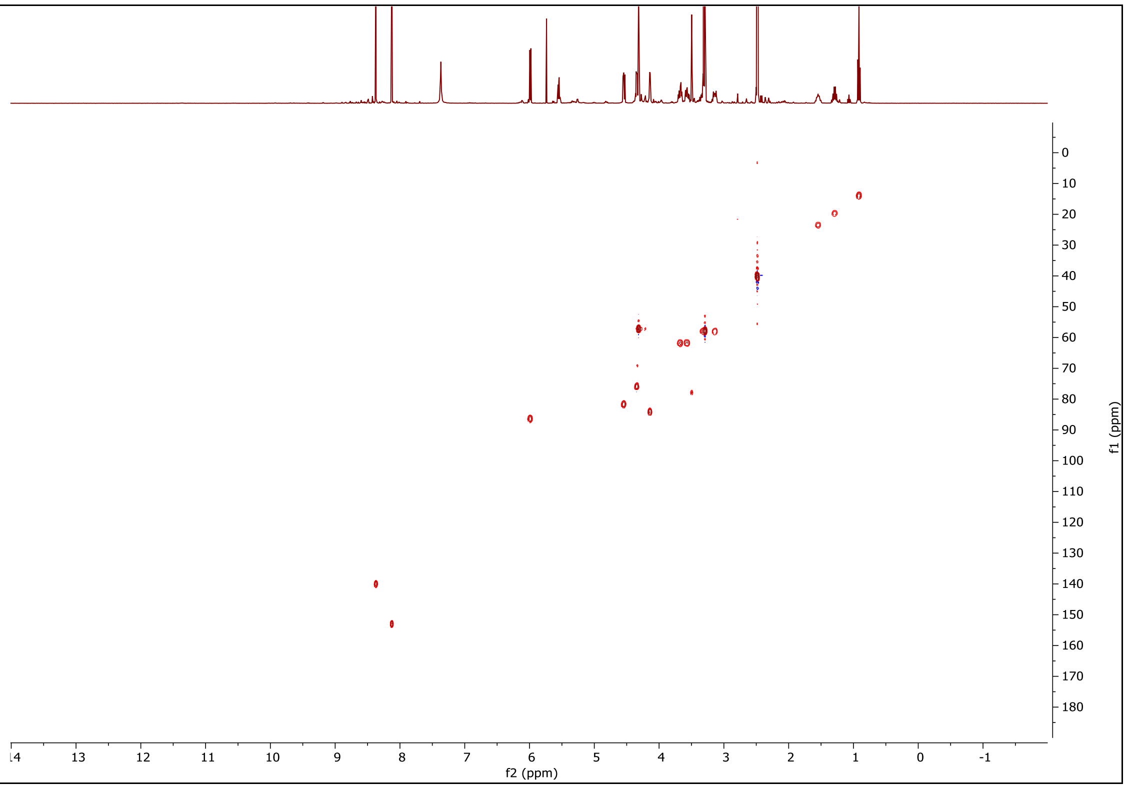

5'-monophoshapte 2'-OMe-3'-OC$_3$H$_3$ adenosine (2'-OMe-3'-OC$_3$H$_3$AMP)

Structure

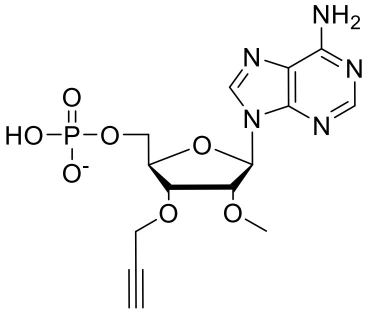

HPLC Profile

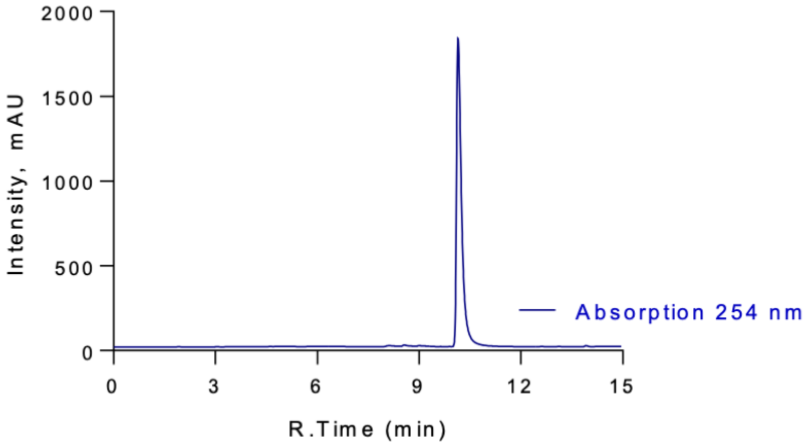

HR MS

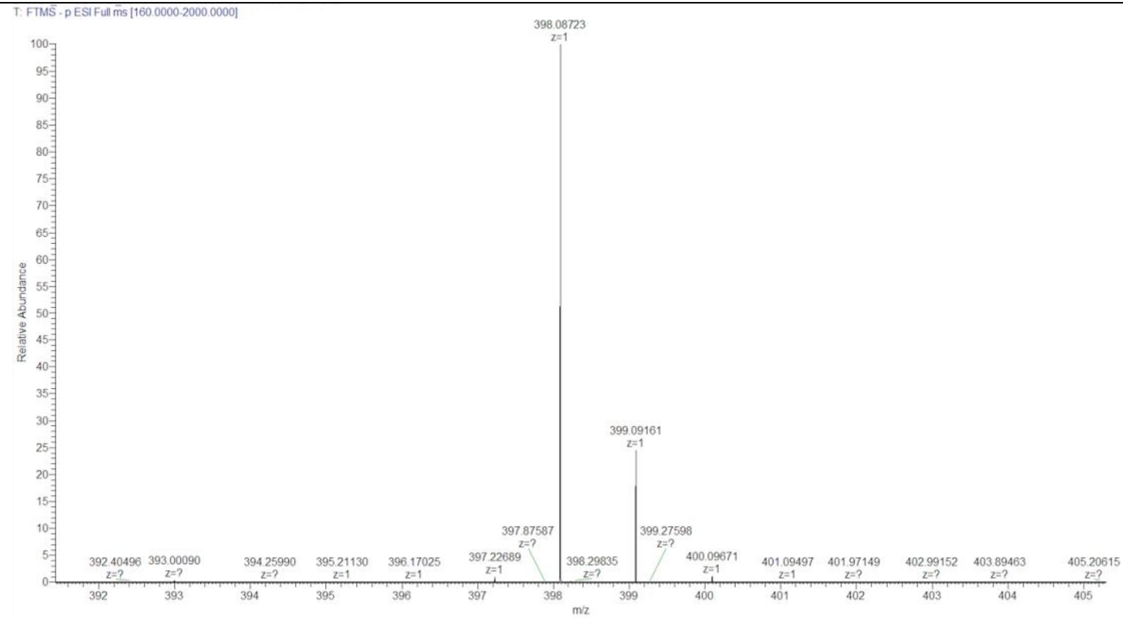

T: FTMS - p ESI Full ms [160.0000-2000.0000]
398.08723
z=1
399.09161
z=1
397.87587
z=?
399.27598
z=?
392.40496
z=?
393.00090
z=?
394.25990
z=?
395.21130
z=1
396.17025
z=1
397.22689
z=1
398.29835
z=?
400.09671
z=1
401.09497
z=1
401.97149
z=?
402.99152
z=?
403.89463
z=?
405.20615
z=?
Relative Abundance
m/z

^{1}H NMR

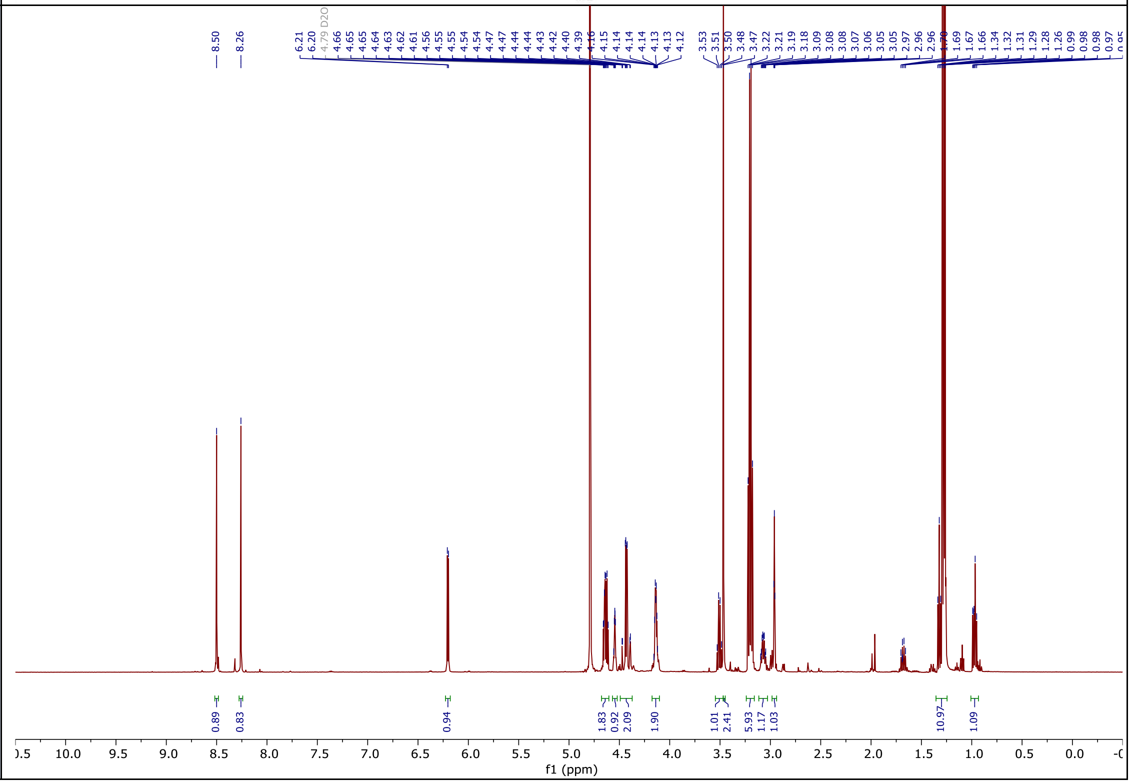

8.50
8.26
4.79 D2O
0.89
0.83
0.94
1.83
0.92
2.09
1.90
1.01
2.41
5.93
1.17
1.03
10.97
1.09
f1 (ppm)

^{31}P NMR

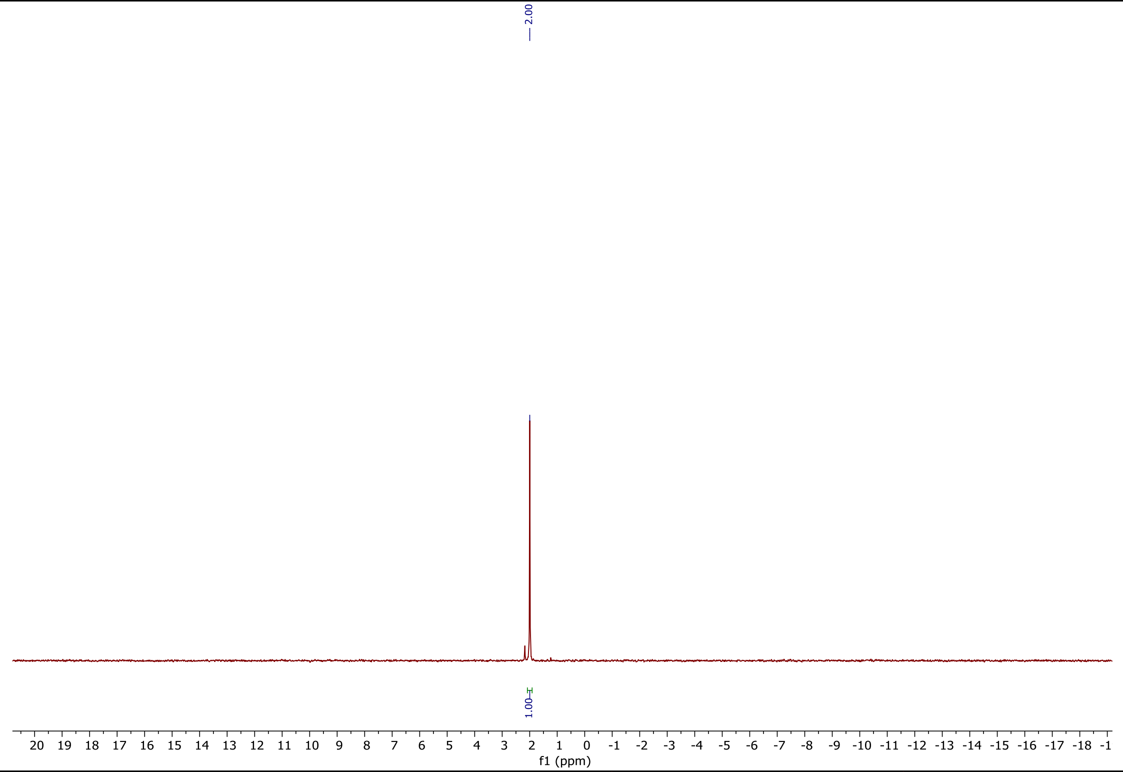

^{1}H-^{1}H COSY NMR

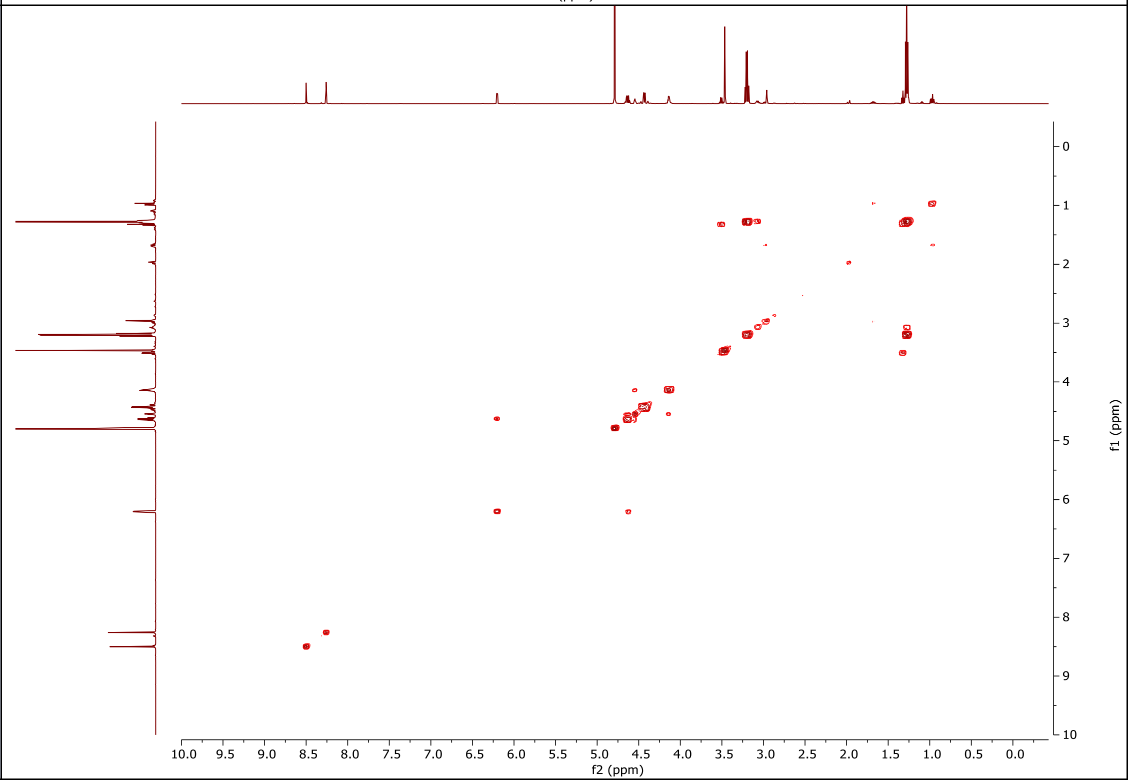

^{13}C-^{1}H HSQC

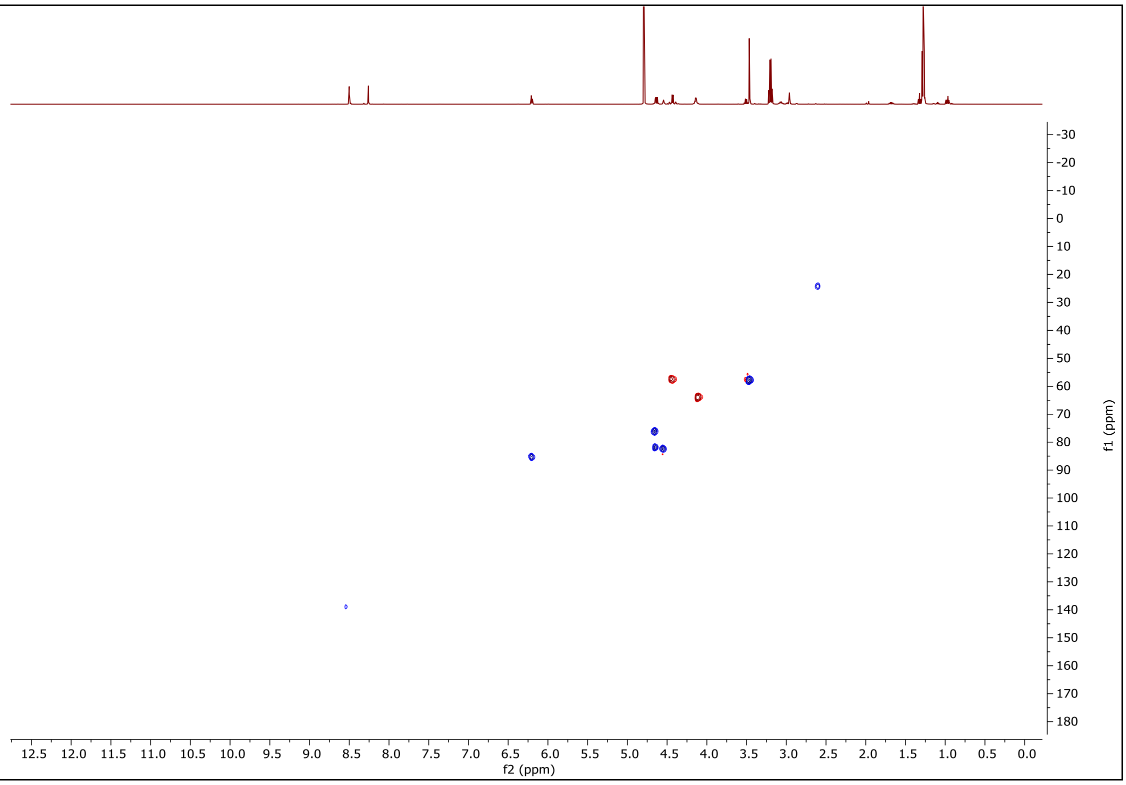

m^{7}GpppA-2'OMe-3'-OC$_3$H$_3$

Structure

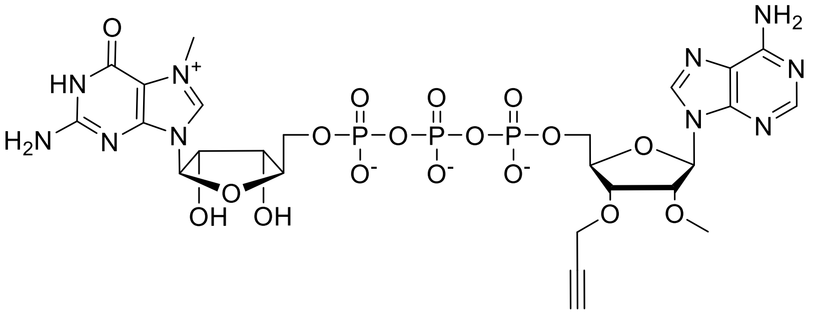

HPLC Profile

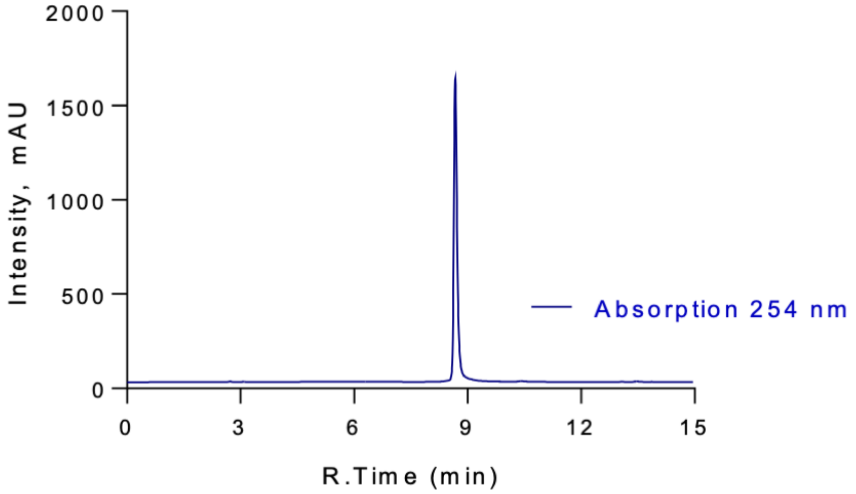

HR MS

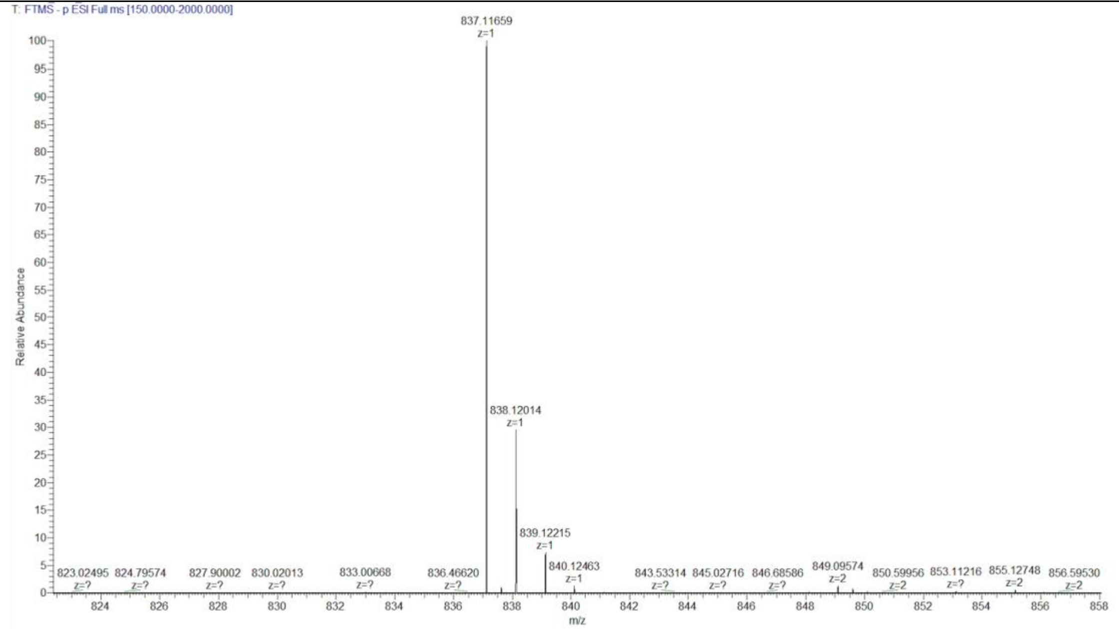

T: FTMS - p ESI Full ms [150.0000-2000.0000]
837.11659
z=1
838.12014
z=1
839.12215
z=1
840.12463
z=1
Relative Abundance
m/z

^{1}H NMR

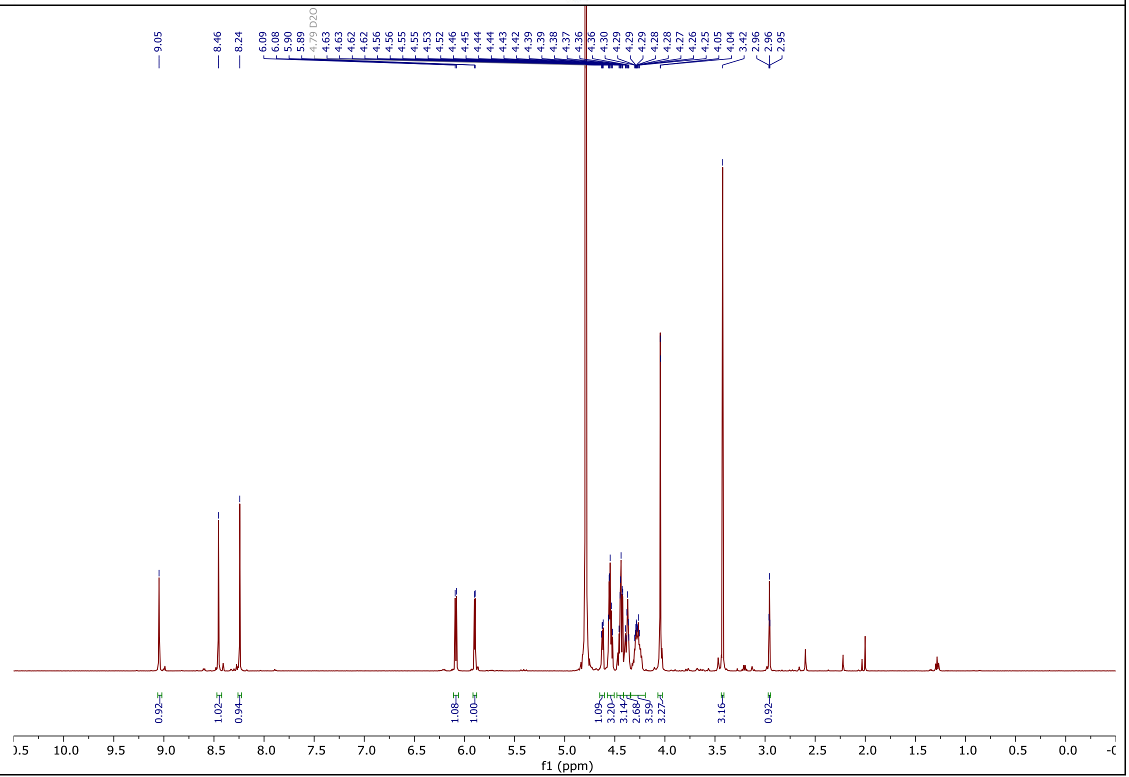

9.05
8.46
8.24
6.09
6.08
5.90
5.89
4.79 D2O
4.05
4.04
3.42
2.96
2.96
2.95
f1 (ppm)

^{31}P NMR

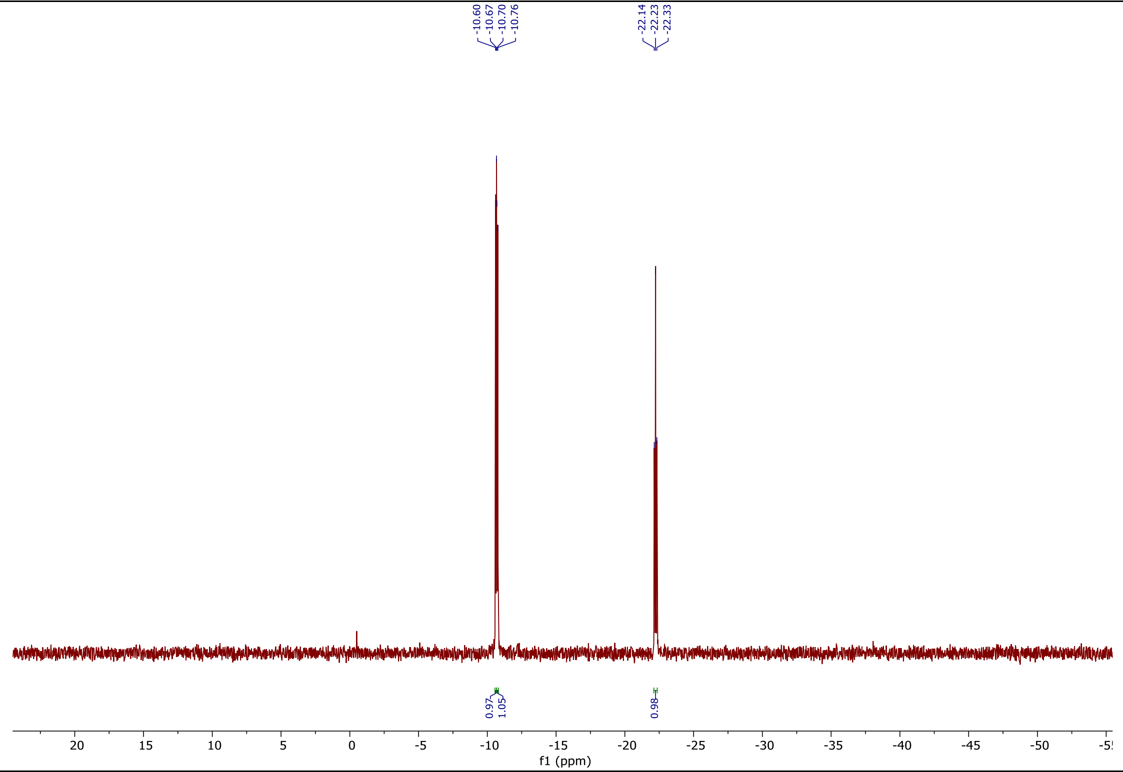

^{1}H-^{1}H COSY NMR

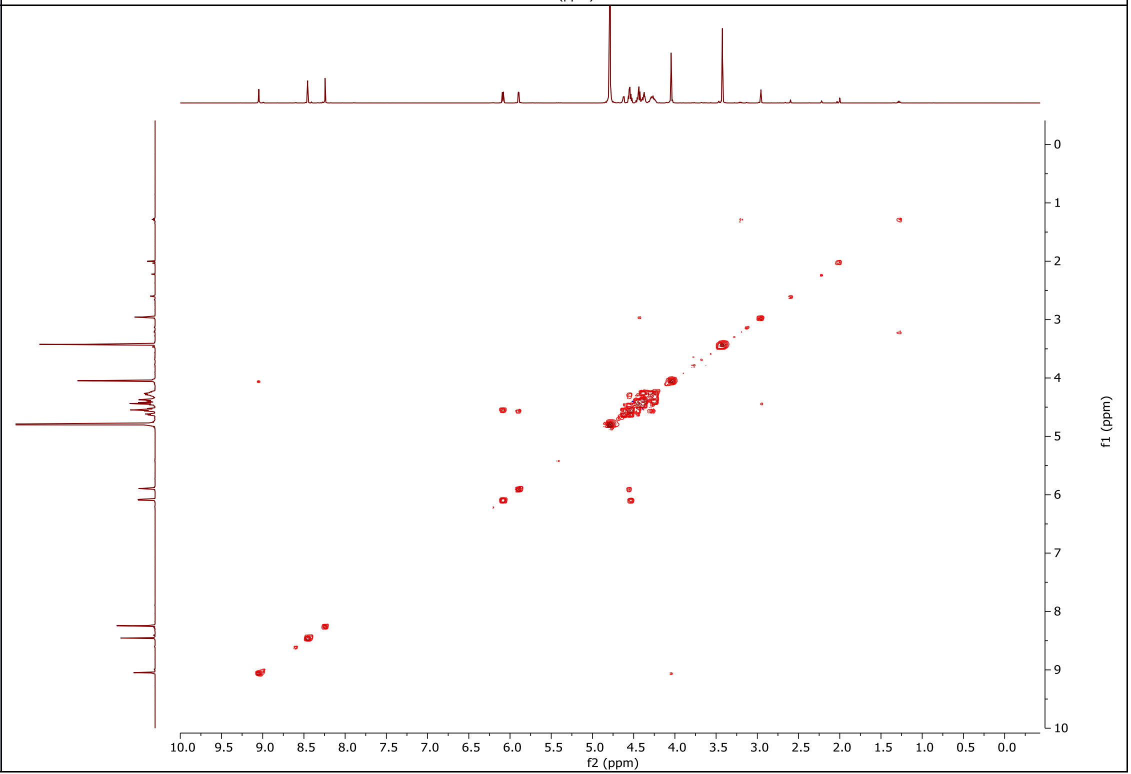

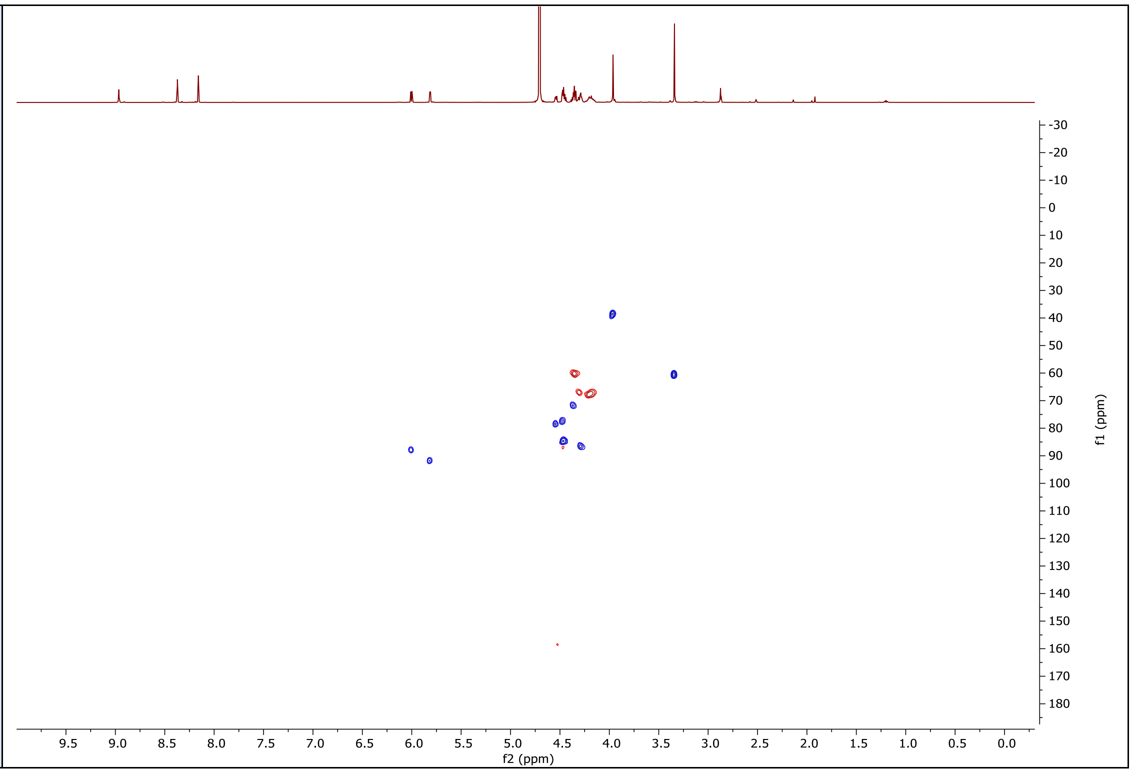

m^{7}GpppA$_m$-3'-OCH$_2$-tr-G 11

Structure

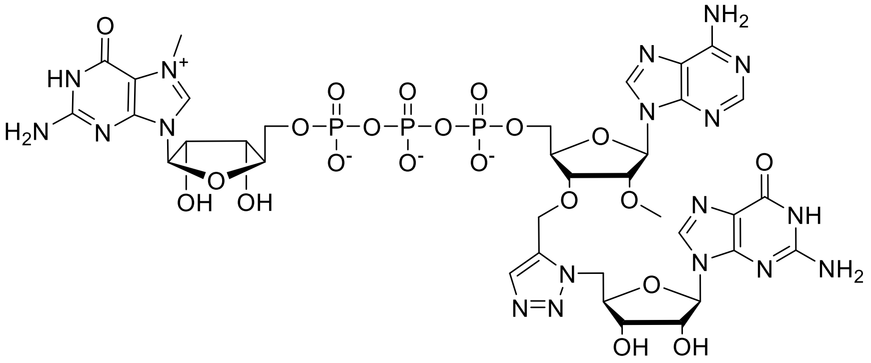

HPLC Profile

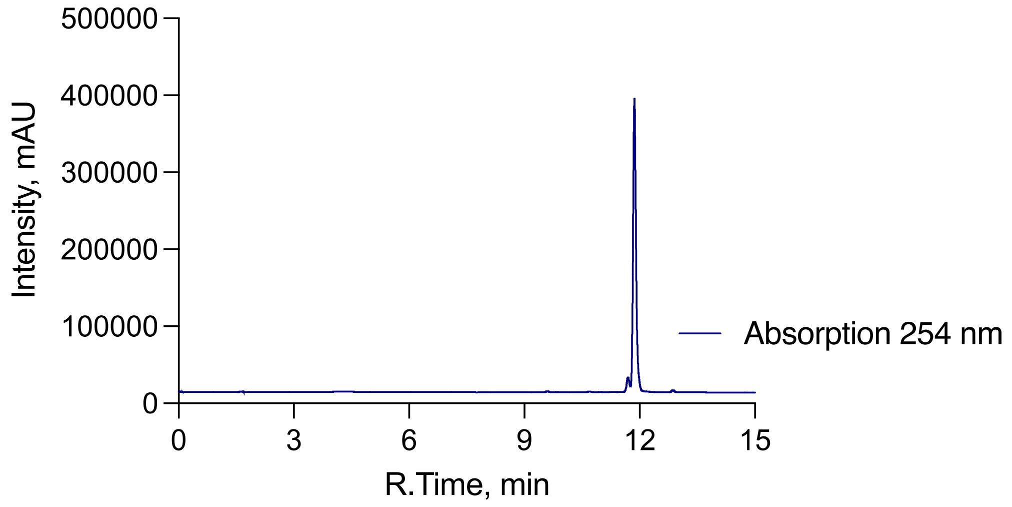

HR MS

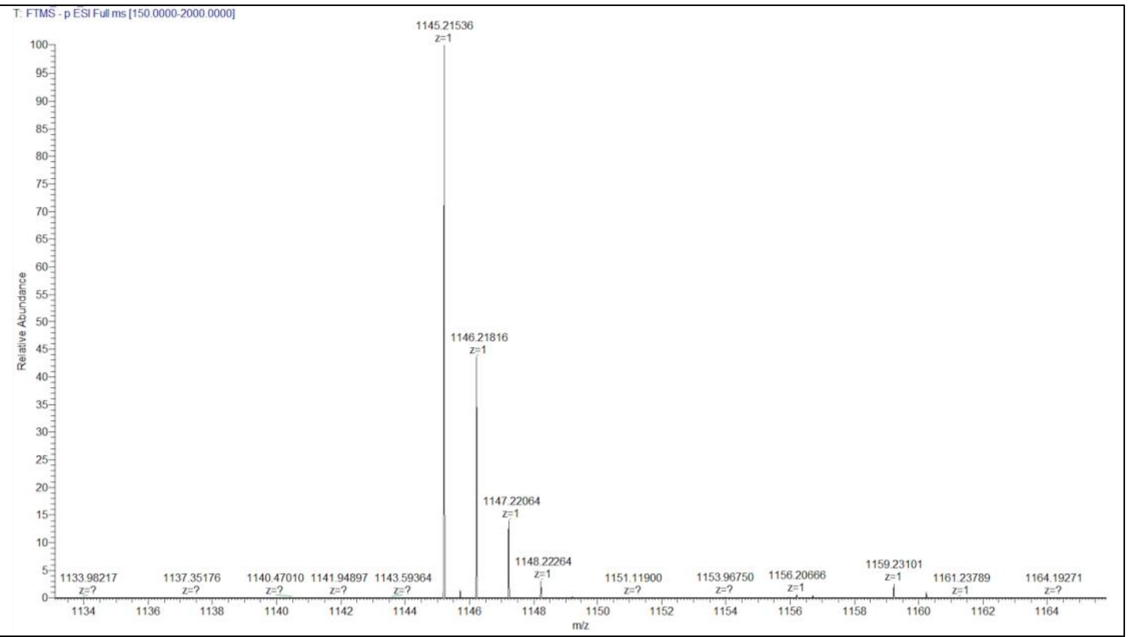

T: FTMS - p ESI Full ms [150.0000-2000.0000]
1145.21536
z=1
1146.21816
z=1
1147.22064
z=1
1148.22264
z=1
1133.98217
z=?
1137.35176
z=?
1140.47010
z=?
1141.94897
z=?
1143.59364
z=?
1151.11900
z=?
1153.96750
z=?
1156.20666
z=1
1159.23101
z=1
1161.23789
z=1
1164.19271
z=?
Relative Abundance
m/z

^{1}H NMR

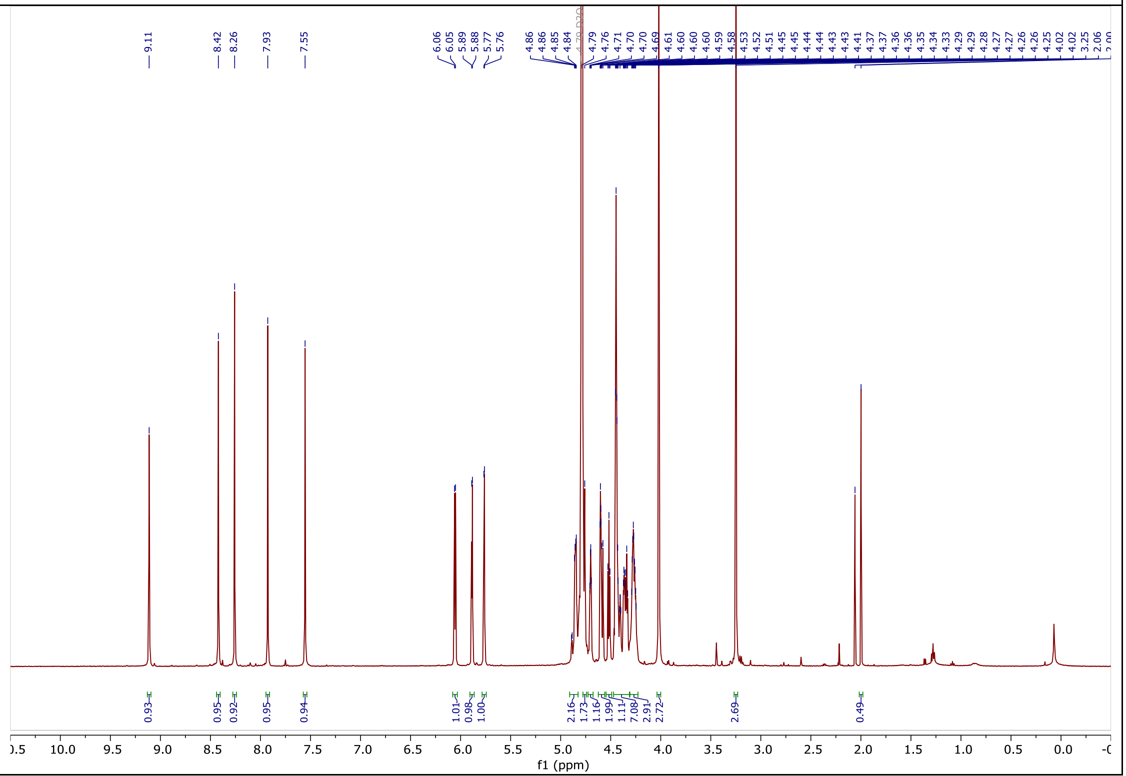

9.11
8.42
8.26
7.93
7.55
6.06
6.05
5.89
5.88
5.77
5.76
4.86
4.86
4.85
4.84
4.79
4.76
4.71
4.70
4.70
4.69
4.61
4.60
4.60
4.60
4.59
4.58
4.53
4.52
4.51
4.45
4.45
4.44
4.43
4.43
4.41
4.37
4.37
4.36
4.36
4.35
4.34
4.33
4.29
4.28
4.27
4.27
4.26
4.26
4.25
4.02
4.02
3.25
2.06
0.93
0.95
0.92
0.95
0.94
1.01
0.98
1.00
2.16
1.73
1.16
1.99
1.11
7.08
2.91
2.72
2.69
0.49
f1 (ppm)

^{31}P NMR

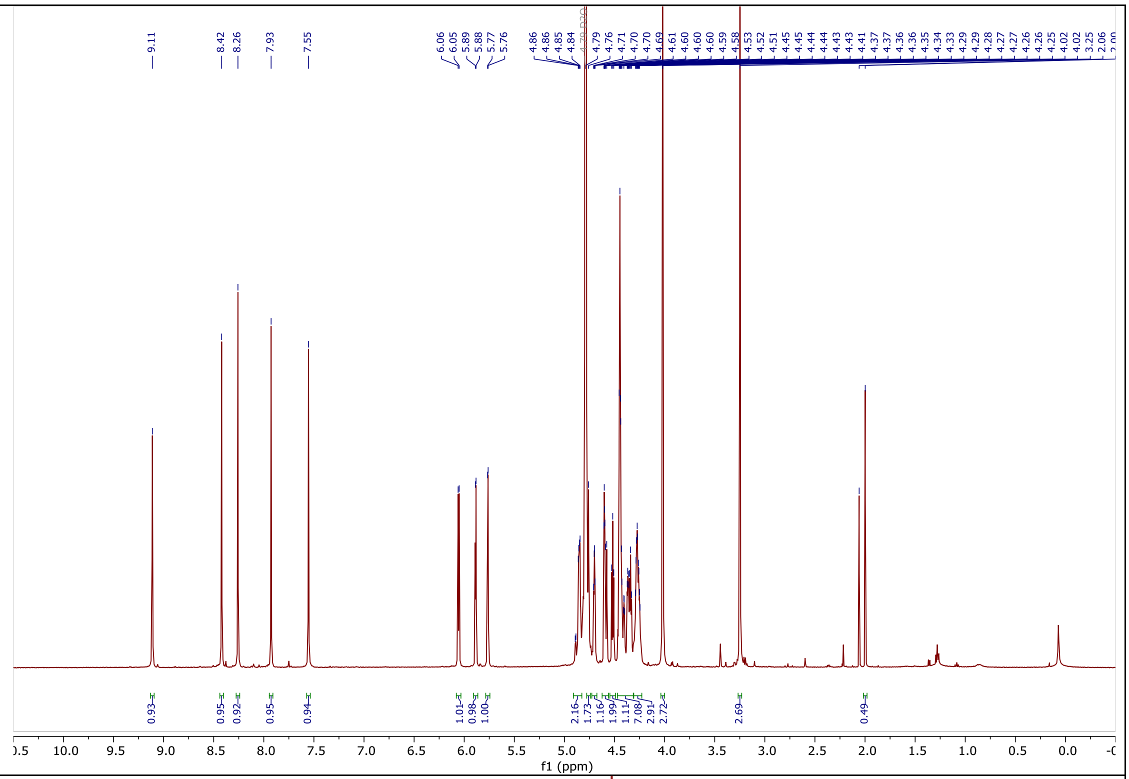

^{1}H-^{1}H COSY NMR

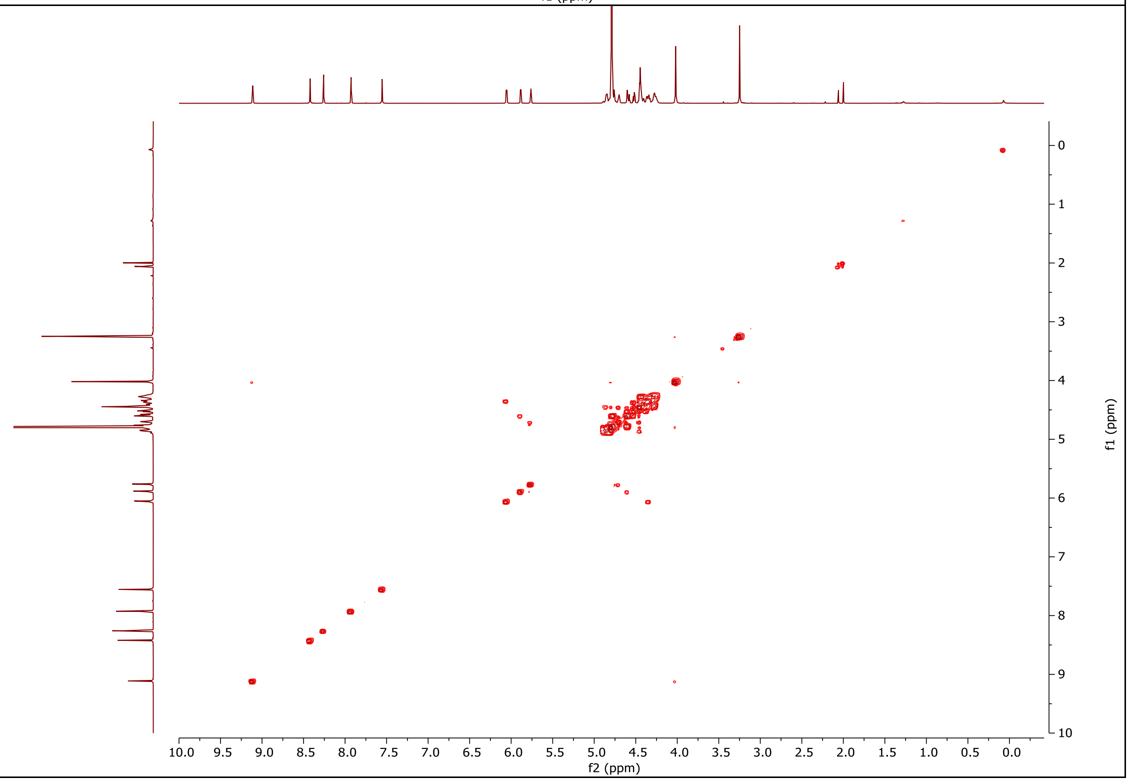

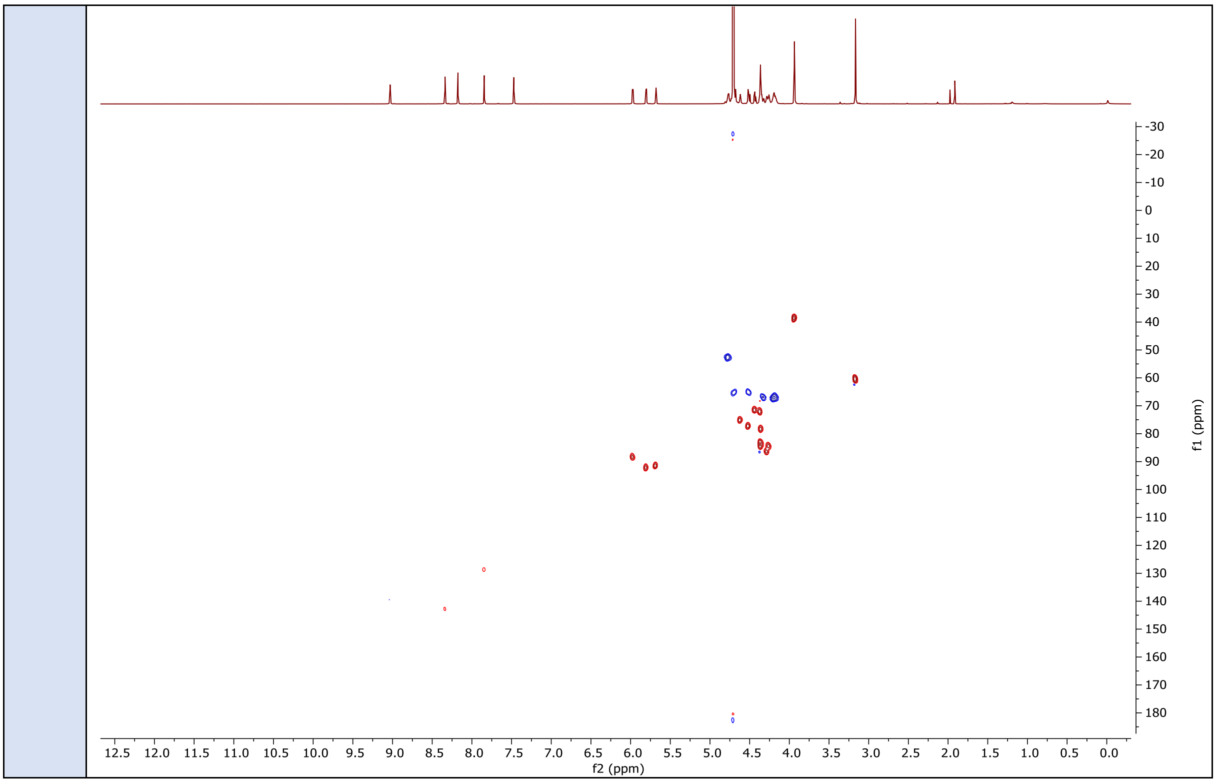

Figures

Figure S1. mRNA purity

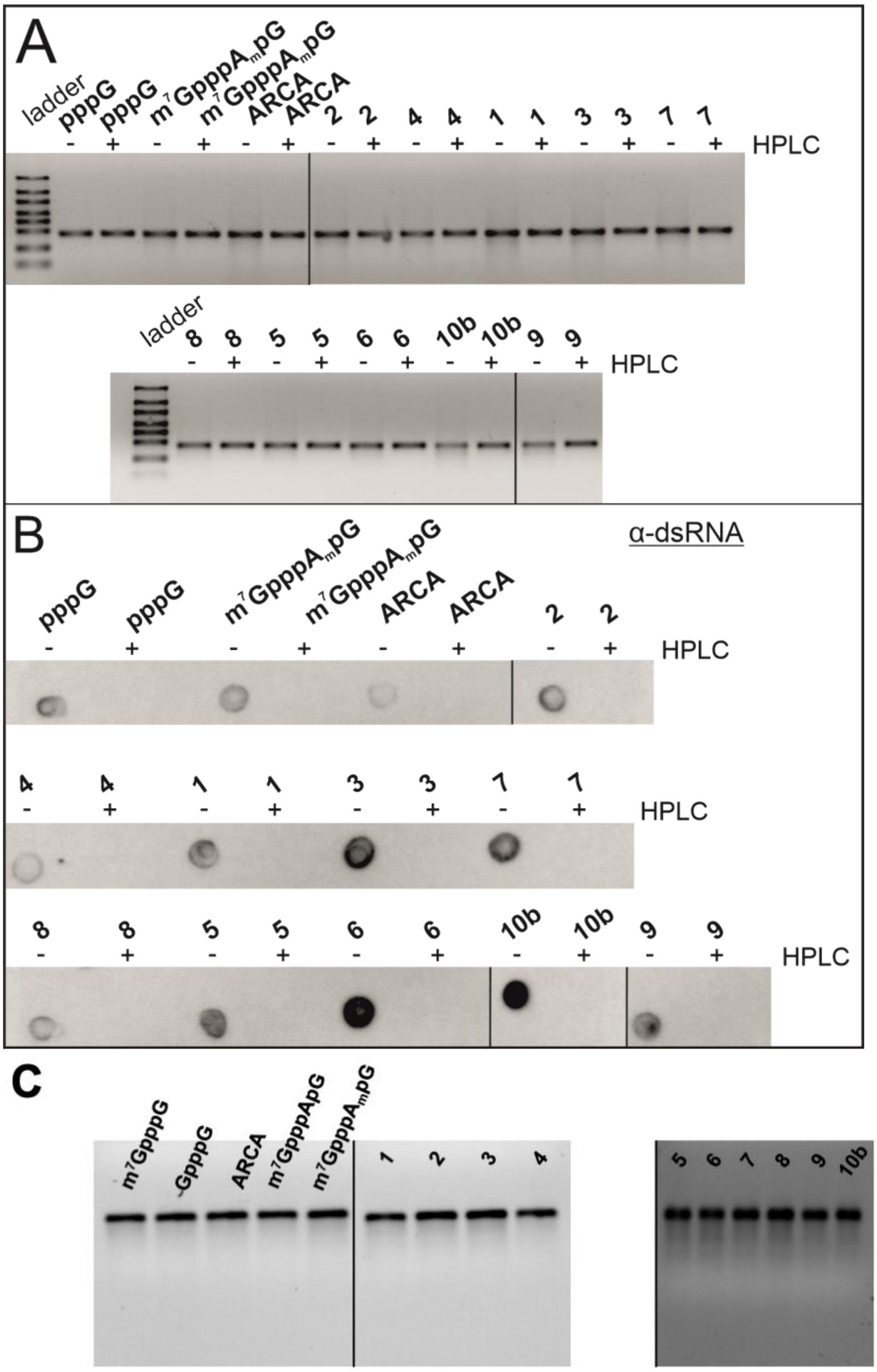

Figure S1. A. Representative 1.2% (TBE) agarose gels of capped RNA encoding *Gaussia* luciferase. B. Dot blot analysis 25 ng of mRNAs were analyzed as previously described in Sikorski et al. 2020[3] with modifications. C. Representative 1.2% (TBE) agarose gels of capped RNA encoding *Gaussia* luciferase. mRNAs were purified using a commercial kit and used in RRL experiments.

Figure S2. Luciferase activity in rabbit reticulocyte lysate

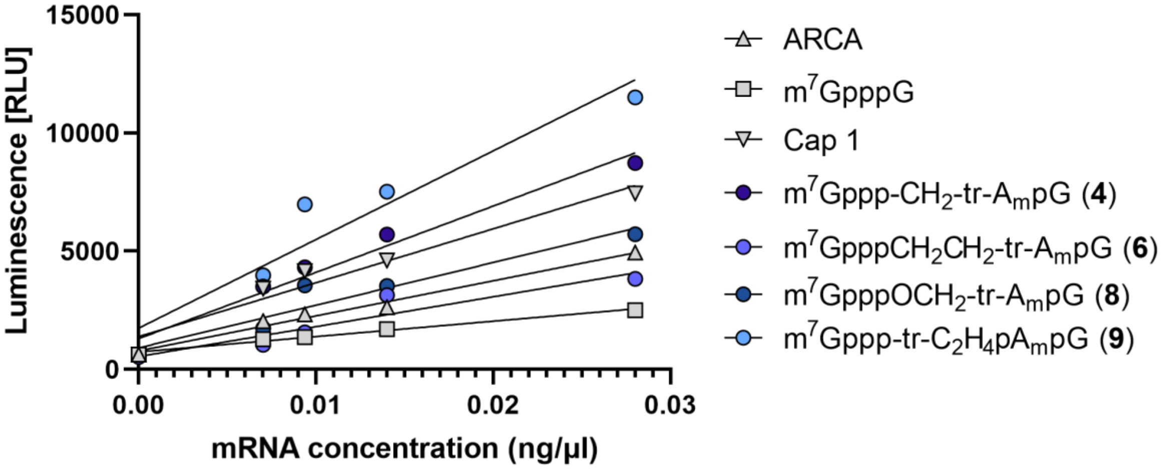

Figure S2. Luciferase activity produced by translation of mRNAs capped with various cap analogues in rabbit reticulocyte lysate – results of a single experiment.

Figure S3. Susceptibility to degradation by hDCP1/DCP2 complex.

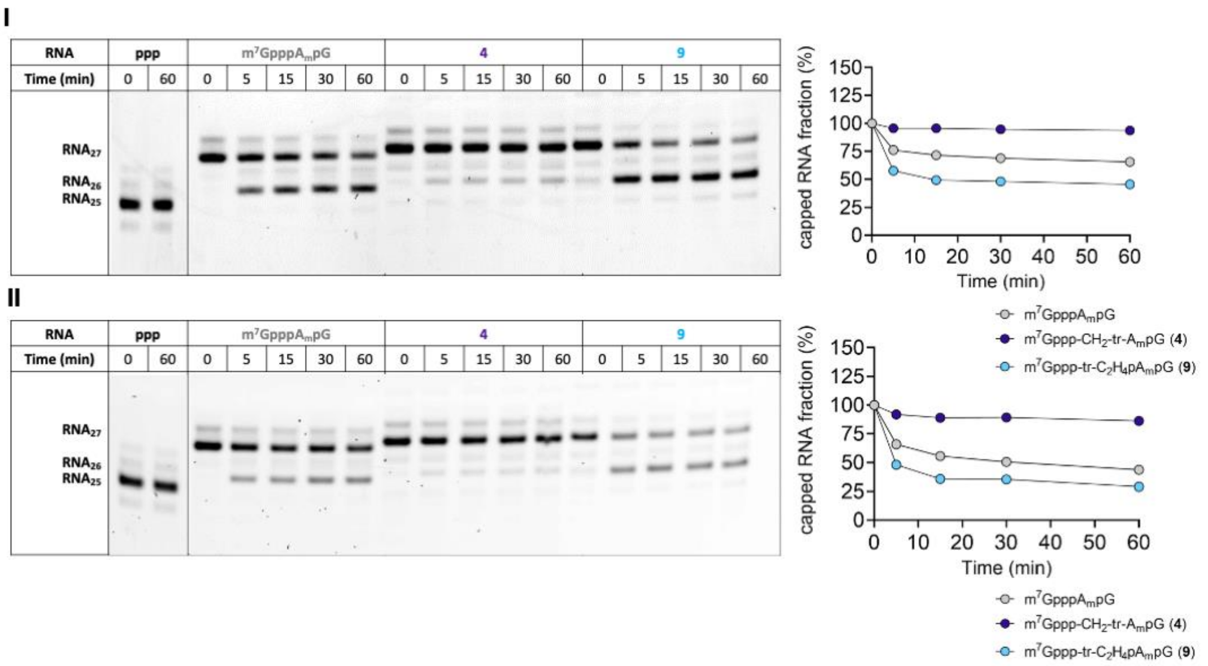

Figure S2. Susceptibility to degradation by hDCP1/DCP2 complex. Two separate repetitions. Experiment: 20 ng of capped RNA was subjected to 11 nM hDCP1/DCP2 in 50 mM Tris/HCl pH 8.0, 50 mM NH_4Cl, 0.01% Igepal with addition of 1 mM DTT, 5 mM $MgCl_2$ and 2 mM $MnCl_2$ at 37°C. Reactions were stopped at particular time points (5, 15, 30 and 60 min) and analysed by PAGE (15% acrylamide / 7 M urea / TBE gel) using SyberGold staining. Capped RNA fractions were calculated based on densitometric analysis of bands intensities.

RNA_{25} - uncapped RNA (control).

RNA_{27} - RNA capped with m^7GpppA_mpG, cap 4 or cap 9.

RNA_{26} - RNA decapped by hDCP1/DCP2 complex.

(1) Walczak, S.; Nowicka, A.; Kubacka, D.; Fac, K.; Wanat, P.; Mroczek, S.; Kowalska, J.; Jemielity, J. A novel route for preparing 5 ' cap mimics and capped RNAs: phosphate-modified cap analogues obtained via click chemistry. *Chemical Science* **2017**, *8* (1), 260-267, Article. DOI: 10.1039/c6sc02437h.
(2) Hebert, N.; Beck, A.; Lennox, R. B.; Just, G. A NEW REAGENT FOR THE REMOVAL OF THE 4-METHOXYBENZYL ETHER - APPLICATION TO THE SYNTHESIS OF UNUSUAL MACROCYCLIC AND BOLAFORM PHOSPHATIDYLCHOLINES. *Journal of Organic Chemistry* **1992**, *57* (6), 1777-1783, Article. DOI: 10.1021/jo00032a033.
(3) Sikorski, P. J.; Warminski, M.; Kubacka, D.; Ratajczak, T.; Nowis, D.; Kowalska, J.; Jemielity, J. The identity and methylation status of the first transcribed nucleotide in eukaryotic mRNA 5 ' cap modulates protein expression in living cells. *Nucleic Acids Research* **2020**, *48* (4), 1607-1626, Article. DOI: 10.1093/nar/gkaa032.
